# A simple test for the cleavage activity of customized endonucleases in plants

**DOI:** 10.1186/s13007-016-0118-6

**Published:** 2016-03-09

**Authors:** Nagaveni Budhagatapalli, Sindy Schedel, Maia Gurushidze, Stefanie Pencs, Stefan Hiekel, Twan Rutten, Stefan Kusch, Robert Morbitzer, Thomas Lahaye, Ralph Panstruga, Jochen Kumlehn, Goetz Hensel

**Affiliations:** Plant Reproductive Biology, Leibniz Institute of Plant Genetics and Crop Plant Research (IPK), Corrensstr. 3, 06466 Stadt Seeland/OT Gatersleben, Germany; Chair of Plant Breeding, Martin Luther University, Betty-Heimann-Str. 3, 06120 Halle (Saale), Germany; Structural Cell Biology, Leibniz Institute of Plant Genetics and Crop Plant Research (IPK), Corrensstr. 3, 06466 Stadt Seeland/OT Gatersleben, Germany; Unit of Plant Molecular Cell Biology, Institute for Biology I, RWTH Aachen University, Worringerweg 1, 52056 Aachen, Germany; ZMBP-General Genetics, University of Tübingen, Auf der Morgenstelle 32, 72076 Tübingen, Germany

**Keywords:** Transcription activator-like effector nucleases, RNA-guided endonucleases, Biolistic gene transfer, Site-directed mutagenesis, Transient expression

## Abstract

**Background:**

Although customized endonucleases [transcription activator-like effector nucleases (TALENs) and RNA-guided endonucleases (RGENs)] are known to be effective agents of mutagenesis in various host plants, newly designed endonuclease constructs require some pre-validation with respect to functionality before investing in the creation of stable transgenic plants.

**Results:**

A simple, biolistics-based leaf epidermis transient expression test has been developed, based on reconstituting the translational reading frame of a mutated, non-functional *yfp* reporter gene. Quantification of mutation efficacy was made possible by co-bombarding the explant with a constitutive *mCherry* expression cassette, thereby allowing the ratio between the number of red and yellow fluorescing cells to serve as a metric for mutation efficiency. Challenging either stable mutant alleles of a compromised version of *gfp* in tobacco and barley or the barley *MLO* gene with TALENs/RGENs confirmed the capacity to induce site-directed mutations.

**Conclusions:**

A convenient procedure to assay the cleavage activity of customized endonucleases has been established. The system is independent of the endonuclease platform and operates in both di- and monocotyledonous hosts. It not only enables the validation of a TALEN/RGEN’s functionality prior to the creation of stable mutants, but also serves as a suitable tool to optimize the design of endonuclease constructs.

**Electronic supplementary material:**

The online version of this article (doi:10.1186/s13007-016-0118-6) contains supplementary material, which is available to authorized users.

## Background

The exploitation of either customizable transcription activator-like effector nucleases (TALENs) [1, 2] or RNA-guided endonucleases (RGENs) [3, 4] has opened up numerous possibilities for site-directed mutagenesis and precise genome editing in plants. The gaps induced in the host’s DNA are repaired by either non-homologous end joining or by homology-directed repair. The former process is error-prone and so randomly introduces insertions and deletions (indels), while the latter, which exploits the respective locus of the sister chromatid or an artificially provided DNA that combines homology to the target site with an alteration of choice as repair template, is highly precise [5, 6]. The documented use of customized endonucleases in plants to date has largely involved *Arabidopsis thaliana*, *Nicotiana benthamiana*, maize or rice [7]. A few inherited TALEN- or RGEN-induced mutations have been reported in both barley [8, 9] and wheat [10]. Budhagatapalli et al. [11] have demonstrated the feasibility of homology-directed editing of barley at the cellular level. In mammalian cells, mutation frequencies of up to, respectively, 60 and 80 % have been achieved following the application of TALENs and RGENs [12].

Customizing endonucleases remains a somewhat empirical process, which would benefit from the development of a simple validation assay able to be carried out prior to transformation. The purpose of the present research was to establish a chimeric expression construct comprising a target sequence positioned upstream of a mutated, non-functional copy of *yfp* (encoding the cellular marker yellow fluorescent protein). The compromised *yfp* sequence features a frameshift mutation which can be reversed by the activity of a sequence-specific endonuclease; thus the frequency of cells producing YFP was expected to reflect the cleavage activity of the endonuclease. The assay has been deliberately designed to be usable with any customizable endonuclease platform in both di- and monocotyledonous plant species. One of the chosen targets in barley was the *MLO* gene, the product of which is associated with susceptibility to infection by *Blumeria graminis*, the causative fungal pathogen of powdery mildew; *mlo* alleles, which fail to produce a functional MLO protein, typically imbue the host with a durable, broad-spectrum resistance against the disease [13, 14].

## Results

### The test system in the dicotyledonous species tobacco

**Fig. 1:**
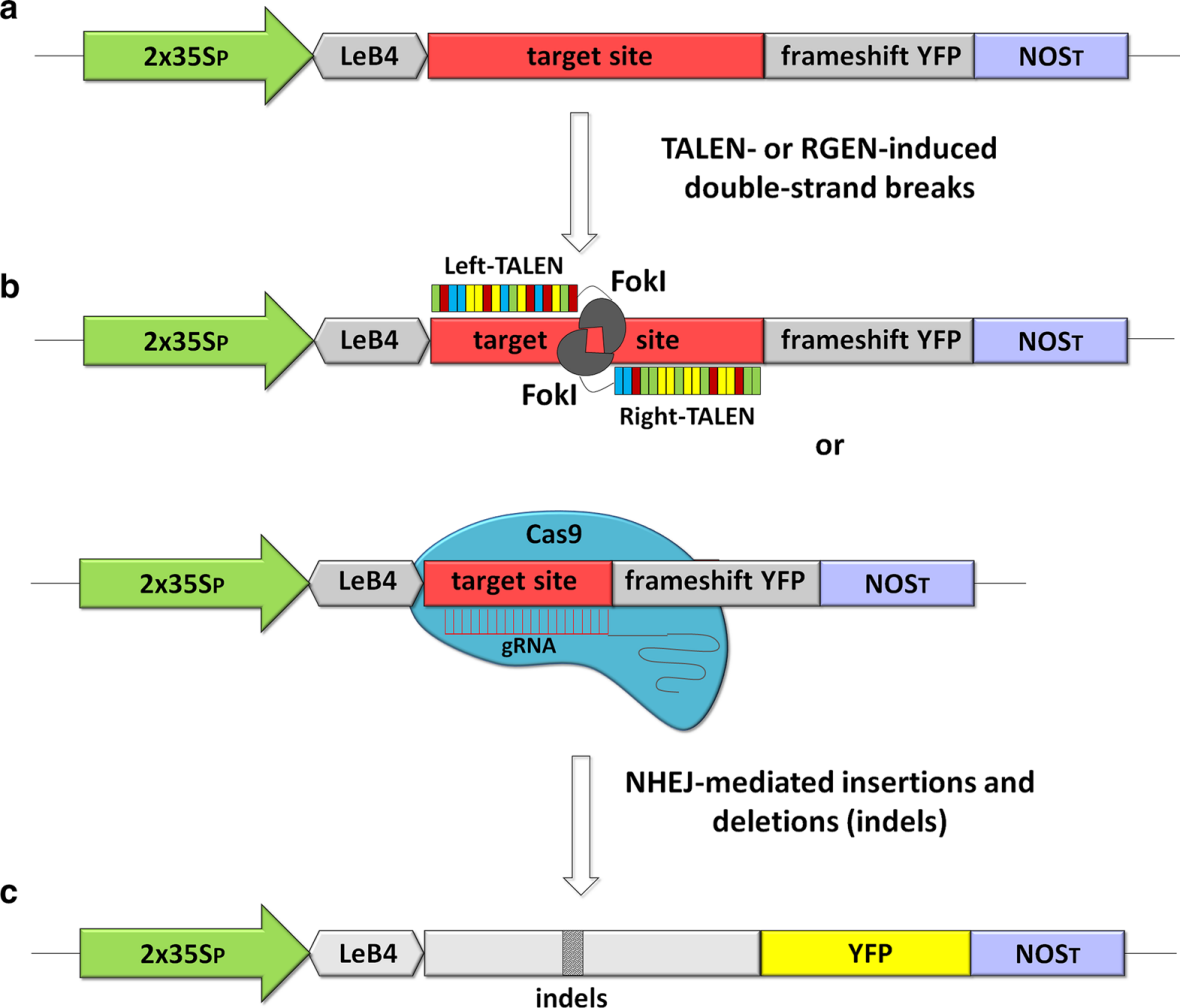
The principle of the transient expression system used to assess the relative cleavage activity of customized endonucleases. **a** The incorporation of a target site for sequence-specific endonucleases generates a frame shift in the *yfp* sequence. **b** Upon co-transformation of the target vector with TALENs or RGENs, double- strand DNA breaks at the target site are induced. **c** The imperfect repair of these breaks via non-homologous end-joining can restore the wild type reading frame, thereby leading to expression of *yfp* and the emission of a YFP signal. The elements shown are not drawn to scale. 2x35SP: doubled enhanced *CaMV 35S* promoter; LeB4: *Vicia faba* legumin B4 signal peptide; YFP: synthetic *yellow fluorescent protein* gene; NOST: *A. tumefaciens NOPALINE SYNTHASE* termination sequence; FokI: DNA cleavage domain of *Flavobacterium okeanokoites* type IIS restriction endonuclease; gRNA: guide-RNA; Cas9: *Streptococcus pyogenes* Cas9; TALEN: transcription activator-like effector nucleases; NHEJ: non-homologous end joining

**Fig. 2:**
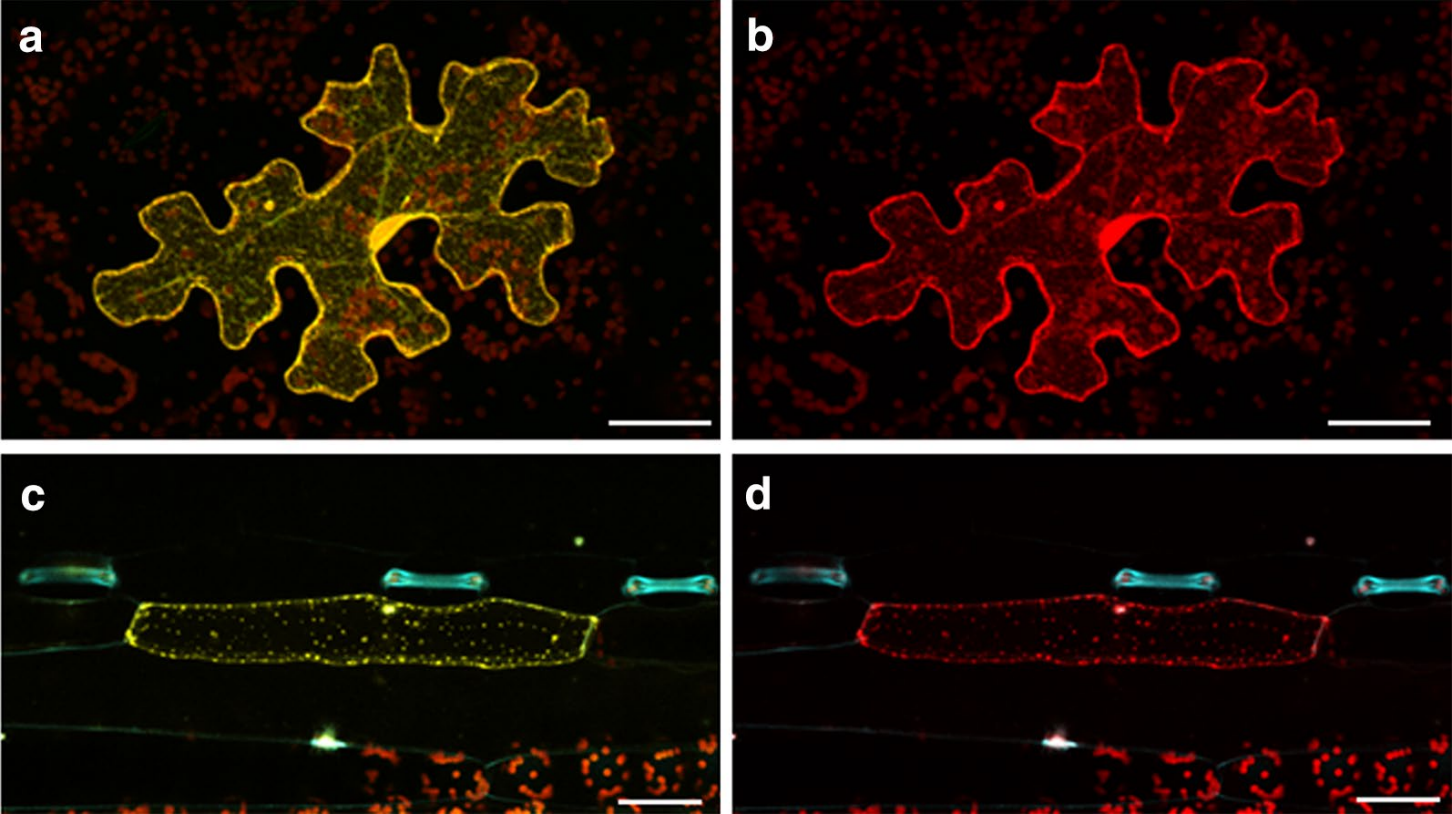
Induced mutations as detected by *yfp* expression. Representative epifluorescing transgenic **a**, **b** tobacco and **c**, **d** barley cells visualized 1 day after bombardment with a nuclease-specific vector together with a TALEN or RGEN; *mCherry* was co-transformed to allow quantification. *Bar* 50 µm

The principle of the assay is that the error-prone repair of endonuclease-induced double-strand breaks would generate indels at the target site. Consequently, a proportion of induced mutations were expected to restore the functionality of the compromised *yfp* sequence positioned downstream of the target sequence (Fig. 1). When tobacco (*Nicotiana tabacum* cv. SR1) leaf segments were co-bombarded with a nuclease-specific vector construct along with a TALEN or an RGEN construct, a number of epidermal cells began to accumulate YFP (Fig. 2a). The mutation frequency was quantified by co-bombarding a constitutive *mCherry* expression cassette; the ratio between the number of red and yellow fluorescing cells was then used to estimate the efficiency of the endonuclease construct (Fig. 2b). The co-bombardment of pTARGET-gfp1 with the pair of *gfp*-specific TALEN constructs (Table 1) generated 100 ± 71 yellow and 363 ± 148 red fluorescent cells, corresponding to a ratio of 27 %; meanwhile, the co-bombardment of pTARGET-gfp1 with the *gfp*-specific RGEN construct yielded a ratio of 75 % (Table 2). Bombardment with the pTARGET-gfp1 vector on its own failed to induce any cells to synthesize YFP.

**Table 1:** List of TALEN and RGEN target site sequences and experiments used

Construct	Target sequence	Species	Approach (constructs)
pTARGET-gfp1	TGGTGAACCGCATCGAGCTGAAGGGCATCGACTTCAAGGAGGACGGCAAGT	Tobacco	RGEN^t, st^ (pSI24)
			TALEN^t, st^ (pSP10, pSP11)
		Barley	TALEN^t, st^ (pGH297, pGH400)
pTARGET-gfp2	GTCTTTGCTCAGGGCGGACTGGG	Barley	RGEN^t^ (pSH92)
MLO pair #1	TGGTGCTCGTGTCCGTCCTCATGGAACACGGCCTCCACAAGCTCGGCCATGTA	Tobacco/barley	TALEN^t^ (p110/111)
MLO pair #2	TCCTCATGGAACACGGCCTCCACAAGCTCGGCCATGTAAGTCCCGTTACCCTA		TALEN^t^ (p112/113)
MLO pair #3	TGGAACACGGCCTCCACAAGCTCGGCCATGTAAGTCCCGTTACCCTAGCTCAA		TALEN^t^ (p114/115)
MLO pair #4	TGCTGGCTTTGTATGCAGATGGGATCAAACATGAAGAGGTCCATCTTCGACGA		TALEN^t^ (p124/125)
MLO pair #5	TGGCTTTGTATGCAGATGGGATCAAACATGAAGAGGTCCATCTTCGACGAGCA		TALEN^t^ (p126/127)
pTARGET-MLO	GCTGGAACACGGCCTCCACAAGCTCGGCCATGTAAGTCCCGTTACCCTAGCTCA	Barley	TALEN^t, st^ (p114/115)

*t* transient, *st* stable transgenic plants

**Table 2:** Relative cleavage activity of RGEN and TALEN constructs in transiently transformed barley and tobacco leaf explants

Plant species	Target gene	Type of customized endonuclease	Constructs used	Experiment	YFP cells	mCherry cells	Ratio YFP/mCherry cells (%)
Tobacco	*gfp*	RGEN	pTARGET-gfp1	1	273	371	73.6
			+ RGEN-gfp	2	342	389	87.9
				3	324	499	64.9
				Average	313 ± 29	420 ± 57	74.6
			pTARGET-gfp1	1	0	106	0
				2	0	204	0
				3	0	81	0
				Average	0	130 ± 65	0
		TALEN	pTARGET-gfp1	1	195	361	54.0
			+ TALEN-gfp	2	25	183	13.7
				3	80	545	14.7
				Average	100 ± 71	363 ± 148	27.5
Barley	*gfp*	RGEN	pTARGET-gfp2	1	72	207	34.8
			+ RGEN-gfp	2	55	206	26.7
				3	83	255	32.5
				Average	70 ± 12	223 ± 23	31.4
			pTARGET-gfp2	1	0	46	0
		TALEN	pTARGET-gfp1	1	71	223	31.8
			+ TALEN-gfp	2	110	331	33.2
				3	49	195	25.1
				Average	77 ± 25	250 ± 59	30.7
			pTARGET-gfp1	1	0	44	0
	*MLO*	TALEN	pTARGET-MLO	1	134	350	38.3
			+ TALEN-MLO	2	107	254	42.1
				3	112	237	47.3
				Average	118 ± 12	280 ± 50	42.0
			pTARGET-MLO	1	0	52	0

To estimate the frequency of mutations achievable in a stably transformed plant, a transgenic tobacco plant harboring a single copy of *gfp* [15] was co-transformed with the pair of *gfp*-specific TALEN constructs to produce plants carrying only one of the TALEN units; no mutations were detectable in the *gfp* sequence in either transgenic. The two TALEN units were then brought together by intercrossing the primary transgenics. Of the 35 progeny found to harbor both *gfp*-specific TALEN units (Table 3), one carried a mutated form of *gfp*. In a similar experiment based on the *gfp*-specific RGEN construct, 17 transgenics were obtained; of these, 15 carried *Cas9* and guide-RNA (gRNA), and of these, 12 contained mutations (Table 3). The mutations obtained were diverse in nature [15].

**Table 3:** Stable transgenic plants expressing RGEN or TALEN constructs

Plant species	Target gene	Type of customized endonuclease	PCR positive		Mutant plants	Ratio PCR+/mutants (%)
			*Cas9*/*FokI*	gRNA		
Tobacco	*gfp*	RGEN	17	15	12	80.0
		TALEN	35	n.a.	1	2.9
Barley	*gfp*	TALEN	66	n.a.	4	6.1
	*MLO*	TALEN	6	n.a.	3	50.0

*n.a.* not analysed

### The test system in the monocotyledonous species barley

Co-bombardment of barley (*Hordeum vulgare* cv. ‘Golden Promise’) leaves with pTARGET-gfp1 and the pair of *gfp*-specific TALEN constructs (Table 1) produced 77 ± 25 epidermal cells showing yellow (YFP) fluorescence and 250 ± 59 exhibiting red (mCherry) fluorescence (Table 2), yielding a ratio of 30.7 %. Bombardment with the *gfp*-specific RGEN construct and pTARGET-gfp2 led to a relative cleavage activity of 31.4 %. To validate the functionality of TALENs targeting an endogenous barley gene, a further co-bombardment of the same barley material was conducted using the *MLO*-specific pTARGET-MLO reporter plasmid and the appropriate TALEN pair #3: this resulted in a relative cleavage activity of 42.0 % (Table 2). The *MLO*-specific TALEN constructs were also tested in combination with the *GUS* reporter gene in barley (cv. ‘Ingrid’; using bombardment) and in *N. benthamiana* (using *Agrobacterium tumefaciens*-mediated transient expression) using five different TALEN pairs targeting two different exons of the *MLO* gene. Out of the five tested TALEN pairs (Additional file 1), only TALEN pairs #2 and #3 showed detectable activity and restored *GUS* expression when the TALEN constructs were co-transformed with the respective reporter construct (Additional file 2). While TALEN pair #2 yielded only very few GUS-positive cells in barley and *N. benthamiana*, TALEN pair #3 was more active and reproducibly led to GUS-positive cells in both plant systems (Additional file 3). Note that in the case of this assay, there was no means of normalizing the transformation efficiency due to the lack of a second reporter.

**Fig. 3:**
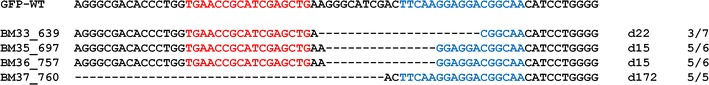
Alignment of mutated *gfp* sequences recovered from four barley transformants (BM33_639, BM35_697, BM36_757, and BM37_760) induced by the presence of a TALEN pair. *gfp*-specific PCR products were subcloned and up to ten clones were sequenced. The sequences shown in *red* and *blue* represent, respectively the *left* and *right* TALEN binding sites; the number of nucleotides deleted (*dashes*) and the number of mutants/number of clones analysed is shown to the right of each sequence

**Fig. 4:**
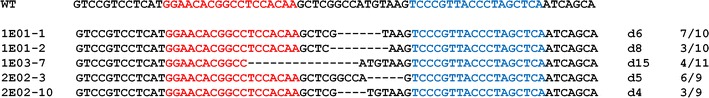
Alignment of mutated *MLO* sequences recovered from three barley transformants (1E01, 1E03, and 2E02) induced by the presence of a TALEN construct. *MLO*-specific PCR products were subcloned and up to ten clones were sequenced. The sequences shown in *red* and *blue* represent, respectively the *left* and *right* TALEN binding sites; the number of nucleotides deleted (*dashes*) and the number of mutants/number of clones analysed is shown to the right of each sequence

To assess the efficacy of the TALEN constructs in a stably transformed barley line, a transgenic version of cv. ‘Igri’ harboring *gfp* [8] was re-transformed. TALEN-induced mutations in *gfp* were detected in four out of 66 T_0_ plants (Table 3; Fig. 3). The induced mutations included various deletions in the size range of 15–172 nt. Therefore, one can conclude mono-allelic mutations for the *gfp* gene. When the *MLO*-specific TALEN construct was transformed into cv. ‘Golden Promise’, three of the six regenerants included a deletion in *MLO*: one harbored a single 15 nt deletion, while clones derived from the other two harbored a range of 4–8 nt deletions (Fig. 4). While two plants were considered bi-allelic mutants, in one *MLO* mutant plant, also wild-type alleles were detectable leading to the conclusion of a mono-allelic alteration.

## Discussion

Although endonuclease-enabled site-directed mutagenesis is known to be effective in a range of plant species [7], the tools presently available to aid the in silico design of binding modules (Target Finder [16], Talvez and Storyteller [17, 18], and TALgetter [19]) or gRNAs (CRISPR design [20], CRISPRer [21] and Deskgen [22]) do not consistently produce the desired outcome [23]. There is thus clearly potential for optimization based on reshuffling the current endonuclease systems, while entirely novel systems are also emerging (such as the Cpf1, see [4]). Here, a convenient platform for detecting the cleavage activity of an endonuclease construct was elaborated, based on the restoration of function to a compromised version of *yfp*, a gene which encodes the readily assayable yellow fluorescent protein. The principle of customized endonuclease-induced restoration of reporter gene function has been used previously in the context of transient expression via infiltration of leaves using Agrobacterium, which is amenable only for a limited number of plant species [24, 25]. By contrast, the method presented here relies on particle bombardment and can thus be readily adopted in any plant species. An additional novel feature which was included was co-transformation with *mCherry*, in order to allow for the comparative quantification of mutation frequency induced by the TALEN/RGEN construct. The concept of using two fluorescent reporters is related to a previously established assay system designed to assess the efficiency of gene silencing constructs [26].

The tobacco TALEN transient expression experiment established that around one third of the cells showing red fluorescence (*mCherry* expressing) also generated a YFP signal (Fig. 2; Table 2), while this frequency was raised to about 75 % when the RGEN construct was assayed (Table 2). The higher success rate achieved with the RGEN construct accords with the literature, and has been at least partly explained by the prediction that in contrast to TALENs, which exhibit a broad range of mutational events, most of the mutations induced by RGEN activity are 1 nt insertions [27, 28]. Consequently, the three possible reading frames of the reporter gene are unlikely to occur in balanced proportions in the latter platform. In the case of the *gfp*-RGEN approach in tobacco, the preferentially occurring insertion of one nucleotide restores the *yfp* open-reading-frame and therefore, the comparatively high score achieved here is in accordance with the literature. By contrast, the *gfp*-RGEN approach in barley uses a different target site and in this case frame shifts by 1 nt back or 2 nt forward cause a functional *yfp*, which is not expected to occur as frequently as the shift by 1 nt forward. In general, it is clear that comparisons of the efficacy of multiple nucleases based on a single target gene are only possible if the same frame-shift is used for the *yfp* coding sequence succeeding the respective target motifs.

**Fig. 5:**
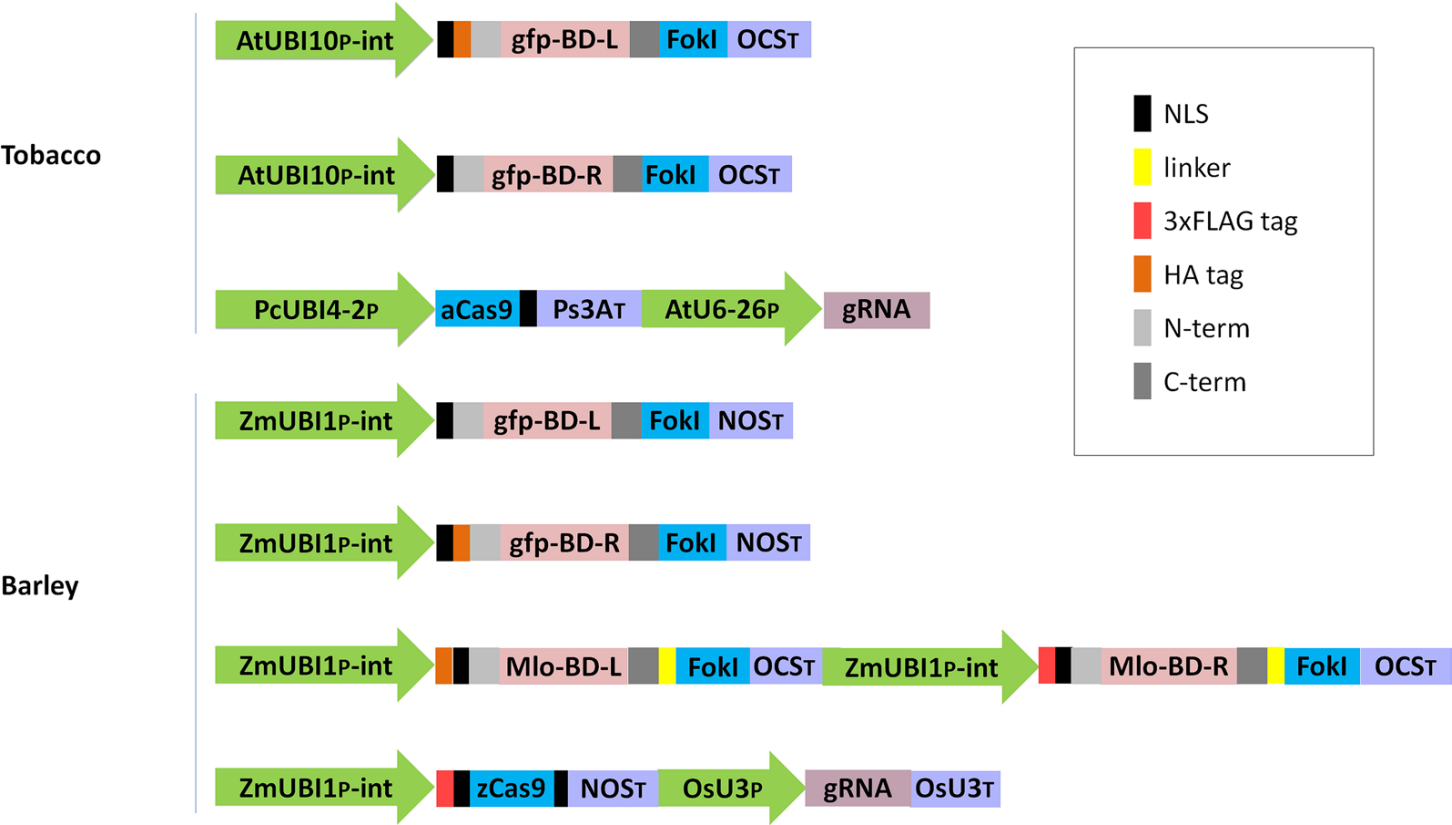
The structure of the customized endonuclease constructs. AtUBI10P-int: *A. thaliana* *UBIQUITIN*-*10* promoter with intron; gfp-BD-L/R: synthetic S65T green fluorescent protein left/right TALEN binding domain; FokI: FokI cleavage domain; OCST: *A. tumefaciens* *OCS* terminator; PcUBI4-2P: *Petroselinum crispum UBIQUITIN4*-*2* promoter; aCas9: *A. thaliana* codon-optimzed *Cas9*; Ps3AT: pea *Pea3A* terminator; AtU6-26P: *A. thaliana U6*-*26* promoter; gRNA: guide-RNA; ZmUBI1P-int: maize *UBIQUITIN1* promoter with first intron; NOST: *NOS* terminator; NLS: SV40 Simian virus 40 nuclear localization signal; 3xFLAG tag: multimeric synthetic FLAG octapeptide; HA tag: hemagglutinin tag; N-term: N-terminus of a modified version of *Xanthomonas campestris* pv. *vesicatoria* *AvrBs3*; C-term: C-terminus of a modified version of *AvrBs3*. The elements shown are not drawn to scale

The induction of mutations to *gfp* induced by stably incorporated TALEN and RGEN constructs corroborated the behavior shown in the transient expression assays. While only a low mutation frequency (2.9 %) was achieved using the *gfp*-specific TALENs, 80 % of the primary transgenic tobacco plants harboring the *gfp*-specific RGEN-encoding sequence contained indels in the target sequence (Table 3). Nekrasov et al. [29] have reported a mutation frequency of 6.6 % in the *N. benthamianaPHYTOENE DESATURASE* (*PDS*) sequence by co-expressing *Cas9* and gRNA. At the same time, Li et al. [30] have shown that both the expression level of the gRNA and the size of the ratio of Cas9 to gRNA are important determinants of mutagenic potential. A very high efficiency (>84 %) has been claimed in RGEN experiments involving *N. tabacumPDS* or *PLEIOTROPIC DRUG RESISTANCE6* (*PDR6*), in which Cas9 and gRNA were presented within a single expression vector [31]. The efficiency of RGEN-mediated mutagenesis is also thought to depend on the accumulation of Cas9 protein. In tobacco, the mutation frequency was reduced when the human codon-optimized variant of *Cas9* was employed [29], while Gao et al. [31] used a tobacco codon-optimized version. Here, a single expression vector was designed on the basis of the *A. thaliana* codon-optimized *Cas9* [32], and this induced a high mutation frequency.

When the system was applied to the more refractory monocotyledonous species barley, the same choice of target gene (*gfp*) was made, since it has been established that *gfp*-specific TALENs are effective inducers of heritable mutations in cultured barley cells [8]. As in tobacco, co-bombardment of pTARGET-gfp1 with the two TALEN units led to the induction of *yfp* expression in about a third of the red fluorescing cells (Table 2). In contrast to tobacco though, the cleavage activities of the *gfp*-specific TALEN and RGEN constructs were identical (Table 2). The comparatively poor performance of the latter in barley indicates that different target sites may have different accessibility for the endonucleases. The generation of stable *gfp* mutants via the transformation of immature embryos proved to be less efficient than that achievable from embryogenic pollen cultures [8], possibly because the effectiveness of the maize *UBI1* promoter is cell-type dependent. The stable generation of *mlo* mutants corroborated the high TALEN activity detected in the transient expression assay. The *MLO*-specific TALEN pair was almost as efficient as the *gfp*-specific RGEN construct in tobacco (Table 3), clearly showing that a pre-validation of TALEN constructs on the basis of their transient expression is an effective procedure, because only one out of five tested *MLO*-specific TALEN constructs showed activity in a reproducible manner (Additional file 3).

The differences between transient expression and stable transgenic plants may be assigned to the fact that expression strength strongly depends on the number of expression units present within a given cell (gene dosage), the specificity and strength of the promoter driving the transgene, the cell type and developmental stage as well as further conditions, which differ in approaches involving different gene transfer methods and target tissues such as barley and tobacco epidermal cell layers and immature embryos, which were used in the present study. In addition, the *gfp*-TALENs were driven by another *ZmUBI1* promoter version than the one used in the *MLO*-TALEN and *gfp*-RGEN constructs.

## Conclusions

In summary, a convenient *in planta* test system for the cleavage activity of sequence-specifically customized endonucleases was established and exemplified using TALENs and RGENs. It is applicable in both dicot and monocot plant species, which makes it a universal tool for the plant science community. This system may not only facilitate the validation of endonuclease functionality prior to the generation of stable mutant plants but also enable researchers to study general principles of endonuclease activity and to optimize construct design.

## Methods

### Plant material

Four week old seedlings of wild type tobacco (*N. tabacum* cv. SR1) were used for the transient transgene expression experiments. In the stable transformation experiment, seeds of TSP20L1-1, an established transgenic line harboring a single copy of *gfp* [15], were surface-sterilized and germinated on [33] solid medium for 2 weeks, after which the seedlings were transferred to culture boxes (107 × 94 × 96 cm) for a further 4 weeks. Barley (*H. vulgare*) cvs. ‘Golden Promise’ and ‘Ingrid’ were used for the transient transgene expression experiments and cv. ‘Golden Promise’ for the mutation of *MLO*, while a transgenic line of cv. ‘Igri’ was used for the *gfp*-specific TALEN experiment; the latter’s seedlings required vernalization (8 weeks at 4 °C under a 9 h photoperiod).

### Transient expression test vector construction

Details of the cloning steps, based on standard procedures, plasmid maps and sequences (Additional file 6), primers and functional elements, are provided in Additional files 4 and 5, and Fig. 5. The generic vector pNB1 (GenBank: KU705395) carries a modified *yfp* reporter gene [11] driven by a doubled enhanced *CaMV 35S* promoter [34]; it includes a *Bam*HI and an *Eco*RI cloning site between the sequences encoding the pea *Legumin B4* signal peptide, *yfp* and a C-terminal KDEL motif for protein retention in the endoplasmic reticulum. Nuclease-specific vectors were developed by inserting 20–50 bp target motifs (annealed oligos, Additional file 4) into the *Bam*HI and *Eco*RI sites to generate the constructs pTARGET-MLO, pTARGET-gfp1 and pTARGET-gfp2 (Additional file 6).

### TALEN vector construction

The *gfp*-specific TALEN vectors [8] comprise a left and a right TALEN unit, each driven by the maize *UBIQUITIN1* promoter [35], along with the bialaphos resistance-conferring *BAR* gene driven by a doubled enhanced *CaMV35S* promoter. The *gfp*-specific TALEN sequences used in the tobacco constructs were identical to those used in barley [8]. However, unlike in the barley constructs, in tobacco the left and right TALEN units were introduced into pUbiAt-OCS (DNA-Cloning-Service, Hamburg, Germany), allowing them to be driven by the *A. thaliana UBIQUITIN*-*10* promoter; their terminal sequence was from the *A. tumefaciens OCTOPINE SYNTHASE* (*OCS*) gene. Each TALEN expression cassette was introduced into the p6N vector via its *Sfi*I cloning sites, which harbors the *HYGROMYCIN PHOSPHOTRANSFERASE* (*HPT*) gene driven by the *A. tumefaciens NOPALINE SYNTHASE* promoter. The pSP10 (left TALEN unit) and pSP11 (right TALEN unit) vectors (Additional file 6) were introduced into *A. tumefaciens* strain GV2260 using a heat shock protocol.

The *MLO*-specific TALEN effector binding elements were preceded by a T, 18 bp long, and separated by a 15 nt spacer sequence (Additional file 1). Repeat lacking TALEN units (pICH47732 TALEN∆Rep and pICH47742 TALEN∆Rep) were assembled with *Bsa*I site-flanked modules encoding a truncated *CaMV 35S* promoter (pICH51277, see [36]), HA-NLS [37], a truncated TALE N- and C-terminus, *FokI* [38] and *OCS* terminator (pICH41432, [36]) into pICH47732 and pICH47742 [36], respectively. The repeat domains of TALEN 114 and TALEN 115 were created following Morbitzer et al. [39] and were cloned into the *Bpi*I site of pICH47732 TALEN∆Rep and pICH47742 TALEN∆Rep. TALEN modules with repeats (pICH47732 TALEN 114 and pICH47742 TALEN115) were assembled together with pICH47744 [36] into pUC57_BpiI/KpnI_shuttle via *Bpi*I cut-ligation and thereby flanked by *Kpn*I. Both of the TALEN *Kpn*I fragments were introduced into the *Kpn*I site of the p6int vector (DNA-Cloning-Service, Hamburg, Germany). The TALEN encoding T-DNA vector used for the *GUS* reporter system was assembled via *Bpi*I cut-ligation from pICH47732 TALEN 114, pICH47742 TALEN 115, pICH47751 Kanamycin, a vector which confers resistance to kanamycin, and pICH47766 [36] into pICH50505 [36] (details and sequences given in Additional file 5).

### RGEN vector construction

To generate a monocotyledonous species-specific generic RGEN vector, two *Sfi*I restriction sites were first inserted into pBUN411 [40]. In addition the *BAR* gene was removed to produce pSH91. The *gfp*-specific RGEN vector pSH92 was generated by replacing the spectinomycin resistance gene via *Bsa*I digestion with a synthetic DNA fragment containing a *gfp*-specific protospacer, formed by annealing the partially complementary oligonucleotides GFP_PP1_f and GFP_PP1_r (Additional file 4). For the tobacco *gfp*-specific RGEN, the Gateway^®^-compatible *Cas9* expression system [32] was used. The above-mentioned *gfp*-specific protospacer sequence was introduced into pEN-Chimera via the pair of *Bbs*I sites. The resulting construct, driven by the *A. thaliana U6*-*26* promoter, was then transferred into pDe-CAS9 via a single site Gateway^®^ LR reaction [32], ensuring that *Cas9* lay under the control of the *Petroselinum crispumPcUbi 4*-*2* promoter and the pea *Pea3A* terminator sequence. The resulting pSI24 vector (Additional file 6) was introduced into *A. tumefaciens* strain GV2260 using a heat shock protocol.

### Transient transgenesis via particle bombardment

Barley and *N. tabacum* leaf explants were transiently transformed using a PDS-1000/He Hepta^TM^ device equipped with a 1100 psi rupture disc (Bio-Rad, Munich, Germany). For barley, six primary leaves harvested from 7 to 8 day old seedlings were placed adaxial side up on 1 % agar containing 20 µg/mL benzimidazol and 20 µg/mL chloramphenicol. For *N. tabacum*, a single leaf harvested from a 4 week old plant was placed on solidified (0.8 % agar) Murashige and Skoog [33] medium containing 2 % sucrose and 400 mg/L ticarcillin. A 7 µg aliquot of plasmid DNA was mixed with 3 mg gold micro-carriers by vortexing in the presence of 25 µL 25 mM CaCl_2_ and 10 µL 0.1 M spermidine. After centrifugation, the pellet was washed with 75 and 100 % ethanol, followed by suspension in 60 µL 100 % ethanol. A total of 4 µL of coated micro-carrier suspension was loaded onto each of the seven macro-carriers, as recommended by the PDS-1000/He manual. Each set of explants were bombarded twice with a total amount of 16–19 µg plasmid DNA (7 µg nuclease test vector, 7 µg endonuclease vector and 2–5 µg mCherry vector), then incubated at room temperature for 1 day before assaying for fluorescence. Each experiment was carried out three times.

### Transient transgenesis via *Agrobacterium tumefaciens* infiltration

The *A. tumefaciens* strain GV3101 pMP90RK [41] was used for transient expression assays in *N. benthamiana*. Liquid culture-grown (28 °C, 180 rpm for 1 day) *Agrobacterium* was set to an OD_600_ of 1.0 with infiltration solution (10 mM MgCl_2_, 10 mM MES (pH 5.7), 200 μM acetosyringone). The bacteria were delivered into 3–4 week-old *N. benthamiana* leaves using a needleless syringe. After 2 days, infiltrated areas were cut out and de-stained in 80 % (v/v) ethanol for a few days prior to GUS staining (see below).

### Stable transformants of barley and tobacco

Barley was transformed according to Hensel et al. [42], except that the immature embryos harvested from transgenic single-copy, *gfp* expressing cv. ‘Igri’ were initially cultured for 5 days on BPCM (solid BCIM, 5 mg/L dicamba) before the introduction of *A. tumefaciens*. Tobacco (*N. tabacum* wild type or line TSP20L1-1) plants were transformed with pGH292 or pSI24, respectively. This vector harbors *gfp* controlled by the *A. thaliana UBIQUITIN*-*10* promoter and the *A. tumefaciens OCS* terminator. The *NEOMYCIN PHOSPHOTRANSFERASE* gene (*NptII*) for kanamycin resistance *in planta* is driven by the *CaMV 35S* promoter and termination sequence. The transgene was introduced into *A. tumefaciens* strain GV2260 using a heat shock protocol. Leaf sections (~1 cm^2^) excised from sterile-grown plants were laid on Murashige and Skoog [33] medium containing 3 % w/v sucrose, 1 mg/L 6-benzylaminopurine, 0.1 mg/L 1-naphthalene acetic acid and 2 % agar for 1–2 days, before inoculation with the transgenic *A. tumefaciens* for 30 min. The explants were blotted with sterile filter paper and kept for 3 days at 19 °C in the dark, on medium supplemented with 400 mg/L ticarcillin and either 100 mg/L kanamycin or 5 mg/L bialaphos at 22 °C. Developing calli were sub-cultured every 10 days. After emergence of first shoots, the plates were transferred to light (16 h photoperiod) until shoots had reached 1 cm in length, at which point these were excised and placed on Murashige and Skoog [33] medium containing 2 % w/v sucrose, 0.8 % agar to stimulate root initiation. Plantlets which had developed a viable root system were transferred to the greenhouse.

### Genomic DNA isolation and PCR

Genomic DNA was isolated from snap-frozen leaves following Palotta et al. [43]. Subsequent 20 μL PCRs were formulated with 50–100 ng template DNA, and primed as listed in Additional file 4. The reaction products were purified using a QIAquick PCR Purification kit (Qiagen, Hilden, Germany) to allow for amplicon sequencing. Target-specific PCR products amplified from transgenic individuals were cloned into pGEM-T Easy (Promega, Mannheim, Germany). After blue-white selection, plasmid DNA was isolated from ten positive clones and sequenced.

### Confocal microscopy

Frequency of TALEN or RGEN construct induced mutations was determined from the ratio between the number of yellow-fluorescent (YFP) and red-fluorescent (mCherry) cells. For this a total of six leaves of barley and one leaf of *N. tabacum* were analyzed with a Zeiss LSM780 confocal laser microscope (Carl Zeiss, Jena, Germany). YFP fluorescence was visualized using a 514 nm laser line in combination with a 517–560 nm bandpass; mCherry fluorescence was visualized with a 561 nm laser line in combination with a 570–620 nm bandpass.

### GUS staining

Barley and *N. benthamiana* leaves were harvested for GUS staining three and 2 days, respectively, after bombardment/or *Agrobacterium* infiltration. The leaves were submerged in 5-bromo-4-chloro-3-indolyl-ß-d-glucoronide cyclohexylammonium (X-Gluc) staining solution (42.3 mM NaH_2_PO_4_, 57.7 mM Na_2_HPO_4_, 10 mM EDTA, 20 % methanol, 5 mM K_3_Fe(CN)_6_, 5 mM K_4_Fe(CN)_6_ × 3 H_2_O, 1 mg/mL X-Gluc, 0.1 % Triton X-100) and vacuum was applied three times for 10 min. Then, the material was incubated at 37 °C overnight. Afterwards, the leaves were bleached in 80 % EtOH at room temperature for at least 2 days. The leaves were screened for blue-stained cells by bright field microscopy; photographs were taken with the Keyence Biorevo BZ9000 microscope (Keyence Corporation, Neu-Isenburg, Germany).

## Additional files

10.1186/s13007-016-0118-6 *MLO*-specific TALEN target sequences.
10.1186/s13007-016-0118-6 (a) Step-wise functional principle of transient expression vector system for assessing the relative cleavage activity of customized endonucleases. Incorporation of a target site for sequence-specific endonucleases deliberately generates a frame shift in the codon sequence of *GUS*. Upon co-transformation of target vector along with respective customized TALENs, double-strand breaks at the target site are induced. The repair of double-strand breaks at target site via non-homologous end-joining, which often introduces indels, render *GUS* back in frame and GUS protein can be detected by X-Gluc staining. (b) Example for successful employment of the reporter system in a transient assay in barley. From left to right: negative control (reporter construct only) and two examples of successful induction of indels indicated by blue-green GUS staining of the cell. Upper panel: barley (*Hordeum vulgare* cv. ‘Ingrid’, *Hv*) after bombardment; lower panel: *N. benthamiana* (*Nb*) after *Agrobacterium* infiltration. Arrows highlight some GUS-stained cells. Bars: 50 µm (upper panel); 100 µm (lower panel).
10.1186/s13007-016-0118-6 Transient expression test of *MLO*-specific TALEN constructs in barley (using bombardment) and *N. benthamiana* (using agroinfection).
10.1186/s13007-016-0118-6 Oligomers used to clone pTARGET and RGEN plasmids, and the PCR-based analysis of putative transgenic regenerants.
10.1186/s13007-016-0118-6 Details and sequences for the *MLO*-specific TALEN constructs.
10.1186/s13007-016-0118-6 Plasmid maps and sequences of the constructs.
