# Supplementary material for: A simple test for the cleavage activity of customized endonucleases in plants
Source: Plant Methods. 2016 Mar 9;12:18. doi: 10.1186/s13007-016-0118-6 (PMC4784412; doi:10.1186/s13007-016-0118-6)
Supplement: Supplementary file 1 — 10.1186/s13007-016-0118-6 MLO-specific TALEN target sequences. [file 13007_2016_118_MOESM1_ESM.pdf]

Additional file 1: *MLO*-specific TALEN target sequences.

TALEN pair	Name	Sequence
**#1**	TALEN 110 EBE	T GGTGCTCGTGTCCGTCCT
	TALEN 111 EBE	T ACATGGCCGAGCTTGTGG
**#2**	TALEN 112 EBE	T CCTCATGGAACACGGCCT
	TALEN 113 EBE	T AGGGTAACGGGACTTACA
**#3**	TALEN 114 EBE	T GGAACACGGCCTCCACAA
	TALEN 115 EBE	T TGAGCTAGGGTAACGGGA
**#4**	TALEN 124 EBE	T GCTGGCTTTGTATGCAGA
	TALEN 125 EBE	T CGTCGAAGATGGACCTCT
**#5**	TALEN 126 EBE	T GGCTTTGTATGCAGATGG
	TALEN 127 EBE	T GCTCGTCGAAGATGGACC
