# Supplementary figures and images for: A simple test for the cleavage activity of customized endonucleases in plants
Source: Plant Methods. 2016 Mar 9;12:18. doi: 10.1186/s13007-016-0118-6 (PMC4784412; doi:10.1186/s13007-016-0118-6)

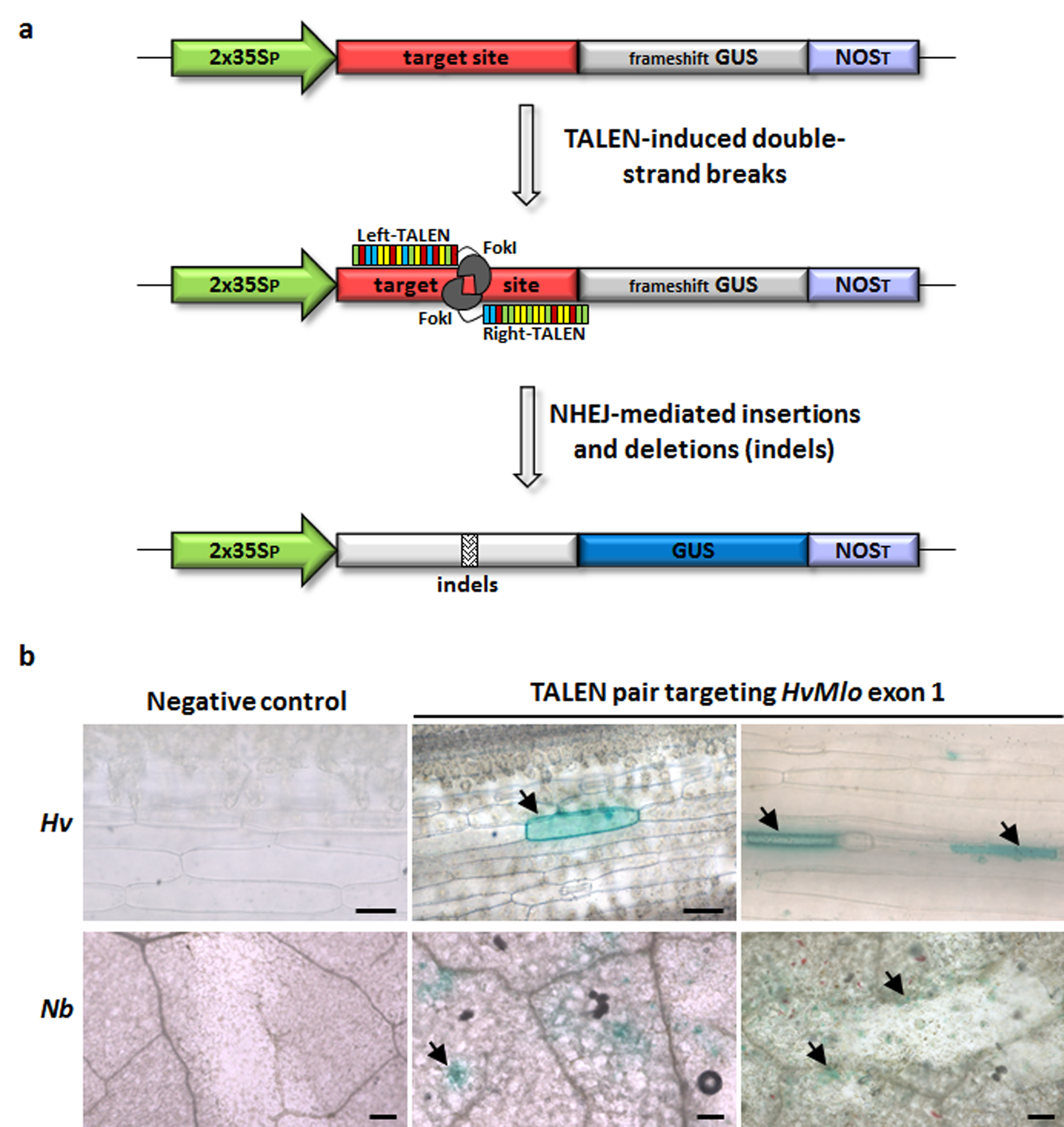

Supplement: Supplementary file 2 — 10.1186/s13007-016-0118-6 (a) Step-wise functional principle of transient expression vector system for assessing the relative cleavage activity of customized endonucleases. Incorporation of a target site for sequence-specific endonucleases deliberately generates a frame shift in the codon sequence of GUS. Upon co-transformation of target vector along with respective customized TALENs, double-strand breaks at the target site are induced. The repair of double-strand breaks at target site via non-homologous end-joining, which often introduces indels, render GUS back in frame and GUS protein can be detected by X-Gluc staining. (b) Example for successful employment of the reporter system in a transient assay in barley. From left to right: negative control (reporter construct only) and two examples of successful induction of indels indicated by blue-green GUS staining of the cell. Upper panel: barley (Hordeum vulgare cv. ‘Ingrid’, Hv) after bombardment; lower panel: N. benthamiana (Nb) after Agrobacterium infiltration. Arrows highlight some GUS-stained cells. Bars: 50 µm (upper panel); 100 µm (lower panel). [file 13007_2016_118_MOESM2_ESM.tif]
