# Supplementary material for: A simple test for the cleavage activity of customized endonucleases in plants
Source: Plant Methods. 2016 Mar 9;12:18. doi: 10.1186/s13007-016-0118-6 (PMC4784412; doi:10.1186/s13007-016-0118-6)
Supplement: Supplementary file 3 — 10.1186/s13007-016-0118-6 Transient expression test of MLO-specific TALEN constructs in barley (using bombardment) and N. benthamiana (using agroinfection). [file 13007_2016_118_MOESM3_ESM.pdf]

Additional file 3: Transient expression test of *MLO*-specific TALEN constructs in barley (using bombardment) and *N. benthamiana* (using *A. tumefaciens*-mediated transient expression).

**TALEN pair**	***MLO* target region**	***Nuclease activity***	
		**Barley**	***N. benthamiana***
#1	Exon 1	no	no
#2	Exon 1	weak	weak
#3	Exon 1	medium	medium
#4	Exon 3	no	no
#5	Exon 3	no	no
