# Supplementary material for: A simple test for the cleavage activity of customized endonucleases in plants
Source: Plant Methods. 2016 Mar 9;12:18. doi: 10.1186/s13007-016-0118-6 (PMC4784412; doi:10.1186/s13007-016-0118-6)
Supplement: Supplementary file 4 — 10.1186/s13007-016-0118-6 Oligomers used to clone pTARGET and RGEN plasmids, and the PCR-based analysis of putative transgenic regenerants. [file 13007_2016_118_MOESM4_ESM.pdf]

Additional file 4: Oligos used for the cloning of pTARGET and RGEN plasmids and PCR analysis of putative transgenic regenerants.

Primer	Direction	Sequence 5' – 3'	Primer binding site
RGEN-gfp target barley	forward	GATCGTCTTTGCTCAGGGCGGACTGGGC	Bp 611-633 of *gfp* gene, complementary strand
	reverse	AATTGCCCAGTCCGCCCTGAGCAAAGAC	Bp 611-633 of *gfp* gene
TALEN-gfp target	forward	GATCTGGTGAACCGCATCGAGCTGAAGGGCATCGACTTCAAGGAGGACGGCAAGTG	Bp 362-407 of *gfp* gene
barley/tobacco	reverse	AATTCACTTGCCGTCCTCCTTGAAGTCGATGCCCTTCAGCTCGATGCGGTTCACCA	Bp 362-407 of *gfp* gene, complementary strand
TALEN-MLO target barley	forward	GATCGCTGGAACACGGCCTCCACAAGCTCGGCCATGTAAGTCCCGTTACCCTAGCTCAG	Bp 100-151 of *MLO* gene
	reverse	AATTCTGAGCTAGGGTAACGGGACTTACATGGCCGAGCTTGTGGAGGCCGTGTTCCAGC	Bp 100-151 of *MLO* gene, complementary strand
SfiI-A_f	forward	GTAGGCCCTTAAGGCCATG	Adapter sequence
SfiI-A_r	reverse	CATGGCCTTAAGGGCCTAC	Adapter sequence, complementary strand
SfiI-B_ f	forward	GTACGTAAGGCCGCCATGGCCAATG	Adapter sequence
SfiI-B_r	reverse	GTACCATTGGCCATGGCGGCCTTAC	Adapter sequence, complementary strand
GFP_PP1_f	forward	GGCGTCTTTGCTCAGGGCGGACT	Bp 611-633 of *gfp* gene
GFP_PP1_r	reverse	AAACAGTCCGCCCTGAGCAAAGA	Bp 611-633 of *gfp* gene, complementary strand
RGEN-gfp tobacco	forward	ATTGGCTGAAGGGCATCGACTTCA	Bp 375-394 of *gfp* gene
	reverse	AAACTGAAGTCGATGCCCTTCAGC	Bp 375-394 of *gfp* gene, complementary strand
Cas9 F2	forward	CAGCTCGTGCAGACCTACAAC	Bp 559-579 of *Cas9* gene
Cas9 R2	reverse	TGCCTTCTAAGGATAGCGTG	Bp 1258-1277 of *Cas9* gene, complementary strand
AtU6-26 F1	forward	CAGCTAGAGTCGAAGTAGTG	Bp 365-384 of *AtU6* promoter tobacco
35S P R1	reverse	GACAGATAGCTGGGCAATGG	Bp 133-152 of *CaMV 35S* promoter, complementary strand
35S P F1	forward	GACGTAAGGGATGACGCAC	Bp 350-368 of *CaMV 35S* promoter
BAR R2	reverse	GAGACGTACACGGTCGACTC	Bp 262-281 of *BAR* gene, complementary strand
GH-GFP R2	forward	TACGGCAAGCTGACCCTGAA	Bp 118-137 of *gfp* gene
GH-GFP F1	reverse	GGTCACGAACTCCAGCAGGA	Bp 659-678 of *gfp* gene, complementary strand
FokI F1	forward	ATCGAGATCGCCCGGAACAGCACC	Bp 13-36 of *FokI* gene
FokI R	reverse	ATCATCTCGCCGCCGATCAGGAGC	Bp 429-452 of *FokI* gene, complementary strand
GH-Ubi10 F	forward	CTATTGCTTCACCGCCTTAG	Bp 1010-1029 of *AtUBIQUITIN-10* promoter tobacco
TALEN R2	reverse	TGGCGGCTTGGCGCGTGACAG	Bp 32-52 of *gfp*-specific right TALEN unit<br>Bp 59-79 of gfp-specific left TALEN unit
GH-MLO-F3	forward	ACATCGGTCGTTTCTCACTC	Bp -396-206 of *MLO* gene
GH-MLO-R1	reverse	ACGCGCACGCCGATTACAAC	Bp -396-206 of *MLO* gene, complementary strand

Underlined are restriction enzyme recognition sites or respective overhangs and red font are the nucleotides to introduce frameshift in yfp gene of target vector
