# Supplementary material for: A simple test for the cleavage activity of customized endonucleases in plants
Source: Plant Methods. 2016 Mar 9;12:18. doi: 10.1186/s13007-016-0118-6 (PMC4784412; doi:10.1186/s13007-016-0118-6)
Supplement: Supplementary file 5 — 10.1186/s13007-016-0118-6 Details and sequences for the MLO-specific TALEN constructs. [file 13007_2016_118_MOESM5_ESM.pdf]

Additional file 5: Details and sequences for the *MLO*-specific TALEN constructs.

Name, description, source	Sequence
short *35S* promoter [1]	GGTCTCAGGAGGTCAACATGGTGGAGCACGACACTCTGGTCTACTCCAAAAATGTCAAAGATACAGTCTCAGAAGATCAAAGGGCTATTGAGACTTTTCAACAAAGGATAATTTCGGGAAACCTCCTCGGATTCCATTGCCCAGC<br>TATCTGTCACTTCATCGAAAGGACAGTAGAAAAGGAAGGTGGCTCCTACAAATGCCATCATTGCGATAAAGGAAAGGCTATCATTCAAGATCTCTCTGCCGACAGTGGTCCCAAAGATGGACCCCCACCCACGAGGAGCATCGTG<br>GAAAAAGAAGAGGTTCCAACCACGTCTACAAAGCAAGTGGATTGATGTGACATCTCCACTGACGTAAGGGATGACGCACAATCCCACTATCCTTCGCAAGACCCTTCCTCTATATAAGGAAGTTCATTTCATTTGGAGAGGACAC<br>GCTCAAGTATAAGAGCTCATTTTTACAACAATTACCAACAACAACAAACAACAAACAACATTACAATTACATTTACAATTATCGATACAATGTGAGACC
HA-NLS [2]	GGTCTCA**AATGGGCTACCCTTACGACGTGCCTGACTACGCCTCTAGACCCAAGAAAAAGCGGAAAGTGGGCATCCAC**GCTATGTGAGACC
truncated N-termius of *AvrBs3*	GGTCTCTT**ATGGATCTACGCACGCTCGGCTACAGCCAGCAGCAACAGGAGAAGATCAAACCGAAGGTTCGTTCGACAGTGGCGCAGCACCACGAGGCACTGGTCGGCCATGGGTTTACACACGCGCACATCGTTGCGCTCAG**<br>**CCAACACCCGGCAGCGTTAGGGACCGTCGCTGTCAAGTATCAGGACATGATCGCAGCGTTGCCAGAGGCGACACACGAAGCGATCGTTGGCGTCGGCAAACAGTGGTCCGGCGCACGCGCCCTGGAGGCCTTGCTCACGGT**<br>**GGCGGGAGAGTTGAGAGGTCCACCGTTACAGTTGGACACAGGCCAACTTCTCAAGATTGCAAAACGTGGCGGCGTGACCGCAGTGGAGGCAGTGCATGCATGGCGCAATGCACTGACGGGTGCCCCCCTGAACCTTACG**CG<br>GTCTTCCCATAATGAGACC
truncated C-terminus of *AvrBs3* analog to +28 [3]	GGTCTCTATAAGGGAAGACGGCGCTGGAG**AGCATTGTTGCCCAGTTATCTCGCCCTGATCCGGCGTTGGCCGCGTTGACCAACGACCACCTCGTCGCCTTGGCCTGCCTCGGCGGAAGCGGCGGAAGC**GGTGTGAGACC
FokI [4]	GGTCTCAGGTGGT**CAGCTAGTGAAATCTGAATTGGAAGAGAAGAAATCTGAACTTAGACATAAATTGAAATATGTGCCACATGAATATATTGAATTGATTGAAATCGCAAGAAATTCAACTCAGGATAGAATCCTTGAAATG**<br>**AAGGTGATGGAGTTCTTTATGAAGGTTTATGGTTATCGTGGTAAACATTTGGGTGGATCAAGGAAACCAGACGGAGCAATTTATACTGTCGGATCTCCTATTGATTACGGTGTGATCGTTGATACTAAGGCATATTCAGGAG**<br>**GTTATAATCTTCCAATTGGTCAAGCAGATGAAATGCAAAGATATGTCGAAGAGAATCAAACAAGAAACAAGCATATCAACCCTAATGAATGGTGGAAAGTCTATCCATCTTCAGTAACAGAATTTAAGTTCTTGTTTGTGAGT**<br>**GGTCATTTCAAAGGAAACTACAAAGCTCAGCTTACAAGATTGAATCATATCACTAATTGTAATGGAGCTGTTCTTAGTGTAGAAGAGCTTTTGATTGGTGGAGAAATGATTAAAGCTGGTACATTGACACTTGAGGAAGTGA**<br>**GAAGGAAATTTAATAACGGTGAGATAAACTTT**TAGGCTTTGAGACC
OCS terminator [1]	GTCTCAGCTTGTCCTGCTTTAATGAGATATGCGAGAAGCCTATGATCGCATGATATTTGCTTTCAATTCTGTTGTGCACGTTGTAAAAAACCTGAGCATGTGTAGCTCAGATCCTTACCGCCGGTTTCGGTTCATTCTAATGAATATA<br>TCACCCGTTACTATCGTATTTTTATGAATAATATTCTCCGTTCAATTTACTGATTGTACCCTACTACTTATATGTACAATATTAAAATGAAAACAATATATTGTGCTGAATAGGTTTATAGCGACATCTATGATAGAGCGCCACAATAA<br>CAAACAATTGCGTTTTATTATTACAAATCCAATTTTAAAAAAAGCGGCAGAACCGGTCAAACCTAAAAGACTGATTACATAAATCTTATTCAAATTTCAAAAGTGCCCCAGGGGCTAGTATCTACGACACACCGAGCGGCGAACTA<br>ATAACGCTCACTGAAGGGAACTCCGGTTCCCCGCCGGCGCGCATGGGTGAGATTCCTTGAAGTTGAGTATTGGCCGTCCGCTCTACCGAAAGTTACGGGCACCATTCAACCCGGTCCAGCACGGCGGCCGGGTAACCGACTTGCT<br>GCCCCGAGAATTATGCAGCATTTTTTTGGTGTATGTGGGCCCCAAATGAAGTGCAGGTCAAACCTTGACAGTGACGACAAATCGTTGGGCGGGTCCAGGGCGAATTTTGCGACAACATGTCGAGGCTCAGCAGGACCGCTTGAG<br>ACC
pUC57_BpiI/KpnI_shuttle (M13F to M13R)	TGTAAAACGACGGCCAGTGAATTCGAGCTCGGTACCTGCCTTGTCTTCTTAAAGCTAGCGTCAGTTTAAGAAGACTTGGGAGGTACCTCGCGAATGCATCTAGATATCGGATCCCGGGCCCGTCGACTGCAGAGGCCTGCATGCA<br>AGCTTGGCGTAATCATGGTCATAGCTGTTTCCTG
Kanamycin Resistance [4]	GAAGACAAGTAAAGCGGGAGGATCATGAGCGGAGAATTAAGGGAGTCACGTTATGACCCCCGCCGATGACGCGGGACAAGCCGTTTTACGTTTGGAACTGACAGAACCGCAACGTTGAAGGAGCCACTCAGCCGCGGGTTTCT<br>GGAGTTTAATGAGCTAAGCACATACGTCAGAAACCATTATTGCGCGTTCAAAAGTCGCCTAAGGTCACTATCAGCTAGCAAATATTTCTTGTCAAAAATGCTCCACTGACGTTCCATAAATTCCCCTCGGTATCCAATTAGAGTCTC<br>ATATTCACTCTCAATCCAAATAATCTGCACCGGATCTGGATCGTTTCGC**ATGATTGAACAAGATGGATTGCACGCAGGTTCTCCGGCCGCTTGGGTGGAGAGGCTATTCGGCTATGACTGGGCACAACAGACAATCGGCTGCTC**<br>**TGATGCCGCCGTGTTCCGGCTGTCAGCGCAGGGGCGCCCGGTTCTTTTTGTCAAGACCGACCTGTCCGGTGCCCTGAATGAACTGCAGGACGAGGCAGCGCGGCTATCGTGGCTGGCCACGACGGGCGTTCCTTGCGCAGCT**<br>**GTGCTCGACGTTGTCACTGAAGCGGGAAGGGACTGGCTGCTATTGGGCGAAGTGCCGGGGCAGGATCTCCTGTCATCTCACCTTGCTCCTGCCGAGAAAGTATCCATCATGGCTGATGCAATGCGGCGGCTGCATACGCTTG**<br>**ATCCGGCTACCTGCCCATTCGACCACCAAGCGAAACATCGCATCGAGCGAGCACGTACTCGGATGGAAGCCGGTCTTGTCGATCAGGATGATCTGGACGAAGAGCATCAGGGGCTCGCGCCAGCCGAACTGTTCGCCAGGCT**<br>**CAAGGCGCGCATGCCCGACGGCGATGATCTCGTCGTGACCCATGGCGATGCCTGCTTGCCGAATATCATGGTGGAAAATGGCCGCTTTTCTGGATTCATCGACTGTGGCCGGCTGGGTGTGGCGGACCGCTATCAGGACATA**<br>**GCGTTGGCTACCCGTGATATTGCTGAAGAGCTTGGCGGCGAATGGGCTGACCGCTTCCTCGTGCTTTACGGTATCGCCGCTCCCGATTCGCAGCGCATCGCCTTCTATCGCCTTCTTGACGAGTTCTTC**TGAGCGGGACTCTGGG<br>GTTCGAAATGACCGACCAAGCGACGCCCAACCTGCCATCACGAGATTTCGATTCCACCGCCGCCTTCTATGAAAGGTTGGGCTTCGGAATCGTTTTCCGGGACGCCGGCTGGATGATCCTCCAGCGCGGGGATCTCATGCTGGAG<br>TTCTTCGCCCACGGGATCTCTGCGGAACAGGCGGTCGAAGGTGCCGATATCATTACGACAGCAACGGCCGACAAGCACAACGCCACGATCCTGAGCGACAATATGATCGGGCCCGGCGTCCACATCAACGGCGTCGGCGGCGAC<br>TGCCCAGGCAAGACCGAGATGCACCGCGATATCTTGCTGCGTTCGGATATTTTCGTGGAGTTCCCGCCACAGACCCGGATGATCCCCGATCGTTCAAACATTTGGCAATAAAGTTTCTTAAGATTGAATCCTGTTGCCGGTCTTGC<br>GATGATTATCATATAATTTCTGTTGAATTACGTTAAGCATGTAATAATTAACATGTAATGCATGACGTTATTTATGAGATGGGTTTTTATGATTAGAGTCCCGCAATTATACATTTAATACGCGATAGAAAACAAAATATAGCGCGC<br>AAACTAGGATAAATTATCGCGCGCGGTGTCATCTATGTTACTAGACGCTCTCCGAGCTCGAATTCTAGTTTGTCTTC

References:

1. Weber E, Engler C, Gruetzner R, Werner S, Marillonnet S. A modular cloning system for standardized assembly of multigene constructs. PLoS ONE 2011;6:e16765.
2. de Lange O, Wolf C, Dietze J, Elsaesser J, Morbitzer R, Lahaye T. Programmable DNA-binding proteins from *Burkholderia* provide a fresh perspective on the TALE-like repeat domain. Nucleic Acids Res. 2014;42:7436-49.
3. Mussolino C, Morbitzer R, Lutge F, Dannemann N, Lahaye T, Cathomen T. A novel TALE nuclease scaffold enables high genome editing activity in combination with low toxicity. Nucleic Acids Res. 2011;39:9283-93.
4. Nakagawa T, Kurose T, Hino T, Tanaka K, Kawamukai M, Niwa Y, Toyooka K, Matsuoka K, Jinbo T, Kimura T. Development of series of gateway binary vectors, pGWBs, for realizing efficient construction of fusion genes for plant transformation. J Biosci Bioeng. 2007;104:34-41.
