# Supplementary material for: A simple test for the cleavage activity of customized endonucleases in plants
Source: Plant Methods. 2016 Mar 9;12:18. doi: 10.1186/s13007-016-0118-6 (PMC4784412; doi:10.1186/s13007-016-0118-6)
Supplement: Supplementary file 6 — 10.1186/s13007-016-0118-6 Plasmid maps and sequences of the constructs. [file 13007_2016_118_MOESM6_ESM.pdf]

gfp-specific RGEN vector used in tobacco pSI24

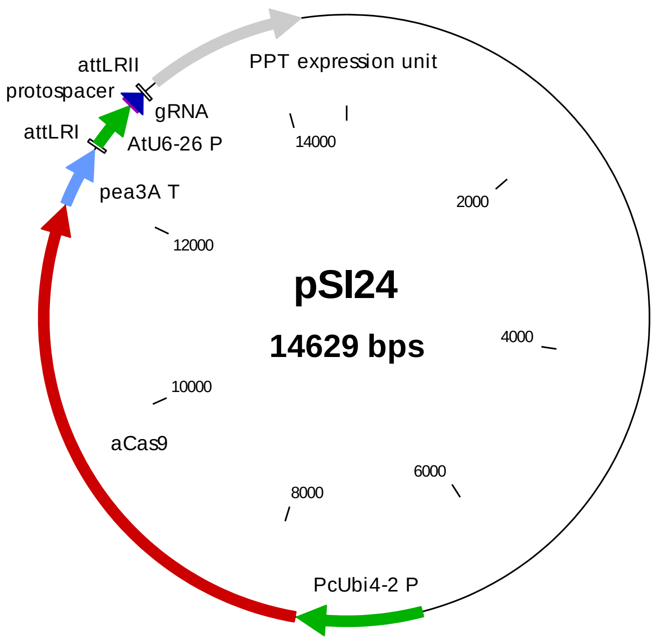


```
   1  AGTACTTTGA TCCAACCCCT CCGCTGCTAT AGTGCAGTCG GCTTCTGACG TTCAGTGCAG CCGTCTTCTG AAAACGACAT GTCGCACAAG TCCTAAGTTA
 101  CGCGACAGGC TGCCGCCCTG CCCTTTTCCT GGCGTTTTCT TGTCGCGTGT TTTAGTCGCA TAAAGTAGAA TACTTGCGAC TAGAACCGGA GACATTACGC
 201  CATGAACAAG AGCGCCGCCG CTGGCCTGCT GGGCTATGCC CGCGTCAGCA CCGACGACCA GGACTTGACC AACCAACGGG CCGAACTGCA CGCGGCCGGC
 301  TGCACCAAGC TGTTTTCCGA GAAGATCACC GGCACCAGGC GCGACCGCCC GGAGCTGGCC AGGATGCTTG ACCACCTACG CCCTGGCGAC GTTGTGACAG
 401  TGACCAGGCT AGACCGCCTG GCCCGCAGCA CCCGCGACCT ACTGGACATT GCCGAGCGCA TCCAGGAGGC CGGCGCGGGC CTGCGTAGCC TGGCAGAGCC
 501  GTGGGCCGAC ACCACCACGC CGGCCGGCCG CATGGTGTTG ACCGTGTTCG CCGGCATTGC CGAGTTCGAG CGTTCCCTAA TCATCGACCG CACCCGGAGC
 601  GGGCGCGAGG CCGCCAAGGC CCGAGGCGTG AAGTTTGGCC CCCGCCCTAC CCTCACCCCG GCACAGATCG CGCACGCCCG CGAGCTGATC GACCAGGAAG
 701  GCCGCACCGT GAAAGAGGCG GCTGCACTGC TTGGCGTGCA TCGCTCGACC CTGTACCGCG CACTTGAGCG CAGCGAGGAA GTGACGCCCA CCGAGGCCAG
 801  GCGGCGCGGT GCCTTCCGTG AGGACGCATT GACCGAGGCC GACGCCCTGG CGGCCGCCGA GAATGAACGC CAAGAGGAAC AAGCATGAAA CCGCACCAGG
 901  ACGGCCAGGA CGAACCGTTT TTCATTACCG AAGAGATCGA GGCGGAGATG ATCGCGGCCG GGTACGTGTT CGAGCCGCCC GCGCACGTCT CAACCGTGCG
1001  GCTGCATGAA ATCCTGGCCG GTTTGTCTGA TGCCAAGCTG GCGGCCTGGC CGGCCAGCTT GGCCGCTGAA GAAACCGAGC GCCGCCGTCT AAAAAGGTGA
1101  TGTGTATTTG AGTAAAACAG CTTGCGTCAT GCGGTCGCTG CGTATATGAT GCGATGAGTA AATAAACAAA TACGCAAGGG GAACGCATGA AGGTTATCGC
1201  TGTACTTAAC CAGAAAGGCG GGTCAGGCAA GACGACCATC GCAACCCATC TAGCCCGCGC CCTGCAACTC GCCGGGGCCG ATGTTCTGTT AGTCGATTCC
1301  GATCCCCAGG GCAGTGCCCG CGATTGGGCG GCCGTGCGGG AAGATCAACC GCTAACCGTT GTCGGCATCG ACCGCCCGAC GATTGACCGC GACGTGAAGG
1401  CCATCGGCCG GCGCGACTTC GTAGTGATCG ACGGAGCGCC CCAGGCGGCG GACTTGGCTG TGTCCGCGAT CAAGGCAGCC GACTTCGTGC TGATTCCGGT
1501  GCAGCCAAGC CCTTACGACA TATGGGCCAC CGCCGACCTG GTGGAGCTGG TTAAGCAGCG CATTGAGGTC ACGGATGGAA GGCTACAAGC GGCCTTTGTC
1601  GTGTCGCGGG CGATCAAAGG CACGCGCATC GGCGGTGAGG TTGCCGAGGC GCTGGCCGGG TACGAGCTGC CCATTCTTGA GTCCCGTATC ACGCAGCGCG
1701  TGAGCTACCC AGGCACTGCC GCCGCCGGCA CAACCGTTCT TGAATCAGAA CCCGAGGGCG ACGCTGCCCG CGAGGTCCAG GCGCTGGCCG CTGAAATTAA
1801  ATCAAAACTC ATTTGAGTTA ATGAGGTAAA GAGAAAATGA GCAAAAGCAC AAACACGCTA AGTGCCGGCC GTCCGAGCGC ACGCAGCAGC AAGGCTGCAA
1901  CGTTGGCCAG CCTGGCAGAC ACGCCAGCCA TGAAGCGGGT CAACTTTCAG TTGCCGGCGG AGGATCACAC CAAGCTGAAG ATGTACGCGG TACGCCAAGG
2001  CAAGACCATT ACCGAGCTGC TATCTGAATA CATCGCGCAG CTACCAGAGT AAATGAGCAA ATGAATAAAT GAGTAGATGA ATTTTAGCGG CTAAAGGAGG
2101  CGGCATGGAA AATCAAGAAC AACCAGGCAC CGACGCCGTG GAATGCCCCA TGTGTGGAGG AACGGGCGGT TGGCCAGGCG TAAGCGGCTG GGTTGTCTGC
2201  CGGCCCTGCA ATGGCACTGG AACCCCCAAG CCCGAGGAAT CGGCGTGACG GTCGCAAACC ATCCGGCCCG GTACAAATCG GCGCGGCGCT GGGTGATGAC
2301  CTGGTGGAGA AGTTGAAGGC CGCGCAGGCC GCCCAGCGGC AACGCATCGA GGCAGAAGCA CGCCCCGGTG AATCGTGGCA AGCGGCCGCT GATCGAATCC
2401  GCAAAGAATC CCGGCAACCG CCGGCAGCCG GTGCGCCGTC GATTAGGAAG CCGCCCAAGG GCGACGAGCA ACCAGATTTT TTCGTTCCGA TGCTCTATGA
2501  CGTGGGCACC CGCGATAGTC GCAGCATCAT GGACGTGGCC GTTTTCCGTC TGTCGAAGCG TGACCGACGA GCTGGCGAGG TGATCCGCTA CGAGCTTCCA
2601  GACGGGCACG TAGAGGTTTC CGCAGGGCCG GCCGGCATGG CCAGTGTGTG GGATTACGAC CTGGTACTGA TGGCGGTTTC CCATCTAACC GAATCCATGA
2701  ACCGATACCG GGAAGGGAAG GGAGACAAGC CCGGCCGCGT GTTCCGTCCA CACGTTGCGG ACGTACTCAA GTTCTGCCGG CGAGCCGATG GCGGAAAGCA
2801  GAAAGACGAC CTGGTAGAAA CCTGCATTCG GTTAAACACC ACGCACGTTG CCATGCAGCG TACGAAGAAG GCCAAGAACG GCCGCCTGGT GACGGTATCC
2901  GAGGGTGAAG CCTTGATTAG CCGCTACAAG ATCGTAAAGA GCGAAACCGG GCGGCCGGAG TACATCGAGA TCGAGCTAGC TGATTGGATG TACCGCGAGA
3001  TCACAGAAGG CAAGAACCCG GACGTGCTGA CGGTTCACCC CGATTACTTT TTGATCGATC CCGGCATCGG CCGTTTTCTC TACCGCCTGG CACGCCGCGC
3101  CGCAGGCAAG GCAGAAGCCA GATGGTTGTT CAAGACGATC TACGAACGCA GTGGCAGCGC CGGAGAGTTC AAGAAGTTCT GTTTCACCGT GCGCAAGCTG
3201  ATCGGGTCAA ATGACCTGCC GGAGTACGAT TTGAAGGAGG AGGCGGGGCA GGCTGGCCCG ATCCTAGTCA TGCGCTACCG CAACCTGATC GAGGGCGAAG
3301  CATCCGCCGG TTCCTAATGT ACGGAGCAGA TGCTAGGGCA AATTGCCCTA GCAGGGGAAA AAGGTCGAAA AGGTCTCTTT CCTGTGGATA GCACGTACAT
3401  TGGGAACCCA AAGCCGTACA TTGGGAACCG GAACCCGTAC ATTGGGAACC CAAAGCCGTA CATTGGGAAC CGGTCACACA TGTAAGTGAC TGATATAAAA
3501  GAGAAAAAAG GCGATTTTTC CGCCTAAAAC TCTTTAAAAC TTATTAAAAC TCTTAAAACC CGCCTGGCCT GTGCATAACT GTCTGGCCAG CGCACAGCCG
3601  AAGAGCTGCA AAAAGCGCCT ACCCTTCGGT CGCTGCGCTC CCTACGCCCC GCCGCTTCGC GTCGGCCTAT CGCGGCCGCT GGCCGCTCAA AAATGGCTGG
3701  CCTACGGCCA GGCAATCTAC CAGGGCGCGG ACAAGCCGCG CCGTCGCCAC TCGACCGCCG GCGCCCACAT CAAGGCACCC TGCCTCGCGC GTTTCGGTGA
3801  TGACGGTGAA AACCTCTGAC ACATGCAGCT CCCGGAGACG GTCACAGCTT GTCTGTAAGC GGATGCCGGG AGCAGACAAG CCCGTCAGGG CGCGTCAGCG
3901  GGTGTTGGCG GGTGTCGGGG CGCAGCCATG ACCCAGTCAC GTAGCGATAG CGGAGTGTAT ACTGGCTTAA CTATGCGGCA TCAGAGCAGA TTGTACTGAG
4001  AGTGCACCAT ATGCGGTGTG AAATACCGCA CAGATGCGTA AGGAGAAAAT ACCGCATCAG GCGCTCTTCC GCTTCCTCGC TCACTGACTC GCTGCGCTCG
4101  GTCGTTCGGC TGCGGCGAGC GGTATCAGCT CACTCAAAGG CGGTAATACG GTTATCCACA GAATCAGGGG ATAACGCAGG AAAGAACATG TGAGCAAAAG
```

4201 GCCAGCAAAA GGCCAGGAAC CGTAAAAAGG CCGCGTTGCT GGCGTTTTTC CATAGGCTCC GCCCCCCTGA CGAGCATCAC AAAAATCGAC GCTCAAGTCA
4301 GAGGTGGCGA AACCCGACAG GACTATAAAG ATACCAGGCG TTTCCCCCTG GAAGCTCCCT CGTGCGCTCT CCTGTTCCGA CCCTGCCGCT TACCGGATAC
4401 CTGTCCGCCT TTCTCCCTTC GGGAAGCGTG GCGCTTTCTC ATAGCTCACG CTGTAGGTAT CTCAGTTCGG TGTAGGTCGT TCGCTCCAAG CTGGGCTGTG
4501 TGCACGAACC CCCCGTTCAG CCCGACCGCT GCGCCTTATC CGGTAACTAT CGTCTTGAGT CCAACCCGGT AAGACACGAC TTATCGCCAC TGGCAGCAGC
4601 CACTGGTAAC AGGATTAGCA GAGCGAGGTA TGTAGGCGGT GCTACAGAGT TCTTGAAGTG GTGGCCTAAC TACGGCTACA CTAGAAGGAC AGTATTTGGT
4701 ATCTGCGCTC TGCTGAAGCC AGTTACCTTC GGAAAAAGAG TTGGTAGCTC TTGATCCGGC AAACAAACCA CCGCTGGTAG CGGTGGTTTT TTTGTTTGCA
4801 AGCAGCAGAT TACGCGCAGA AAAAAAGGAT CTCAAGAAGA TCCTTTGATC TTTTCTACGG GGTCTGACGC TCAGTGGAAC GAAAACTCAC GTTAAGGGAT
4901 TTTGGTCATG CATGATATAT CTCCCAATTT GTGTAGGGCT TATTATGCAC GCTTAAAAAT AATAAAAGCA GACTTGACCT GATAGTTTGG CTGTGAGCAA
5001 TTATGTGCTT AGTGCATCTA ATCGCTTGAG TTAACGCCGG CGAAGCGGCG TCGGCTTGAA CGAATTTCTA GCTAGACATT ATTTGCCGAC TACCTTGGTG
5101 ATCTCGCCTT TCACGTAGTG GACAAATTCT TCCAACTGAT CTGCGCGCGA GGCCAAGCGA TCTTCTTCTT GTCCAAGATA AGCCTGTCTA GCTTCAAGTA
5201 TGACGGGCTG ATACTGGGCC GGCAGGCGCT CCATTGCCCA GTCGGCAGCG ACATCCTTCG GCGCGATTTT GCCGGTTACT GCGCTGTACC AAATGCGGGA
5301 CAACGTAAGC ACTACATTTC GCTCATCGCC AGCCCAGTCG GGCGGCGAGT TCCATAGCGT TAAGGTTTCA TTTAGCGCCT CAAATAGATC CTGTTCAGGA
5401 ACCGGATCAA AGAGTTCCTC CGCCGCTGGA CCTACCAAGG CAACGCTATG TTCTCTTGCT TTTGTCAGCA AGATAGCCAG ATCAATGTCG ATCGTGGCTG
5501 GCTCGAAGAT ACCTGCAAGA ATGTCATTGC GCTGCCATTC TCCAAATTGC AGTTCGCGCT TAGCTGGATA ACGCCACGGA ATGATGTCGT CGTGCACAAC
5601 AATGGTGACT TCTACAGCGC GGAGAATCTC GCTCTCTCCA GGGGAAGCCG AAGTTTCCAA AAGGTCGTTG ATCAAAGCTC GCCGCGTTGT TTCATCAAGC
5701 CTTACGGTCA CCGTAACCAG CAAATCAATA TCACTGTGTG GCTTCAGGCC GCCATCCACT GCGGAGCCGT ACAAATGTAC GGCCAGCAAC GTCGGTTCGA
5801 GATGGCGCTC GATGACGCCA ACTACCTCTG ATAGTTGAGT CGATACTTCG GCGATCACCG CTTCCCCCAT GATGTTTAAC TTTGTTTTAG GGCGACTGCC
5901 CTGCTGCGTA ACATCGTTGC TGCTCCATAA CATCAAACAT CGACCCACGG CGTAACGCGC TTGCTGCTTG GATGCCCGAG GCATAGACTG TACCCCAAAA
6001 AAACATGTCA TAACAAGAAG CCATGAAAAC CGCCACTGCG CCGTTACCAC CGCTGCGTTC GGTCAAGGTT CTGGACCAGT TGCGTGACGG CAGTTACGCT
6101 ACTTGCATTA CAGCTTACGA ACCGAACGAG GCTTATGTCC ACTGGGTTCG TGCCCGAATT GATCACAGGC AGCAACGCTC TGTCATCGTT ACAATCAACA
6201 TGCTACCCTC CGCGAGATCA TCCGTGTTTC AAACCCGGCA GCTTAGTTGC CGTTCTTCCG AATAGCATCG GTAACATGAG CAAAGTCTGC CGCCTTACAA
6301 CGGCTCTCCC GCTGACGCCG TCCCGGACTG ATGGGCTGCC TGTATCGAGT GGTGATTTTG TGCCGAGCTG CCGGTCGGGG AGCTGTTGGC TGGCTGGTGG
6401 CAGGATATAT TGTGGTGTAA ACAAATTGAC GCTTAGACAA CTTAATAACA CATTGCGGAC GTTTTTAATG TACTGAATTA ACGCCGAATT GCTCTAGCCA
6501 ATACGCAAAC CGCCTCTCCC CGCGCGTTGG CCGATTCATT AATGCAGCTG GCACGACAGG TTTCCCGACT GGAAAGCGGG CAGTGAGCGC AACGCAATTA
6601 ATGTGAGTTA GCTCACTCAT TAGGCACCCC AGGCTTTACA CTTTATGCTT CCGGCTCGTA TGTTGTGTGG AATTGTGAGC GGATAACAAT TTCACACAGG
6701 AAACAGCTAT GACATGATTA CGAATTCAAA AATTACGGAT ATGAATATAG GCATATCCGT ATCCGAATTA TCCGTTTGAC AGCTAGCAAC GATTGTACAA
6801 TTGCTTCTTT AAAAAAGGAA GAAAGAAAGA AAGAAAAGAA TCAACATCAG CGTTAACAAA CGGCCCCGTT ACGGCCCAAA CGGTCATATA GAGTAACGGC
6901 GTTAAGCGTT GAAAGACTCC TATCGAAATA CGTAACCGCA AACGTGTCAT AGTCAGATCC CCTCTTCCTT CACCGCCTCA AACACAAAAA TAATCTTCTA
7001 CAGCCTATAT ATACAACCCC CCCTTCTATC TCTCCTTTCT CACAATTCAT CATCTTTCTT TCTCTACCCC CAATTTTAAG AAATCCTCTC TTCTCCTCTT
7101 CATTTTCAAG GTAAATCTCT CTCTCTCTCT CTCTCTCTGT TATTCCTTGT TTTAATTAGG TATGTATTAT TGCTAGTTTG TTAATCTGCT TATCTTATGT
7201 ATGCCTTATG TGAATATCTT TATCTTGTTC ATCTCATCCG TTTAGAAGCT ATAAATTTGT TGATTTGACT GTGTATCTAC ACGTGGTTAT GTTTATATCT
7301 AATCAGATAT GAATTTCTTC ATATTGTTGC GTTTGTGTGT ACCAATCCGA AATCGTTGAT TTTTTTCATT TAATCGTGTA GCTAATTGTA CGTATACATA
7401 TGGATCTACG TATCAATTGT TCATCTGTTT GTGTTTGTAT GTATACAGAT CTGAAAACAT CACTTCTCTC ATCTGATTGT GTTGTTACAT ACATAGATAT
7501 AGATCTGTTA TATCATTTTT TTTATTAATT GTGTATATAT ATATGTGCAT AGATCTGGAT TACATGATTG TGATTATTTA CATGATTTTG TTATTTACGT
7601 ATGTATATAT GTAGATCTGG ACTTTTTGGA GTTGTTGACT TGATTGTATT TGTGTGTGTA TATGTGTGTT CTGATCTTGA TATGTTATGT ATGTGCAGCG
7701 AATTCGGCGC GCCATGGATA AGAAGTACTC TATCGGACTC GATATCGGAA CTAACTCTGT GGGATGGGCT GTGATCACCG ATGAGTACAA GGTGCCATCT
7801 AAGAAGTTCA AGGTTCTCGG AAACACCGAT AGGCACTCTA TCAAGAAAAA CCTTATCGGT GCTCTCCTCT TCGATTCTGG TGAAACTGCT GAGGCTACCA
7901 GACTCAAGAG AACCGCTAGA AGAAGGTACA CCAGAAGAAA GAACAGGATC TGCTACCTCC AAGAGATCTT CTCTAACGAG ATGGCTAAAG TGGATGATTC
8001 ATTCTTCCAC AGGCTCGAAG AGTCATTCCT CGTGGAAGAA GATAAGAAGC ACGAGAGGCA CCCTATCTTC GGAAACATCG TTGATGAGGT GGCATACCAC
8101 GAGAAGTACC CTACTATCTA CCACCTCAGA AAGAAGCTCG TTGATTCTAC TGATAAGGCT GATCTCAGGC TCATCTACCT CGCTCTCGCT CACATGATCA
8201 AGTTCAGAGG ACACTTCCTC ATCGAGGGTG ATCTCAACCC TGATAACTCT GATGTGGATA AGTTGTTCAT CCAGCTCGTG CAGACCTACA ACCAGCTTTT
8301 CGAAGAGAAC CCTATCAACG CTTCAGGTGT GGATGCTAAG GCTATCCTCT CTGCTAGGCT CTCTAAGTCA AGAAGGCTTG AGAACCTCAT TGCTCAGCTC
8401 CCTGGTGAGA AGAAGAACGG ACTTTTCGGA AACTTGATCG CTCTCTCTCT CGGACTCACC CCTAACTTCA AGTCTAACTT CGATCTCGCT GAGGATGCAA
8501 AGCTCCAGCT CTCAAAGGAT ACCTACGATG ATGATCTCGA TAACCTCCTC GCTCAGATCG GAGATCAGTA CGCTGATTTG TTCCTCGCTG CTAAGAACCT
8601 CTCTGATGCT ATCCTCCTCA GTGATATCCT CAGAGTGAAC ACCGAGATCA CCAAGGCTCC ACTCTCAGCT TCTATGATCA AGAGATACGA TGAGCACCAC
8701 CAGGATCTCA CACTTCTCAA GGCTCTTGTT AGACAGCAGC TCCCAGAGAA GTACAAAGAG ATTTTCTTCG ATCAGTCTAA GAACGGATAC GCTGGTTACA
8801 TCGATGGTGG TGCATCTCAA GAAGAGTTCT ACAAGTTCAT CAAGCCTATC CTCGAGAAGA TGGATGGAAC CGAGGAACTC CTCGTGAAGC TCAATAGAGA
8901 GGATCTTCTC AGAAAGCAGA GGACCTTCGA TAACGGATCT ATCCCTCATC AGATCCACCT CGGAGAGTTG CACGCTATCC TTAGAAGGCA AGAGGATTTC
9001 TACCCATTCC TCAAGGATAA CAGGGAAAAG ATTGAGAAGA TTCTCACCTT CAGAATCCCT TACTACGTGG GACCTCTCGC TAGAGGAAAC TCAAGATTCG
9101 CTTGGATGAC CAGAAAGTCT GAGGAAACCA TCACCCCTTG GAACTTCGAA GAGGTGGTGG ATAAGGGTGC TAGTGCTCAG TCTTTCATCG AGAGGATGAC
9201 CAACTTCGAT AAGAACCTTC CAAACGAGAA GGTGCTCCCT AAGCACTCTT TGCTCTACGA GTACTTCACC GTGTACAACG AGTTGACCAA GGTTAAGTAC
9301 GTGACCGAGG GAATGAGGAA GCCTGCTTTT TTGTCAGGTG AGCAAAAGAA GGCTATCGTT GATCTCTTGT TCAAGACCAA CAGAAAGGTG ACCGTGAAGC
9401 AGCTCAAAGA GGATTACTTC AAGAAAATCG AGTGCTTCGA TTCAGTTGAG ATTTCTGGTG TTGAGGATAG GTTCAACGCA TCTCTCGGAA CCTACCACGA
9501 TCTCCTCAAG ATCATTAAGG ATAAGGATTT CTTGGATAAC GAGGAAAACG AGGATATCTT GGAGGATATC GTTCTTACCC TCACCCTCTT TGAAGATAGA
9601 GAGATGATTG AAGAAAGGCT CAAGACCTAC GCTCATCTCT TCGATGATAA GGTGATGAAG CAGTTGAAGA GAAGAAGATA CACTGGTTGG GGAAGGCTCT
9701 CAAGAAAGCT CATTAACGGA ATCAGGGATA AGCAGTCTGG AAAGACAATC CTTGATTTCC TCAAGTCTGA TGGATTCGCT AACAGAAACT TCATGCAGCT
9801 CATCCACGAT GATTCTCTCA CCTTTAAAGA GGATATCCAG AAGGCTCAGG TTTCAGGACA GGGTGATAGT CTCCATGAGC ATATCGCTAA CCTCGCTGGA
9901 TCTCCTGCAA TCAAGAAGGG AATCCTCCAG ACTGTGAAGG TTGTGGATGA GTTGGTGAAG GTGATGGGAA GGCATAAGCC TGAGAACATC GTGATCGAAA
10001 TGGCTAGAGA GAACCAGACC ACTCAGAAGG GACAGAAGAA CTCTAGGGAA AGGATGAAGA GGATCGAGGA AGGTATCAAA GAGCTTGGAT CTCAGATCCT
10101 CAAAGAGCAC CCTGTTGAGA ACACTCAGCT CCAGAATGAG AAGCTCTACC TCTACTACCT CCAGAACGGA AGGGATATGT ATGTGGATCA AGAGTTGGAT
10201 ATCAACAGGC TCTCTGATTA CGATGTTGAT CATATCGTGC CACAGTCATT CTTGAAGGAT GATTCTATCG ATAACAAGGT GCTCACCAGG TCTGATAAGA
10301 ACAGGGGTAA GAGTGATAAC GTGCCAAGTG AAGAGGTTGT GAAGAAAATG AAGAACTATT GGAGGCAGCT CCTCAACGCT AAGCTCATCA CTCAGAGAAA
10401 GTTCGATAAC TTGACTAAGG CTGAGAGGGG AGGACTCTCT GAATTGGATA AGGCAGGATT CATCAAGAGG CAGCTTGTGG AAACCAGGCA GATCACTAAG
10501 CACGTTGCAC AGATCCTCGA TTCTAGGATG AACACCAAGT ACGATGAGAA CGATAAGTTG ATCAGGGAAG TGAAGGTTAT CACCCTCAAG TCAAAGCTCG
10601 TGTCTGATTT CAGAAAGGAT TTCCAATTCT ACAAGGTGAG GGAAATCAAC AACTACCACC ACGCTCACGA TGCTTACCTT AACGCTGTTG TTGGAACCGC
10701 TCTCATCAAG AAGTATCCTA AGCTCGAGTC AGAGTTCGTG TACGGTGATT ACAAGGTGTA CGATGTGAGG AAGATGATCG CTAAGTCTGA GCAAGAGATC
10801 GGAAAGGCTA CCGCTAAGTA TTTCTTCTAC TCTAACATCA TGAATTTCTT CAAGACCGAG ATTACCCTCG CTAACGGTGA GATCAGAAAG AGGCCACTCA
10901 TCGAGACAAA CGGTGAAACA GGTGAGATCG TGTGGGATAA GGGAAGGGAT TTCGCTACCG TTAGAAAGGT GCTCTCTATG CCACAGGTGA ACATCGTTAA
11001 GAAAACCGAG GTGCAGACCG GTGGATTCTC TAAAGAGTCT ATCCTCCCTA AGAGGAACTC TGATAAGCTC ATTGCTAGGA AGAAGGATTG GGACCCTAAG
11101 AAATACGGTG GTTTCGATTC TCCTACCGTG GCTTACTCTG TTCTCGTTGT GGCTAAGGTT GAGAAGGGAA AGAGTAAGAA GCTCAAGTCT GTTAAGGAAC
11201 TTCTCGGAAT CACTATCATG GAAAGGTCAT CTTTCGAGAA GAACCCAATC GATTTCCTCG AGGCTAAGGG ATACAAAGAG GTTAAGAAGG ATCTCATCAT
11301 CAAGCTCCCA AAGTACTCAC TCTTCGAACT CGAGAACGGT AGAAAGAGGA TGCTCGCTTC TGCTGGTGAG CTTCAAAAGG GAAACGAGCT TGCTCTCCCA
11401 TCTAAGTACG TTAACTTTCT TTACCTCGCT TCTCACTACG AGAAGTTGAA GGGATCTCCA GAAGATAACG AGCAGAAGCA ACTTTTCGTT GAGCAGCACA
11501 AGCACTACTT GGATGAGATC ATCGAGCAGA TCTCTGAGTT CTCTAAAAGG GTGATCCTCG CTGATGCAAA CCTCGATAAG GTGTTGTCTG CTTACAACAA
11601 GCACAGAGAT AAGCCTATCA GGGAACAGGC AGAGAACATC ATCCATCTCT TCACCCTTAC CAACCTCGGT GCTCCTGCTG CTTTCAAGTA CTTCGATACA
11701 ACCATCGATA GGAAGAGATA CACCTCTACC AAAGAAGTGC TCGATGCTAC CCTCATCCAT CAGTCTATCA CTGGACTCTA CGAGACTAGG ATCGATCTCT
11801 CACAGCTCGG TGGTGATTCA AGGGCTGATC CTAAGAAGAA GAGGAAGGTT TGAGGCGCGC CGAGCTCCAG GCCTCCCAGC TTTCGTCCGT ATCATCGGTT
11901 TCGACAACGT TCGTCAAGTT CAATGCATCA GTTTCATTGC CCACACACCA GAATCCTACT AAGTTTGAGT ATTATGGCAT TGGAAAAGCT GTTTTCTTCT
12001 ATCATTTGTT CTGCTTGTAA TTTACTGTGT TCTTTCAGTT TTTGTTTTCG GACATCAAAA TGCAAATGGA TGGATAAGAG TTAATAAATG ATATGGTCCT
12101 TTTGTTCATT CTCAAATTAT TATTATCTGT TGTTTTTACT TTAATGGGTT GAATTTAAGT AAGAAAGGAA CTAACAGTGT GATATTAAGG TGCAATGTTA
12201 GACATATAAA ACAGTCTTTC ACCTCTCTTT GGTTATGTCT TGAATTGGTT TGTTTCTTCA CTTATCTGTG TAATCAAGTT TACTATGAGT CTATGATCAA
12301 GTAATTATGC AATCAAGTTA AGTACAGTAT AGGCTTGAGC TCCCTAGGCC TGTTATCCCT AACAAGTTTG TACAAAAAAG CAGGCTCTTT TTTTCTTCTT
12401 CTTCGTTCAT ACAGTTTTTT TTTGTTTATC AGCTTACATT TTCTTGAACC GTAGCTTTCG TTTTCTTCTT TTTAACTTTC CATTCGGAGT TTTTGTATCT
12501 TGTTTCATAG TTTGTCCCAG GATTAGAATG ATTAGGCATC GAACCTTCAA GAATTTGATT GAATAAAACA TCTTCATTCT TAAGATATGA AGATAATCTT
12601 CAAAAGGCCC CTGGGAATCT GAAAGAAGAG AAGCAGGCCC ATTTATATGG GAAAGAACAA TAGTATTTCT TATATAGGCC CATTTAAGTT GAAAACAATC
12701 TTCAAAAGTC CCACATCGCT TAGATAAGAA AACGAAGCTG AGTTTATATA CAGCTAGAGT CGAAGTAGTG ATTGGCTGAA GGGCATCGAC TTCAGTTTTA
12801 GAGCTAGAAA TAGCAAGTTA AAATAAGGCT AGTCCGTTAT CAACTTGAAA AAGTGGCACC GAGTCGGTGC TTTTTTTCTA GACCCAGCTT TCTTGTACAA
12901 AGTTGGCATT AACCCAGCTT TCTTGTACAA AGTGGTTCGA TAATTCCGAT CCAGCCTAGG CCCGGGCCTG AGGACGCGTC CATGGTTAAT TAAGACGTCC
13001 GGACCGACTA GTGGATCCTC TAGAGTCGAC CTGCAGGCAT GCAAGCTTCT TCGTCAACAT GGTGGAGCAC GACACGCTTG TCTACTCCAA AAATATCAAA
13101 GATACAGTCT CAGAAGACCA AAGGGCAATT GAGACTTTTC AACAAAGGGT AATATCCGGA AACCTCCTCG GATTCCATTG CCCAGCTATC TGTCACTTTA
13201 TTGTGAAGAT AGTGGAAAAG GAAGGTGGCT CCTACAAATG CCATCATTGC GATAAAGGAA AGGCCATCGT TGAAGATGCC TCTGCCGACA GTGGTCCCAA
13301 AGATGGACCC CCACCCACGA GGAGCATCGT GGAAAAAGAA GACGTTCCAA CCACGTCTTC AAAGCAAGTG GATTGATGTG ATATCTCCAC TGACGTAAGG
13401 GATGACGCAC AATCAATCCC ACTATCCTTC GCAAGACCCT TTTAAGGGGG AAGTTCATTT CATTTGGAGA GGACACGCTG AAATCACCAG TCTCTCTGTA
13501 CAAATCNATC TCTCTCTATA ATATTGTGTA AGTAGTTCCC AGATAAGGGA ATTAGGGTTC TTATAGGGTT TCGCTCAGCT GTTGAGCATA TAAGAAACCC
13601 TTAGTCGATA GATCTGTTGG GGATCTACCA TGAGCCCAGA ACGACGCCCG GCCGACATCC GCCGTGCCAC CGAGGCGGAC ATGCCGGCGG TCTGCACCAT
13701 CGTCAACCAC TACATCGAGA CAAGCACGGT CAACTTCCGT ACCGAGCCGC AGGAACCGCA GGAGTGGACG GACGACCTCG TCCGTCTGCG GGAGCGCTAT
13801 CCCTGGCTCG TCGCCGAGGT GGACGGCGAG GTCGCCGGCA TCGCCTACGC GGGCCCCTGG AAGGCACGCA ACGCCTACGA CTGGACGGCC GAGTCGACCG
13901 TGTACGTCTC CCCCCGCCAC CAGCGGACGG GACTGGGCTC CACGCTCTAC ACCCACCTGC TGAAGTCCCT GGAGGCACAG GGCTTCAAGA GCGTGGTCGC
14001 TGTCATCGGG CTGCCCAACG ACCCGAGCGT GCGCATGCAC GAGGCGCTCG GATATGCCCC CCGCGGCATG CTGCGGGCGG CCGGCTTCAA GCACGGGAAC
14101 TGGCATGACG TGGGTTTCTG GCAGCTGGAC TTCAGCCTGC CGGTACCGCC CCGTCCGGTC CTGCCCGTCA CCGAGATCTG ATGACCCAAC TTAGTATGTA
14201 TTTGTATTTG TAAAATACTT CTATCAATAA AATTTCTAAT TCCTAAAACC AAAATCCAGG GGTACCGAAC AAGCTTGGCA CTGGCCGTCG TTTTACAACG
14301 TCGTGACTGG GAAAACCCTG GCGTTACCCA ACTTAATCGC CTTGCAGCAC ATCCCCCTTT CGCCAGCTGG CGTAATAGCG AAGAGGCCCG CACCGATCGC
14401 CCTTCCCAAC AGTTGCGCAG CCTGAATGGC GAATGAGCTT GAGCTTGGAT CAGATTGTCG TTTCCCGCCT TCAGTTTAAA CTATCAGTGT TTGACAGGAT
14501 ATATTGGCGG GTAAACCTAA GAGAAAAGAG CGTTTATTAG AATAACGGAT ATTTAAAAGG GCGTGAAAAG GTTTATCCGT TCGTCCATTT GTATGTGCAT
14601 GCCAACCACA GGGTTCCCCT CGGGATCAA

gfp-specific left TALEN vector used in tobacco pSP10

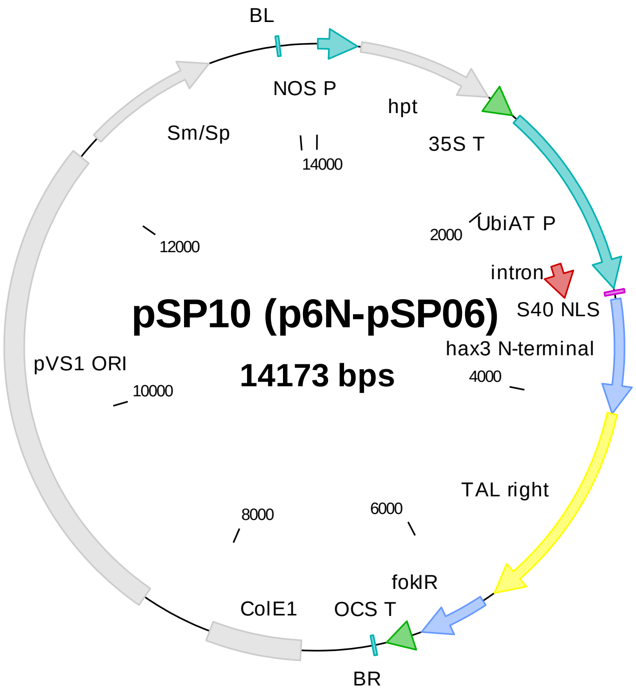


```
   1 TCTAGATCAT GAGCGGAGAA TTAAGGGAGT CACGTTATGA CCCCCGCCGA TGACGCGGGA CAAGCCGTTT TACGTTTGGA ACTGACAGAA CCGCAACGTT
 101 GAAGGAGCCA CTCAGCCGCG GGTTTCTGGA GTTTAATGAG CTAAGCACAT ACGTCAGAAA CCATTATTGC GCGTTCAAAA GTCGCCTAAG GTCACTATCA
 201 GCTAGCAAAT ATTTCTTGTC AAAAATGCTC CACTGACGTT CCATAAATTC CCCTCGGTAT CCAATTAGAG TCTCATATTC ACTCTCAATC CAAATAATCT
 301 GCACCTCGAG GGCCCCGGGG GGCAATAAGA TATGAAAAAG CCTGAACTCA CCGCGACGTC TGTCGAGAAG TTTCTGATCG AAAAGTTCGA CAGCGTCTCC
 401 GACCTGATGC AGCTCTCGGA GGGCGAAGAA TCTCGTGCTT TCAGCTTCGA TGTAGGAGGG CGTGGATATG TCCTGCGGGT AAATAGCTGC GCCGATGGTT
 501 TCTACAAAGA TCGTTATGTT TATCGGCACT TTGCATCGGC CGCGCTCCCG ATTCCGGAAG TGCTTGACAT TGGGGAATTC AGCGAGAGCC TGACCTATTG
 601 CATCTCCCGC CGTGCACAGG GTGTCACGTT GCAAGACCTG CCTGAAACCG AACTGCCCGC TGTTCTGCAG CCGGTCGCGG AGGCCATGGA TGCGATCGCT
 701 GCGGCCGATC TTAGCCAGAC GAGCGGGTTC GGCCCATTCG GACCGCAAGG AATCGGTCAA TACACTACAT GGCGTGATTT CATATGCGCG ATTGCTGATC
 801 CCCATGTGTA TCACTGGCAA ACTGTGATGG ACGACACCGT CAGTGCGTCC GTCGCGCAGG CTCTCGATGA GCTGATGCTT TGGGCCGAGG ACTGCCCCGA
 901 AGTCCGGCAC CTCGTGCACG CGGATTTCGG CTCCAACAAT GTCCTGACGG ACAATGGCCG CATAACAGCG GTCATTGACT GGAGCGAGGC GATGTTCGGG
1001 GATTCCCAAT ACGAGGTCGC CAACATCTTC TTCTGGAGGC CGTGGTTGGC TTGTATGGAG CAGCAGACGC GCTACTTCGA GCGGAGGCAT CCGGAGCTTG
1101 CAGGATCGCC GCGGCTCCGG GCGTATATGC TCCGCATTGG TCTTGACCAA CTCTATCAGA GCTTGGTTGA CGGCAATTTC GATGATGCAG CTTGGGCGCA
1201 GGGTCGATGC GACGCAATCG TCCGATCCGG AGCCGGGACT GTCGGGCGTA CACAAATCGC CCGCAGAAGC GCGGCCGTCT GGACCGATGG CTGTGTAGAA
1301 GTACTCGCCG ATAGTGGAAA CCGACGCCCC AGCACTCGTC CGAGGGCAAA GGAATAGAGT AGATGCCGAC CGGAGTCCGC AAAAATCACC AGTCTCTCTC
1401 TACAAATCTA TCTCTCTCTA TTTTTCTCCA GAATAATGTG TGAGTAGTTC CCAGATAAGG GAATTAGGGT TCTTATAGGG TTTCGCTCAT GTGTTGAGCA
1501 TATAAGAAAC CCTTAGTATG TATTTGTATT TGTAAAATAC TTCTATCAAT AAAATTTCTA ATTCCTAAAA CCAAAATCCA GTGACCTGCA GGCATGCAAG
1601 CTGATCCACT AGAGGCCATG GCGGCCGCTT TCCTTACATT CTGAGCCTCT TTCCTTCTAA TCCACTCATC TGCATCTTCT TGTGTCCTTA CTAATACCTC
1701 ATTGGTTCCA AATTCCCTCC CTTTAAGCAC CAGCTCGTTT CTGTTCTTCC ACAGCCTCCC AAGTATCCAA GGGACTAAAG CCTCCACATT CTTCAGATCA
1801 GGATATTCTT GTTTAAGATG TTGAACTCTA TGGAGGTTTG TATGAACTGA TGATCTAGGA CCGGATAAGT TCCCTTCTTC ATAGCGAACT TATTCAAAGA
1901 ATGTTTTGTG TATCATTCTT GTTACATTGT TATTAATGAA AAAATATTAT TGGTCATTGG ACTGAACACG AGTGTTAAAT ATGGACCAGG CCCCAAATAA
2001 GATCCATTGA TATATGAATT AAATAACAAG AATAAATCGA GTCACCAAAC CACTTGCCTT TTTTAACGAG ACTTGTTCAC CAACTTGATA CAAAAGTCAT
2101 TATCCTATGC AAATCAATAA TCATACAAAA ATATCCAATA ACACTAAAAA ATTAAAAGAA ATGGATAATT TCACAATATG TTATACGATA AAGAAGTTAC
2201 TTTTCCAAGA AATTCACTGA TTTTATAAGC CCACTTGCAT TAGATAAATG GCAAAAAAAA ACAAAAAGGA AAAGAAATAA AGCACGAAGA ATTCTAGAAA
2301 ATACGAAATA CGCTTCAATG CAGTGGGACC CACGGTTCAA TTATTGCCAA TTTTCAGCTC CACCGTATAT TTAAAAAATA AAACGATAAT GCTAAAAAAA
2401 TATAAATCGT AACGATCGTT AAATCTCAAC GGCTGGATCT TATGACGACC GTTAGAAATT GTGGTTGTCG ACGAGTCAGT AATAAACGGC GTCAAAGTGG
2501 TTGCAGCCGG CACACACGAG TCGTGTTTAT CAACTCAAAG CACAAATACT TTTCCTCAAC CTAAAAATAA GGCAATTAGC CAAAAACAAC TTTGCGTGTA
2601 AACAACGCTC AATACACGTG TCATTTTATT ATTAGCTATT GCTTCACCGC CTTAGCTTTC TCGTGACCTA GTCGTCCTCG TCTTTTCTTC TTCTTCTTCT
2701 ATAAAACAAT ACCCAAAGAG CTCTTCTTCT TCACAATTCA GATTTCAATT TCTCAAAATC TTAAAAACTT TCTCTCAATT CTCTCTACCG TGATCAAGGT
2801 AAATTTCTGT GTTCCTTATT CTCTCAAAAT CTTCGATTTT GTTTTCGTTC GATCCCAATT TCGTATATGT TCTTTGGTTT AGATTCTGTT AATCTTAGAT
2901 CGAAGACGAT TTTCTGGGTT TGATCGTTAG ATATCATCTT AATTCTCGAT TAGGGTTTCA TAGATATCAT CCGATTTGTT CAAATAATTT GAGTTTTGTC
3001 GAATAATTAC TCTTCGATTT GTGATTTCTA TCTAGATCTG GTGTTAGTTT CTAGTTTGTG CGATCGAATT TGTCGATTAA TCTGAGTTTT TCTGATTAAC
3101 AGATACTAGT TCGGCGCGCC ATGGCTCCAA AGAAGAAGAG AAAGGTCAGC GCTGTCACGC GCCAAGCCGC CACCATGGCC GACCCCATTC GTTCGCGCAC
3201 ACCAAGTCCT GCCCGCGAGC TTCTGCCCGG ACCCCAACCC GATGGGGTTC AGCCGACTGC AGATCGTGGG GTGTCTCCGC CTGCCGGCGG CCCCCTGGAT
3301 GGCTTGCCGG CTCGGCGGAC GATGTCCCGG ACCCGGCTGC CATCTCCCCC TGCCCCCTCA CCTGCGTTCT CGGCGGGCAG CTTCAGTGAC CTGTTACGTC
3401 AGTTCGATCC GTCACTTTTT AATACATCGC TTTTTGATTC ATTGCCTCCC TTCGGCGCTC ACCATACAGA GGCTGCCACA GGCGAGTGGG ATGAGGTGCA
3501 ATCGGGTCTG CGGGCAGCCG ACGCCCCCCC ACCCACCATG CGCGTGGCTG TCACTGCCGC GCGGCCCCCG CGCGCCAAGC CGGCGCCGCG ACGACGTGCT
3601 GCGCAACCCT CCGACGCTTC GCCGGCGGCG CAGGTGGATC TACGCACGCT CGGCTACAGC CAGCAGCAAC AGGAGAAGAT CAAACCGAAG GTTCGTTCGA
3701 CAGTGGCGCA GCACCACGAG GCACTGGTCG GCCACGGGTT TACACACGCG CACATCGTTG CGTTAAGCCA ACACCCGGCA GCGTTAGGGA CCGTCGCTGT
3801 CAAGTATCAG GACATGATCG CAGCGTTGCC AGAGGCGACA CACGAAGCGA TCGTTGGCGT CGGCAAACAG TGGTCCGGCG CACGCGCTCT GGAGGCCTTG
3901 CTCACGGTGG CGGGAGAGTT GAGAGGTCCA CCGTTACAGT TGGACACAGG CCAACTTCTC AAGATTGCAA AACGTGGCGG CGTGACCGCA GTGGAGGCAG
4001 TGCATGCATG GCGCAATGCA CTGACGGGTG CCCCGCTCAA CTTGACCCCC CAGCAGGTGG TGGCCATCGC CAGCAATGGC GGTGGCAAGC AGGCGCTGGA
```

4101 GACGGTCCAG CGGCTGTTGC CGGTGCTGTG CCAGGCCCAC GGCTTGACCC CCCAGCAGGT GGTGGCCATC GCCAGCAATA ATGGTGGCAA GCAGGCGCTG
4201 GAGACGGTCC AGCGGCTGTT GCCGGTGCTG TGCCAGGCCC ACGGCTTGAC CCCGGAGCAG GTGGTGGCCA TCGCCAGCCA CGATGGCGGC AAGCAGGCGC
4301 TGGAGACGGT CCAGCGGCTG TTGCCGGTGC TGTGCCAGGC CCACGGCTTG ACCCCGGAGC AGGTGGTGGC CATCGCCAGC CACGATGGCG GCAAGCAGGC
4401 GCTGGAGACG GTCCAGCGGC TGTTGCCGGT GCTGTGCCAG GCCCACGGCT TGACCCCCCA GCAGGTGGTG GCCATCGCCA GCAATAATGG TGGCAAGCAG
4501 GCGCTGGAGA CGGTCCAGCG GCTGTTGCCG GTGCTGTGCC AGGCCCACGG CTTGACCCCC CAGCAGGTGG TGGCCATCGC CAGCAATGGC GGTGGCAAGC
4601 AGGCGCTGGA GACGGTCCAG CGGCTGTTGC CGGTGCTGTG CCAGGCCCAC GGCTTGACCC CGGAGCAGGT GGTGGCCATC GCCAGCCACG ATGGCGGCAA
4701 GCAGGCGCTG GAGACGGTCC AGCGGCTGTT GCCGGTGCTG TGCCAGGCCC ACGGCTTGAC CCCGGAGCAG GTGGTGGCCA TCGCCAGCCA CGATGGCGGC
4801 AAGCAGGCGC TGGAGACGGT CCAGCGGCTG TTGCCGGTGC TGTGCCAGGC CCACGGCTTG ACCCCCCAGC AGGTGGTGGC CATCGCCAGC AATGGCGGTG
4901 GCAAGCAGGC GCTGGAGACG GTCCAGCGGC TGTTGCCGGT GCTGTGCCAG GCCCACGGCT TGACCCCGGA GCAGGTGGTG GCCATCGCCA GCCACGATGG
5001 CGGCAAGCAG GCGCTGGAGA CGGTCCAGCG GCTGTTGCCG GTGCTGTGCC AGGCCCACGG CTTGACCCCG GAGCAGGTGG TGGCCATCGC CAGCCACGAT
5101 GGCGGCAAGC AGGCGCTGGA GACGGTCCAG CGGCTGTTGC CGGTGCTGTG CCAGGCCCAC GGCTTGACCC CCCAGCAGGT GGTGGCCATC GCCAGCAATG
5201 GCGGTGGCAA GCAGGCGCTG GAGACGGTCC AGCGGCTGTT GCCGGTGCTG TGCCAGGCCC ACGGCTTGAC CCCCCAGCAG GTGGTGGCCA TCGCCAGCAA
5301 TGGCGGTGGC AAGCAGGCGC TGGAGACGGT CCAGCGGCTG TTGCCGGTGC TGTGCCAGGC CCACGGCTTG ACCCCCCAGC AGGTGGTGGC CATCGCCAGC
5401 AATAATGGTG GCAAGCAGGC GCTGGAGACG GTCCAGCGGC TGTTGCCGGT GCTGTGCCAG GCCCACGGCT TGACCCCGGA GCAGGTGGTG GCCATCGCCA
5501 GCAATATTGG TGGCAAGCAG GCGCTGGAGA CGGTGCAGGC GCTGTTGCCG GTGCTGTGCC AGGCCCACGG CTTGACCCCT CAGCAGGTGG TGGCCATCGC
5601 CAGCAATGGC GGCGGCAGGC CGGCGCTGGA GAGCATTGTT GCCCAGTTAT CTCGCCCTGA TCCGAGTGGC AGCGGAAGTG GCGGGGATCC TATCAGCCGT
5701 TCCCAGCTGG TGAAGTCCGA GCTGGAGGAG AAGAAATCCG AGTTGAGGCA CAAGCTGAAG TACGTGCCCC ACGAGTACAT CGAGCTGATC GAGATCGCCC
5801 GGAACAGCAC CCAGGACCGT ATCCTGGAGA TGAAGGTGAT GGAGTTCTTC ATGAAGGTGT ACGGCTACAG GGGCAAGCAC CTGGGCGGCT CCAGGAAGCC
5901 CGACGGCGCC ATCTACACCG TGGGCTCCCC CATCGACTAC GGCGTGATCG TGGACACCAA GGCCTACTCC GGCGGCTACA ACCTGCCCAT CGGCCAGGCC
6001 GACGAAATGC AGAGGTACGT GGAGGAGAAC CAGACCAGGA ACAAGCACAT CAACCCCAAC GAGTGGTGGA AGGTGTACCC CTCCAGCGTG ACCGAGTTCA
6101 AGTTCCTGTT CGTGTCCGGC CACTTCAAGG GCAACTACAA GGCCCAGCTG ACCAGGCTGA ACCACATCAC CAACTGCAAC GGCGCCGTGC TGTCCGTGGA
6201 GGAGCTCCTG ATCGGCGGCG AGATGATCAA GGCCGGCACC CTGACCCTGG AGGAGGTGAG GAGGAAGTTC AACAACGGCG AGATCAACTT CGCGGCCGAC
6301 TGATAACTCG AGCGATCCTC TAGACGAGCT CCTCGAGCCT GCAGCAGCTG AAGCTTACGC GTGTCGACCC TGCTTTAATG AGATATGCGA GACGCCTATG
6401 ATCGCATGAT ATTTGCTTTC AATTCTGTTG TGCACGTTGT AAAAAACCTG AGCATGTGTA GCTCAGATCC TTACCGCCGG TTTCGGTTCA TTCTAATGAA
6501 TATATCACCC GTTACTATCG TATTTTTATG AATAATATTC TCCGTTCAAT TTACTGATTG TCCTCGACAG GCCTTAAGGG CCAGATCTTG GGCCCGGTAC
6601 CCGATCAGAT TGTCGTTTCC CGCCTTCGGT TTAAACTATC AGTGTTTGAC AGGATATATT GGCGGGTAAA CCTAAGAGAA AAGAGCGTTT ATTAGAATAA
6701 TCGGATATTT AAAAGGGCGT GAAAAGGTTT ATCCGTTCGT CCATTTGTAT GTGCATGCCA ACCACAGGGT TCCCCTCGGG AGTGCTTGGC ATTCCGTGCG
6801 ATAATGACTT CTGTTCAACC ACCCAAACGT CGGAAAGCCT GACGACGGAG CAGCATTCCA AAAAGATCCC TTGGCTCGTC TGGGTCGGCT AGAAGGTCGA
6901 GTGGGCTGCT GTGGCTTGAT CCCTCAACGC GGTCGCGGAC GTAGCGCAGC GCCGAAAAAT CCTCGATCGC AAATCCGACG CTGTCGAAAA GCGTGATCTG
7001 CTTGTCGCTC TTTCGGCCGA CGTCCTGGCC AGTCATCACG CGCCAAAGTT CCGTCACAGG ATGATCTGGC GCGAGTTGCT GGATCTCGCC TTCAATCCGG
7101 GTCTGTGGCG GGAACTCCAC GAAAATATCC GAACGCAGCA AGATATCGCG GTGCATCTCG GTCTTGCCTG GGCAGTCGCC GCCGACGCCG TTGATGTGGA
7201 CGCCGAAAAG GATCTAGGTG AAGATCCTTT TTGATAATCT CATGACCAAA ATCCCTTAAC GTGAGTTTTC GTTCCACTGA GCGTCAGACC CCGTAGAAAA
7301 GATCAAAGGA TCTTCTTGAG ATCCTTTTTT TCTGCGCGTA ATCTGCTGCT TGCAAACAAA AAAACCACCG CTACCAGCGG TGGTTTGTTT GCCGGATCAA
7401 GAGCTACCAA CTCTTTTTCC GAAGGTAACT GGCTTCAGCA GAGCGCAGAT ACCAAATACT GTTCTTCTAG TGTAGCCGTA GTTAGGCCAC CACTTCAAGA
7501 ACTCTGTAGC ACCGCCTACA TACCTCGCTC TGCTAATCCT GTTACCAGTG GCTGCTGCCA GTGGCGATAA GTCGTGTCTT ACCGGGTTGG ACTCAAGACG
7601 ATAGTTACCG GATAAGGCGC AGCGGTCGGG CTGAACGGGG GGTTCGTGCA CACAGCCCAG CTTGGAGCGA ACGACCTACA CCGAACTGAG ATACCTACAG
7701 CGTGAGCTAT GAGAAAGCGC CACGCTTCCC GAAGGGAGAA AGGCGGACAG GTATCCGGTA AGCGGCAGGG TCGGAACAGG AGAGCGCACG AGGGAGCTTC
7801 CAGGGGGAAA CGCCTGGTAT CTTTATAGTC CTGTCGGGTT TCGCCACCTC TGACTTGAGC GTCGATTTTT GTGATGCTCG TCAGGGGGGC GGAGCCTATG
7901 GAAAAACGCC AGCAACGCGG CCTTTTTACG GTTCCTGGCC TTTTGCTGGC CTTTTGCTCA CATGTTCTTT CCTGCGTTAT CCCCTGATTC TGTGGATAAC
8001 CGATTACCGC CTTTGAGTGA GCTGATACCG CTCGCCGCAG CCGAACGACC GAGCGCAGCG AGTCAGTGAG CGAGGAAGCG GAAGAGCGCC TGATGCGGTA
8101 TTTTCTCCTT ACGCATCTGT GCGGTATTTC ACACCGCATA TGGTGCACTC TCAGTACAAT CTGCTCTGAT GCCGCATAGT TAAGCCAGTA TACACTCCGC
8201 TATCGCTACG TGACTGGGTC ATGGCTGCGC CCCGACACCC GCCAACACCC GCTGACGCGC CCTGACGGGC TTGTCTGCTC CCGGCATCCG CTTACAGACA
8301 AGCTGTGACC GTCTCCGGGA GCTGCATGTG TCAGAGGTTT TCACCGTCAT CACCGAAACG CGCGAGGCAG GGGTACGTCG AGGTCGATCC AACCCCTCCG
8401 CTGCTATAGT GCAGTCGGCT TCTGACGTTC AGTGCAGCCG TCTTCTGAAA ACGACATGTC GCACAAGTCC TAAGTTACGC GACAGGCTGC CGCCCTGCCC
8501 TTTTCCTGGC GTTTTCTTGT CGCGTGTTTT AGTCGCATAA AGTAGAATAC TTGCGACTAG AACCGGAGAC ATTACGCCAT GAACAAGAGC GCCGCCGCTG
8601 GCCTGCTGGG CTATGCCCGC GTCAGCACCG ACGACCAGGA CTTGACCAAC CAACGGGCCG AACTGCACGC GGCCGGCTGC ACCAAGCTGT TTTCCGAGAA
8701 GATCACCGGC ACCAGGCGCG ACCGCCCGGA GCTGGCCAGG ATGCTTGACC ACCTACGCCC TGGCGACGTT GTGACAGTGA CCAGGCTAGA CCGCCTGGCC
8801 CGCAGCACCC GCGACCTACT GGACATTGCC GAGCGCATCC AGGAGGCCGG CGCGGGCCTG CGTAGCCTGG CAGAGCCGTG GGCCGACACC ACCACGCCGG
8901 CCGGCCGCAT GGTGTTGACC GTGTTCGCCG GCATTGCCGA GTTCGAGCGT TCCCTAATCA TCGACCGCAC CCGGAGCGGG CGCGAGGCCG CCAAGGCGCG
9001 AGGCGTGAAG TTTGGCCCCC GCCCTACCCT CACCCCGGCA CAGATCGCGC ACGCCCGCGA GCTGATCGAC CAGGAAGGCC GCACCGTGAA AGAGGCGGCT
9101 GCACTGCTTG GCGTGCATCG CTCGACCCTG TACCGCGCAC TTGAGCGCAG CGAGGAAGTG ACGCCCACCG AGGCCAGGCG GCGCGGTGCC TTCCGTGAGG
9201 ACGCATTGAC CGAGGCCGAC GCCCTGGCGG CCGCCGAGAA TGAACGCCAA GAGGAACAAG CATGAAACCG CACCAGGACG GCCAGGACGA ACCGTTTTTC
9301 ATTACCGAAG AGATCGAGGC GGAGATGATC GCGGCCGGGT ACGTGTTCGA GCCGCCCGCG CACGTCTCAA CCGTGCGGCT GCATGAAATC CTGGCCGGTT
9401 TGTCTGATGC CAAGCTCGCG GCCTGGCCGG CGAGCTTGGC CGCTGAAGAA ACCGAGCGCC GCCGTCTAAA AAGGTGATGT GTATTTGAGT AAAACAGCTT
9501 GCGTCATGCG GTCGCTGCGT ATATGATGCG ATGAGTAAAT AAACAAATAC GCAAGGGGAA CGCATGAAGG TTATCGCTGT ACTTAACCAG AAAGGCGGGT
9601 CAGGCAAGAC GACCATCGCA ACCCATCTAG CCCGCGCCCT GCAACTCGCC GGGGCCGATG TTCTGTTAGT CGATTCCGAT CCCCAGGGCA GTGCCCGCGA
9701 TTGGGCGGCC GTGCGGGAAG ATCAACCGCT AACCGTTGTC GGCATCGACC GCCCGACGAT TGACCGCGAC GTGAAGGCCA TCGGCCGGCG CGACTTCGTA
9801 GTGATCGACG GAGCGCCCCA GGCGGCGGAC TTGGCTGTGT CCGCGATCAA GGCAGCCGAC TTCGTGCTGA TTCCGGTGCA GCCAAGCCCT TACGACATAT
9901 GGGCCACCGC CGACCTGGTG GAGCTGGTTA AGCAGCGCAT TGAGGTCACG GATGGAAGGC TACAAGCGGC CTTTGTCGTG TCGCGGGCGA TCAAAGGCAC
10001 GCGCATCGGC GGTGAGGTTG CCGAGGCGCT GGCCGGGTAC GAGCTGCCCA TTCTTGAGTC CCGTATCACG CAGCGCGTGA GCTACCCAGG CACTGCCGCC
10101 GCCGGCACAA CCGTTCTTGA ATCAGAACCC GAGGGCGACG CTGCCCGCGA GGTCCAGGCG CTGGCCGCTG AAATTAAATC AAAACTCATT TGAGTTAATG
10201 AGGTAAAGAG AAAATGAGCA AAAGCACAAA CACGCTAAGT GCCGGCCGTC CGAGCGCACG CAGCAGCAAG GCTGCAACGT TGGCCAGCCT GGCAGACACG
10301 CCAGCCATGA AGCGGGTCAA CTTTCAGTTG CCGGCGGAGG ATCACACCAA GCTGAAGATG TACGCGGTAC GCCAAGGCAA GACCATTACC GAGCTGCTAT
10401 CTGAATACAT CGCGCAGCTA CCAGAGTAAA TGAGCAAATG AATAAATGAG TAGATGAATT TTAGCGGCTA AAGGAGGCGG CATGGAAAAT CAAGAACAAC
10501 CAGGCACCGA CGCCGTGGAA TGCCCCATGT GTGGAGGAAC GGGCGGTTGG CCAGGCGTAA GCGGCTGGGT TGTCTGCCGG CCCTGCAATG GCACTGGAAC
10601 CCCCAAGCCC GAGGAATCGG CGTGAGCGGT CGCAAACCAT CCGGCCCGGT ACAAATCGGC GCGGCGCTGG GTGATGACCT GGTGGAGAAG TTGAAGGCGG
10701 CGCAGGCCGC CCAGCGGCAA CGCATCGAGG CAGAAGCACG CCCCGGTGAA TCGTGGCAAG CGGCCGCTGA TCGAATCCGC AAAGAATCCC GGCAACCGCC
10801 GGCAGCCGGT GCGCCGTCGA TTAGGAAGCC GCCCAAGGGC GACGAGCAAC CAGATTTTTT CGTTCCGATG CTCTATGACG TGGGCACCCG CGATAGTCGC
10901 AGCATCATGG ACGTGGCCGT TTTCCGTCTG TCGAAGCGTG ACCGACGAGC TGGCGAGGTG ATCCGCTACG AGCTTCCAGA CGGGCACGTA GAGGTTTCCG
11001 CAGGGCCGGC CGGCATGGCG AGTGTGTGGG ATTACGACCT GGTACTGATG GCGGTTTCCC ATCTAACCGA ATCCATGAAC CGATACCGGG AAGGGAAGGG
11101 AGACAAGCCC GGCCGCGTGT TCCGTCCACA CGTTGCGGAC GTACTCAAGT TCTGCCGGCG AGCCGATGGC GGAAAGCAGA AAGACGACCT GGTAGAAACC
11201 TGCATTCGGT TAAACACCAC GCACGTTGCC ATGCAGCGTA CGAAGAAGGC CAAGAACGGC CGCCTGGTGA CGGTATCCGA GGGTGAAGCC TTGATTAGCC
11301 GCTACAAGAT CGTAAAGAGC GAAACCGGGC GGCCGGAGTA CATCGAGATC GAGCTAGCTG ATTGGATGTA CCGCGAGATC ACAGAAGGCA AGAACCCGGA
11401 CGTGCTGACG GTTCACCCCG ATTACTTTTT GATCGATCCC GGCATCGGCC GTTTTCTCTA CCGCCTGGCA CGCCGCGCCG CAGGCAAGGC AGAAGCCAGA
11501 TGGTTGTTCA AGACGATCTA CGAACGCAGT GGCAGCGCCG GAGAGTTCAA GAAGTTCTGT TTCACCGTGC GCAAGCTGAT CGGGTCAAAT GACCTGCCGG
11601 AGTACGATTT GAAGGAGGAG GCGGGGCAGG CTGGCCCGAT CCTAGTCATG CGCTACCGCA ACCTGATCGA GGGCGAAGCA TCCGCCGGTT CCTAATGTAC
11701 GGAGCAGATG CTAGGGCAAA TTGCCCTAGC AGGGGAAAAA GGTCGAAAAG GTCTCTTTCC TGTGGATAGC ACGTACATTG GGAACCCAAA GCCGTACATT
11801 GGGAACCGGA ACCCGTACAT TGGGAACCCA AAGCCGTACA TTGGGAACCG GTCACACATG TAAGTGACTG ATATAAAAGA GAAAAAAGGC GATTTTTCCG
11901 CCTAAAACTC TTTAAAACTT ATTAAAACTC TTAAAACCCG CCTGGCCTGT GCATAACTGT CTGGCCAGCG CACAGCCGAA GAGCTGCAAA AAGCGCCTAC
12001 CCTTCGGTCG CTGCGCTCCC TACGCCCCGC CGCTTCGCGT CGGCCTATCG CGGCCGCTGG CCGCTCAAAA ATGGCTGGCC TACGGCCAGG CAATCTACCA
12101 GGGCGCGGAC AAGCCGCGCC GTCGCCACTC GACCGCCGGC GCCCACATCA AGGCACCGGT GGGTATGCCT GACGATGCGT GGAGACCGAA ACCTTGCGCT
12201 CGTTCGCCAG CCAGGACAGA AATGCCTCGA CTTCGCTGCT GCCCAAGGTT GCCGGGTGAC GCACACCGTG GAAACGGATG AAGGCACGAA CCCAGTGGAC
12301 ATAAGCCTGT TCGGTTCGTA AGCTGTAATG CAAGTAGCGT ATGCGCTCAC GCAACTGGTC CAGAACCTTG ACCGAACGCA GCGGTGGTAA CGGCGCAGTG
12401 GCGGTTTTCA TGGCTTGTTA TGACTGTTTT TTTGGGGTAC AGTCTATGCC TCGGGCATCC AAGCAGCAAG CGCGTTACGC CGTGGGTCGA TGTTTGATGT
12501 TATGGAGCAG CAACGATGTT ACGCAGCAGG GCAGTCGCCC TAAAACAAAG TTAAACATCA TGAGGGAAGC GGTGATCGCC GAAGTATCGA CTCAACTATC
12601 AGAGGTAGTT GGCGTCATCG AGCGCCATCT CGAACCGACG TTGCTGGCCG TACATTTGTA CGGCTCCGCA GTGGATGGCG GCCTGAAGCC ACACAGTGAT
12701 ATTGATTTGC TGGTTACGGT GACCGTAAGG CTTGATGAAA CAACGCGGCG AGCTTTGATC AACGACCTTT TGGAAACTTC GGCTTCCCCT GGAGAGAGCG
12801 AGATTCTCCG CGCTGTAGAA GTCACCATTG TTGTGCACGA CGACATCATT CCGTGGCGTT ATCCAGCTAA GCGCGAACTG CAATTTGGAG AATGGCAGCG
12901 CAATGACATT CTTGCAGGTA TCTTCGAGCC AGCCACGATC GACATTGATC TGGCTATCTT GCTGACAAAA GCAAGAGAAC ATAGCGTTGC CTTGGTAGGT
13001 CCAGCGGCGG AGGAACTCTT TGATCCGGTT CCTGAACAGG ATCTATTTGA GGCGCTAAAT GAAACCTTAA CGCTATGGAA CTCGCCGCCC GACTGGGCTG
13101 GCGATGAGCG AAATGTAGTG CTTACGTTGT CCCGCATTTG GTACAGCGCA GTAACCGGCA AAATCGCGCC GAAGGATGTC GCTGCCGACT GGGCAATGGA
13201 GCGCCTGCCG GCCCAGTATC AGCCCGTCAT ACTTGAAGCT AGACAGGCTT ATCTTGGACA AGAAGAAGAT CGCTTGGCCT CGCGCGCAGA TCAGTTGGAA
13301 GAATTTGTCC ACTACGTGAA AGGCGAGATC ACCAAGGTAG TCGGCAAATA ATGTCTAACA ATTCGTTCAA GCCGACGCCG CTTCGCGGCG CGGCTTAACT
13401 CAAGCGTTAG ATGCACTAAG CACATAATTG CTCACAGCCA AACTATCAGG TCAAGTCTGC TTTTATTATT TTTAAGCGTG CATAATAAGC CCTACACAAA
13501 TTGGGAGATA TATCATGAAA GGCTGGCTTT TTCTTGTTAT CGCAATAGTT GGCGAAGTAA TCGCAACATA GCTTGCTTGG TCGTTCCGCG TGAACGTCGG
13601 CTCGATTGTA CCTGCGTTCA AATACTTTGC GATCGTGTTG CGCGCCTGCC CGGTGCGTCG GCTGATCTCA CGGATCGACT GCTTCTCTCG CAACGCCATC
13701 CGACGGATGA TGTTTAAAAG TCCCATGTGG ATCACTCCGT TGCCCCGTCG CTCACCGTGT TGGGGGGAAG GTGCACATGG CTCAGTTCTC AATGGAAATT
13801 ATCTGCCTAA CCGGCTCAGT TCTGCGTAGA AACCAACATG CAAGCTCCAC CGGGTGCAAA GCGGCAGCGG CGGCAGGATA TATTCAATTG TAAATGGCTT
13901 CATGTCCGGG AAATCTACAT GGATCAGCAA TGAGTATGAT GGTCAATATG GAGAAAAAGA AAGAGTAATT ACCAATTTTT TTTCAATTCA AAAATGTAGA
14001 TGTCCGCAGC GTTATTATAA AATGAAAGTA CATTTTGATA AAACGACAAA TTACGATCCG TCGTATTTAT AGGCGAAAGC AATAAACAAA TTATTCTAAT
14101 TCGGAAATCT TTATTTCGAC GTGTCTACAT TCACGTCCAA ATGGGGGCTT AGATGAGAAA CTTCACGATC GGC

gfp-specific right TALEN vector used in tobacco pSP11

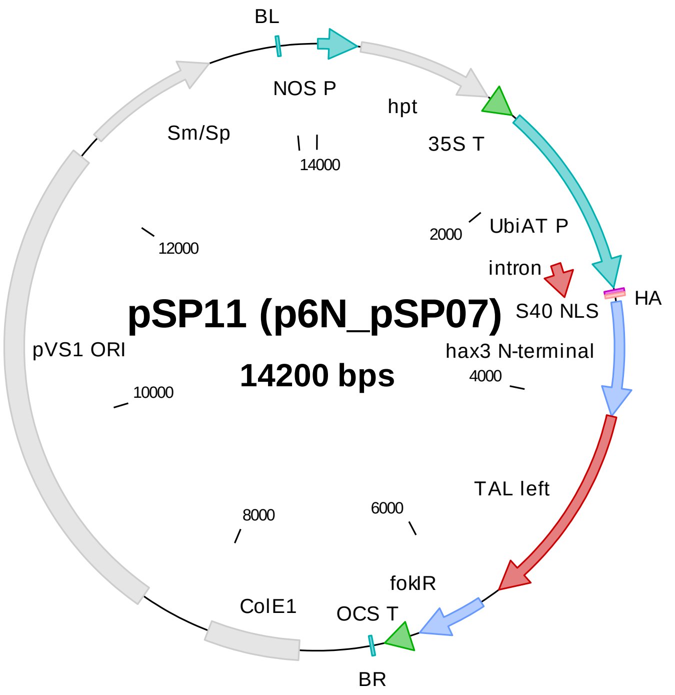


```
   1 TCTAGATCAT GAGCGGAGAA TTAAGGGAGT CACGTTATGA CCCCCGCCGA TGACGCGGGA CAAGCCGTTT TACGTTTGGA ACTGACAGAA CCGCAACGTT
 101 GAAGGAGCCA CTCAGCCGCG GGTTTCTGGA GTTTAATGAG CTAAGCACAT ACGTCAGAAA CCATTATTGC GCGTTCAAAA GTCGCCTAAG GTCACTATCA
 201 GCTAGCAAAT ATTTCTTGTC AAAAATGCTC CACTGACGTT CCATAAATTC CCCTCGGTAT CCAATTAGAG TCTCATATTC ACTCTCAATC CAAATAATCT
 301 GCACCTCGAG GGCCCCGGGG GGCAATAAGA TATGAAAAAG CCTGAACTCA CCGCGACGTC TGTCGAGAAG TTTCTGATCG AAAAGTTCGA CAGCGTCTCC
 401 GACCTGATGC AGCTCTCGGA GGGCGAAGAA TCTCGTGCTT TCAGCTTCGA TGTAGGAGGG CGTGGATATG TCCTGCGGGT AAATAGCTGC GCCGATGGTT
 501 TCTACAAAGA TCGTTATGTT TATCGGCACT TTGCATCGGC CGCGCTCCCG ATTCCGGAAG TGCTTGACAT TGGGGAATTC AGCGAGAGCC TGACCTATTG
 601 CATCTCCCGC CGTGCACAGG GTGTCACGTT GCAAGACCTG CCTGAAACCG AACTGCCCGC TGTTCTGCAG CCGGTCGCGG AGGCCATGGA TGCGATCGCT
 701 GCGGCCGATC TTAGCCAGAC GAGCGGGTTC GGCCCATTCG GACCGCAAGG AATCGGTCAA TACACTACAT GGCGTGATTT CATATGCGCG ATTGCTGATC
 801 CCCATGTGTA TCACTGGCAA ACTGTGATGG ACGACACCGT CAGTGCGTCC GTCGCGCAGG CTCTCGATGA GCTGATGCTT TGGGCCGAGG ACTGCCCCGA
 901 AGTCCGGCAC CTCGTGCACG CGGATTTCGG CTCCAACAAT GTCCTGACGG ACAATGGCCG CATAACAGCG GTCATTGACT GGAGCGAGGC GATGTTCGGG
1001 GATTCCCAAT ACGAGGTCGC CAACATCTTC TTCTGGAGGC CGTGGTTGGC TTGTATGGAG CAGCAGACGC GCTACTTCGA GCGGAGGCAT CCGGAGCTTG
1101 CAGGATCGCC GCGGCTCCGG GCGTATATGC TCCGCATTGG TCTTGACCAA CTCTATCAGA GCTTGGTTGA CGGCAATTTC GATGATGCAG CTTGGGCGCA
1201 GGGTCGATGC GACGCAATCG TCCGATCCGG AGCCGGGACT GTCGGGCGTA CACAAATCGC CCGCAGAAGC GCGGCCGTCT GGACCGATGG CTGTGTAGAA
1301 GTACTCGCCG ATAGTGGAAA CCGACGCCCC AGCACTCGTC CGAGGGCAAA GGAATAGAGT AGATGCCGAC CGGAGTCCGC AAAAATCACC AGTCTCTCTC
1401 TACAAATCTA TCTCTCTCTA TTTTTCTCCA GAATAATGTG TGAGTAGTTC CCAGATAAGG GAATTAGGGT TCTTATAGGG TTTCGCTCAT GTGTTGAGCA
1501 TATAAGAAAC CCTTAGTATG TATTTGTATT TGTAAAATAC TTCTATCAAT AAAATTTCTA ATTCCTAAAA CCAAAATCCA GTGACCTGCA GGCATGCAAG
1601 CTGATCCACT AGAGGCCATG GCGGCCGCTT TCCTTACATT CTGAGCCTCT TTCCTTCTAA TCCACTCATC TGCATCTTCT TGTGTCCTTA CTAATACCTC
1701 ATTGGTTCCA AATTCCCTCC CTTTAAGCAC CAGCTCGTTT CTGTTCTTCC ACAGCCTCCC AAGTATCCAA GGGACTAAAG CCTCCACATT CTTCAGATCA
1801 GGATATTCTT GTTTAAGATG TTGAACTCTA TGGAGGTTTG TATGAACTGA TGATCTAGGA CCGGATAAGT TCCCTTCTTC ATAGCGAACT TATTCAAAGA
1901 ATGTTTTGTG TATCATTCTT GTTACATTGT TATTAATGAA AAAATATTAT TGGTCATTGG ACTGAACACG AGTGTTAAAT ATGGACCAGG CCCCAAATAA
2001 GATCCATTGA TATATGAATT AAATAACAAG AATAAATCGA GTCACCAAAC CACTTGCCTT TTTTAACGAG ACTTGTTCAC CAACTTGATA CAAAAGTCAT
2101 TATCCTATGC AAATCAATAA TCATACAAAA ATATCCAATA ACACTAAAAA ATTAAAAGAA ATGGATAATT TCACAATATG TTATACGATA AAGAAGTTAC
2201 TTTTCCAAGA AATTCACTGA TTTTATAAGC CCACTTGCAT TAGATAAATG GCAAAAAAAA ACAAAAAGGA AAAGAAATAA AGCACGAAGA ATTCTAGAAA
2301 ATACGAAATA CGCTTCAATG CAGTGGGACC CACGGTTCAA TTATTGCCAA TTTTCAGCTC CACCGTATAT TTAAAAAATA AAACGATAAT GCTAAAAAAA
2401 TATAAATCGT AACGATCGTT AAATCTCAAC GGCTGGATCT TATGACGACC GTTAGAAATT GTGGTTGTCG ACGAGTCAGT AATAAACGGC GTCAAAGTGG
2501 TTGCAGCCGG CACACACGAG TCGTGTTTAT CAACTCAAAG CACAAATACT TTTCCTCAAC CTAAAAATAA GGCAATTAGC CAAAAACAAC TTTGCGTGTA
2601 AACAACGCTC AATACACGTG TCATTTTATT ATTAGCTATT GCTTCACCGC CTTAGCTTTC TCGTGACCTA GTCGTCCTCG TCTTTTCTTC TTCTTCTTCT
2701 ATAAAACAAT ACCCAAAGAG CTCTTCTTCT TCACAATTCA GATTTCAATT TCTCAAAATC TTAAAAACTT TCTCTCAATT CTCTCTACCG TGATCAAGGT
2801 AAATTTCTGT GTTCCTTATT CTCTCAAAAT CTTCGATTTT GTTTTCGTTC GATCCCAATT TCGTATATGT TCTTTGGTTT AGATTCTGTT AATCTTAGAT
2901 CGAAGACGAT TTTCTGGGTT TGATCGTTAG ATATCATCTT AATTCTCGAT TAGGGTTTCA TAGATATCAT CCGATTTGTT CAAATAATTT GAGTTTTGTC
3001 GAATAATTAC TCTTCGATTT GTGATTTCTA TCTAGATCTG GTGTTAGTTT CTAGTTTGTG CGATCGAATT TGTCGATTAA TCTGAGTTTT TCTGATTAAC
3101 AGATACTAGT TCGGCGCGCC ATGGCTCCAA AGAAGAAGAG AAAGGTCTAC CCATACGACG TCCCAGACTA CGCTAGCGCT GTCACGCGCC AAGCCGCCAC
3201 CATGGCCGAC CCCATTCGTT CGCGCACACC AAGTCCTGCC CGCGAGCTTC TGCCCGGACC CCAACCCGAT GGGGTTCAGC CGACTGCAGA TCGTGGGGTG
3301 TCTCCGCCTG CCGGCGGCCC CCTGGATGGC TTGCCGGCTC GGCGGACGAT GTCCCGGACC CGGCTGCCAT CTCCCCCTGC CCCCTCACCT GCGTTCTCGG
3401 CGGGCAGCTT CAGTGACCTG TTACGTCAGT TCGATCCGTC ACTTTTTAAT ACATCGCTTT TTGATTCATT GCCTCCCTTC GGCGCTCACC ATACAGAGGC
3501 TGCCACAGGC GAGTGGGATG AGGTGCAATC GGGTCTGCGG GCAGCCGACG CCCCCCCACC CACCATGCGC GTGGCTGTCA CTGCCGCGCG GCCCCCGCGC
3601 GCCAAGCCGG CGCCGCGACG ACGTGCTGCG CAACCCTCCG ACGCTTCGCC GGCGGCGCAG GTGGATCTAC GCACGCTCGG CTACAGCCAG CAGCAACAGG
3701 AGAAGATCAA ACCGAAGGTT CGTTCGACAG TGGCGCAGCA CCACGAGGCA CTGGTCGGCC ACGGGTTTAC ACACGCGCAC ATCGTTGCGT TAAGCCAACA
3801 CCCGGCAGCG TTAGGGACCG TCGCTGTCAA GTATCAGGAC ATGATCGCAG CGTTGCCAGA GGCGACACAC GAAGCGATCG TTGGCGTCGG CAAACAGTGG
3901 TCCGGCGCAC GCGCTCTGGA GGCCTTGCTC ACGGTGGCGG GAGAGTTGAG AGGTCCACCG TTACAGTTGG ACACAGGCCA ACTTCTCAAG ATTGCAAAAC
4001 GTGGCGGCGT GACCGCAGTG GAGGCAGTGC ATGCATGGCG CAATGCACTG ACGGGTGCCC CGCTCAACTT GACCCCCCAG CAGGTGGTGG CCATCGCCAG
4101 CAATAATGGT GGCAAGCAGG CGCTGGAGAC GGTCCAGCGG CTGTTGCCGG TGCTGTGCCA GGCCCACGGC TTGACCCCGG AGCAGGTGGT GGCCATCGCC
```

4201 AGCAATATTG GTGGCAAGCA GGCGCTGGAG ACGGTGCAGG CGCTGTTGCC GGTGCTGTGC CAGGCCCACG GCTTGACCCC GGAGCAGGTG GTGGCCATCG
4301 CCAGCAATAT TGGTGGCAAG CAGGCGCTGG AGACGGTGCA GGCGCTGTTG CCGGTGCTGT GCCAGGCCCA CGGCTTGACC CCGGAGCAGG TGGTGGCCAT
4401 CGCCAGCCAC GATGGCGGCA AGCAGGCGCT GGAGACGGTC CAGCGGCTGT TGCCGGTGCT GTGCCAGGCC CACGGCTTGA CCCCGGAGCA GGTGGTGGCC
4501 ATCGCCAGCC ACGATGGCGG CAAGCAGGCG CTGGAGACGG TCCAGCGGCT GTTGCCGGTG CTGTGCCAGG CCCACGGCTT GACCCCCCAG CAGGTGGTGG
4601 CCATCGCCAG CAATAATGGT GGCAAGCAGG CGCTGGAGAC GGTCCAGCGG CTGTTGCCGG TGCTGTGCCA GGCCCACGGC TTGACCCCGG AGCAGGTGGT
4701 GGCCATCGCC AGCCACGATG GCGGCAAGCA GGCGCTGGAG ACGGTCCAGC GGCTGTTGCC GGTGCTGTGC CAGGCCCACG GCTTGACCCC GGAGCAGGTG
4801 GTGGCCATCG CCAGCAATAT TGGTGGCAAG CAGGCGCTGG AGACGGTGCA GGCGCTGTTG CCGGTGCTGT GCCAGGCCCA CGGCTTGACC CCCCAGCAGG
4901 TGGTGGCCAT CGCCAGCAAT GGCGGTGGCA AGCAGGCGCT GGAGACGGTC CAGCGGCTGT TGCCGGTGCT GTGCCAGGCC CACGGCTTGA CCCCGGAGCA
5001 GGTGGTGGCC ATCGCCAGCC ACGATGGCGG CAAGCAGGCG CTGGAGACGG TCCAGCGGCT GTTGCCGGTG CTGTGCCAGG CCCACGGCTT GACCCCCCAG
5101 CAGGTGGTGG CCATCGCCAG CAATAATGGT GGCAAGCAGG CGCTGGAGAC GGTCCAGCGG CTGTTGCCGG TGCTGTGCCA GGCCCACGGC TTGACCCCGG
5201 AGCAGGTGGT GGCCATCGCC AGCAATATTG GTGGCAAGCA GGCGCTGGAG ACGGTGCAGG CGCTGTTGCC GGTGCTGTGC CAGGCCCACG GCTTGACCCC
5301 CCAGCAGGTG GTGGCCATCG CCAGCAATAA TGGTGGCAAG CAGGCGCTGG AGACGGTCCA GCGGCTGTTG CCGGTGCTGT GCCAGGCCCA CGGCTTGACC
5401 CCGGAGCAGG TGGTGGCCAT CGCCAGCCAC GATGGCGGCA AGCAGGCGCT GGAGACGGTC CAGCGGCTGT TGCCGGTGCT GTGCCAGGCC CACGGCTTGA
5501 CCCCCCAGCA GGTGGTGGCC ATCGCCAGCA ATGGCGGTGG CAAGCAGGCG CTGGAGACGG TCCAGCGGCT GTTGCCGGTG CTGTGCCAGG CCCACGGCTT
5601 GACCCCTCAG CAGGTGGTGG CCATCGCCAG CAATGGCGGC GGCAGGCCGG CGCTGGAGAG CATTGTTGCC CAGTTATCTC GCCCTGATCC GAGTGGCAGC
5701 GGAAGTGGCG GGGATCCTAT CAGCCGTTCC CAGCTGGTGA AGTCCGAGCT GGAGGAGAAG AAATCCGAGT TGAGGCACAA GCTGAAGTAC GTGCCCCACG
5801 AGTACATCGA GCTGATCGAG ATCGCCCGGA ACAGCACCCA GGACCGTATC CTGGAGATGA AGGTGATGGA GTTCTTCATG AAGGTGTACG GCTACAGGGG
5901 CAAGCACCTG GGCGGCTCCA GGAAGCCCGA CGGCGCCATC TACACCGTGG GCTCCCCCAT CGACTACGGC GTGATCGTGG ACACCAAGGC CTACTCCGGC
6001 GGCTACAACC TGCCCATCGG CCAGGCCGAC GAAATGCAGA GGTACGTGGA GGAGAACCAG ACCAGGAACA AGCACATCAA CCCCAACGAG TGGTGGAAGG
6101 TGTACCCCTC CAGCGTGACC GAGTTCAAGT TCCTGTTCGT GTCCGGCCAC TTCAAGGGCA ACTACAAGGC CCAGCTGACC AGGCTGAACC ACATCACCAA
6201 CTGCAACGGC GCCGTGCTGT CCGTGGAGGA GCTCCTGATC GGCGGCGAGA TGATCAAGGC CGGCACCCTG ACCCTGGAGG AGGTGAGGAG GAAGTTCAAC
6301 AACGGCGAGA TCAACTTCGC GGCCGACTGA TAACTCGAGC GATCCTCTAG ACGAGCTCCT CGAGCCTGCA GCAGCTGAAG CTTACGCGTG TCGACCCTGC
6401 TTTAATGAGA TATGCGAGAC GCCTATGATC GCATGATATT TGCTTTCAAT TCTGTTGTGC ACGTTGTAAA AAACCTGAGC ATGTGTAGCT CAGATCCTTA
6501 CCGCCGGTTT CGGTTCATTC TAATGAATAT ATCACCCGTT ACTATCGTAT TTTTATGAAT AATATTCTCC GTTCAATTTA CTGATTGTCC TCGACAGGCC
6601 TTAAGGGCCA GATCTTGGGC CCGGTACCCG ATCAGATTGT CGTTTCCCGC CTTCGGTTTA AACTATCAGT GTTTGACAGG ATATATTGGC GGGTAAACCT
6701 AAGAGAAAAG AGCGTTTATT AGAATAATCG GATATTTAAA AGGGCGTGAA AAGGTTTATC CGTTCGTCCA TTTGTATGTG CATGCCAACC ACAGGGTTCC
6801 CCTCGGGAGT GCTTGGCATT CCGTGCGATA ATGACTTCTG TTCAACCACC CAAACGTCGG AAAGCCTGAC GACGGAGCAG CATTCCAAAA AGATCCCTTG
6901 GCTCGTCTGG GTCGGCTAGA AGGTCGAGTG GGCTGCTGTG GCTTGATCCC TCAACGCGGT CGCGGACGTA GCGCAGCGCC GAAAAATCCT CGATCGCAAA
7001 TCCGACGCTG TCGAAAAGCG TGATCTGCTT GTCGCTCTTT CGGCCGACGT CCTGGCCAGT CATCACGCGC CAAAGTTCCG TCACAGGATG ATCTGGCGCG
7101 AGTTGCTGGA TCTCGCCTTC AATCCGGGTC TGTGGCGGGA ACTCCACGAA AATATCCGAA CGCAGCAAGA TATCGCGGTG CATCTCGGTC TTGCCTGGGC
7201 AGTCGCCGCC GACGCCGTTG ATGTGGACGC CGAAAAGGAT CTAGGTGAAG ATCCTTTTTG ATAATCTCAT GACCAAAATC CCTTAACGTG AGTTTTCGTT
7301 CCACTGAGCG TCAGACCCCG TAGAAAAGAT CAAAGGATCT TCTTGAGATC CTTTTTTTCT GCGCGTAATC TGCTGCTTGC AAACAAAAAA ACCACCGCTA
7401 CCAGCGGTGG TTTGTTTGCC GGATCAAGAG CTACCAACTC TTTTTCCGAA GGTAACTGGC TTCAGCAGAG CGCAGATACC AAATACTGTT CTTCTAGTGT
7501 AGCCGTAGTT AGGCCACCAC TTCAAGAACT CTGTAGCACC GCCTACATAC CTCGCTCTGC TAATCCTGTT ACCAGTGGCT GCTGCCAGTG GCGATAAGTC
7601 GTGTCTTACC GGGTTGGACT CAAGACGATA GTTACCGGAT AAGGCGCAGC GGTCGGGCTG AACGGGGGGT TCGTGCACAC AGCCCAGCTT GGAGCGAACG
7701 ACCTACACCG AACTGAGATA CCTACAGCGT GAGCTATGAG AAAGCGCCAC GCTTCCCGAA GGGAGAAAGG CGGACAGGTA TCCGGTAAGC GGCAGGGTCG
7801 GAACAGGAGA GCGCACGAGG GAGCTTCCAG GGGGAAACGC CTGGTATCTT TATAGTCCTG TCGGGTTTCG CCACCTCTGA CTTGAGCGTC GATTTTTGTG
7901 ATGCTCGTCA GGGGGGCGGA GCCTATGGAA AAACGCCAGC AACGCGGCCT TTTTACGGTT CCTGGCCTTT TGCTGGCCTT TTGCTCACAT GTTCTTTCCT
8001 GCGTTATCCC CTGATTCTGT GGATAACCGA TTACCGCCTT TGAGTGAGCT GATACCGCTC GCCGCAGCCG AACGACCGAG CGCAGCGAGT CAGTGAGCGA
8101 GGAAGCGGAA GAGCGCCTGA TGCGGTATTT TCTCCTTACG CATCTGTGCG GTATTTCACA CCGCATATGG TGCACTCTCA GTACAATCTG CTCTGATGCC
8201 GCATAGTTAA GCCAGTATAC ACTCCGCTAT CGCTACGTGA CTGGGTCATG GCTGCGCCCC GACACCCGCC AACACCCGCT GACGCGCCCT GACGGGCTTG
8301 TCTGCTCCCG GCATCCGCTT ACAGACAAGC TGTGACCGTC TCCGGGAGCT GCATGTGTCA GAGGTTTTCA CCGTCATCAC CGAAACGCGC GAGGCAGGGG
8401 TACGTCGAGG TCGATCCAAC CCCTCCGCTG CTATAGTGCA GTCGGCTTCT GACGTTCAGT GCAGCCGTCT TCTGAAAACG ACATGTCGCA CAAGTCCTAA
8501 GTTACGCGAC AGGCTGCCGC CCTGCCCTTT TCCTGGCGTT TTCTTGTCGC GTGTTTTAGT CGCATAAAGT AGAATACTTG CGACTAGAAC CGGAGACATT
8601 ACGCCATGAA CAAGAGCGCC GCCGCTGGCC TGCTGGGCTA TGCCCGCGTC AGCACCGACG ACCAGGACTT GACCAACCAA CGGGCCGAAC TGCACGCGGC
8701 CGGCTGCACC AAGCTGTTTT CCGAGAAGAT CACCGGCACC AGGCGCGACC GCCCGGAGCT GGCCAGGATG CTTGACCACC TACGCCCTGG CGACGTTGTG
8801 ACAGTGACCA GGCTAGACCG CCTGGCCCGC AGCACCCGCG ACCTACTGGA CATTGCCGAG CGCATCCAGG AGGCCGGCGC GGGCCTGCGT AGCCTGGCAG
8901 AGCCGTGGGC CGACACCACC ACGCCGGCCG GCCGCATGGT GTTGACCGTG TTCGCCGGCA TTGCCGAGTT CGAGCGTTCC CTAATCATCG ACCGCACCCG
9001 GAGCGGGCGC GAGGCCGCCA AGGCGCGAGG CGTGAAGTTT GGCCCCCGCC CTACCCTCAC CCCGGCACAG ATCGCGCACG CCCGCGAGCT GATCGACCAG
9101 GAAGGCCGCA CCGTGAAAGA GGCGGCTGCA CTGCTTGGCG TGCATCGCTC GACCCTGTAC CGCGCACTTG AGCGCAGCGA GGAAGTGACG CCCACCGAGG
9201 CCAGGCGGCG CGGTGCCTTC CGTGAGGACG CATTGACCGA GGCCGACGCC CTGGCGGCCG CCGAGAATGA ACGCCAAGAG GAACAAGCAT GAAACCGCAC
9301 CAGGACGGCC AGGACGAACC GTTTTTCATT ACCGAAGAGA TCGAGGCGGA GATGATCGCG GCCGGGTACG TGTTCGAGCC GCCCGCGCAC GTCTCAACCG
9401 TGCGGCTGCA TGAAATCCTG GCCGGTTTGT CTGATGCCAA GCTCGCGGCC TGGCCGGCGA GCTTGGCCGC TGAAGAAACC GAGCGCCGCC GTCTAAAAAG
9501 GTGATGTGTA TTTGAGTAAA ACAGCTTGCG TCATGCGGTC GCTGCGTATA TGATGCGATG AGTAAATAAA CAAATACGCA AGGGGAACGC ATGAAGGTTA
9601 TCGCTGTACT TAACCAGAAA GGCGGGTCAG GCAAGACGAC CATCGCAACC CATCTAGCCC GCGCCCTGCA ACTCGCCGGG GCCGATGTTC TGTTAGTCGA
9701 TTCCGATCCC CAGGGCAGTG CCCGCGATTG GGCGGCCGTG CGGGAAGATC AACCGCTAAC CGTTGTCGGC ATCGACCGCC CGACGATTGA CCGCGACGTG
9801 AAGGCCATCG GCCGGCGCGA CTTCGTAGTG ATCGACGGAG CGCCCCAGGC GGCGGACTTG GCTGTGTCCG CGATCAAGGC AGCCGACTTC GTGCTGATTC
9901 CGGTGCAGCC AAGCCCTTAC GACATATGGG CCACCGCCGA CCTGGTGGAG CTGGTTAAGC AGCGCATTGA GGTCACGGAT GGAAGGCTAC AAGCGGCCTT
10001 TGTCGTGTCG CGGGCGATCA AAGGCACGCG CATCGGCGGT GAGGTTGCCG AGGCGCTGGC CGGGTACGAG CTGCCCATTC TTGAGTCCCG TATCACGCAG
10101 CGCGTGAGCT ACCCAGGCAC TGCCGCCGCC GGCACAACCG TTCTTGAATC AGAACCCGAG GGCGACGCTG CCCGCGAGGT CCAGGCGCTG GCCGCTGAAA
10201 TTAAATCAAA ACTCATTTGA GTTAATGAGG TAAAGAGAAA ATGAGCAAAA GCACAAACAC GCTAAGTGCC GGCCGTCCGA GCGCACGCAG CAGCAAGGCT
10301 GCAACGTTGG CCAGCCTGGC AGACACGCCA GCCATGAAGC GGGTCAACTT TCAGTTGCCG GCGGAGGATC ACACCAAGCT GAAGATGTAC GCGGTACGCC
10401 AAGGCAAGAC CATTACCGAG CTGCTATCTG AATACATCGC GCAGCTACCA GAGTAAATGA GCAAATGAAT AAATGAGTAG ATGAATTTTA GCGGCTAAAG
10501 GAGGCGGCAT GGAAAATCAA GAACAACCAG GCACCGACGC CGTGGAATGC CCCATGTGTG GAGGAACGGG CGGTTGGCCA GGCGTAAGCG GCTGGGTTGT
10601 CTGCCGGCCC TGCAATGGCA CTGGAACCCC CAAGCCCGAG GAATCGGCGT GAGCGGTCGC AAACCATCCG GCCCGGTACA AATCGGCGCG GCGCTGGGTG
10701 ATGACCTGGT GGAGAAGTTG AAGGCGGCGC AGGCCGCCCA GCGGCAACGC ATCGAGGCAG AAGCACGCCC CGGTGAATCG TGGCAAGCGG CCGCTGATCG
10801 AATCCGCAAA GAATCCCGGC AACCGCCGGC AGCCGGTGCG CCGTCGATTA GGAAGCCGCC CAAGGGCGAC GAGCAACCAG ATTTTTTCGT TCCGATGCTC
10901 TATGACGTGG GCACCCGCGA TAGTCGCAGC ATCATGGACG TGGCCGTTTT CCGTCTGTCG AAGCGTGACC GACGAGCTGG CGAGGTGATC CGCTACGAGC
11001 TTCCAGACGG GCACGTAGAG GTTTCCGCAG GGCCGGCCGG CATGGCGAGT GTGTGGGATT ACGACCTGGT ACTGATGGCG GTTTCCCATC TAACCGAATC
11101 CATGAACCGA TACCGGGAAG GGAAGGGAGA CAAGCCCGGC CGCGTGTTCC GTCCACACGT TGCGGACGTA CTCAAGTTCT GCCGGCGAGC CGATGGCGGA
11201 AAGCAGAAAG ACGACCTGGT AGAAACCTGC ATTCGGTTAA ACACCACGCA CGTTGCCATG CAGCGTACGA AGAAGGCCAA GAACGGCCGC CTGGTGACGG
11301 TATCCGAGGG TGAAGCCTTG ATTAGCCGCT ACAAGATCGT AAAGAGCGAA ACCGGGCGGC CGGAGTACAT CGAGATCGAG CTAGCTGATT GGATGTACCG
11401 CGAGATCACA GAAGGCAAGA ACCCGGACGT GCTGACGGTT CACCCCGATT ACTTTTTGAT CGATCCCGGC ATCGGCCGTT TTCTCTACCG CCTGGCACGC
11501 CGCGCCGCAG GCAAGGCAGA AGCCAGATGG TTGTTCAAGA CGATCTACGA ACGCAGTGGC AGCGCCGGAG AGTTCAAGAA GTTCTGTTTC ACCGTGCGCA
11601 AGCTGATCGG GTCAAATGAC CTGCCGGAGT ACGATTTGAA GGAGGAGGCG GGGCAGGCTG GCCCGATCCT AGTCATGCGC TACCGCAACC TGATCGAGGG
11701 CGAAGCATCC GCCGGTTCCT AATGTACGGA GCAGATGCTA GGGCAAATTG CCCTAGCAGG GGAAAAAGGT CGAAAAGGTC TCTTTCCTGT GGATAGCACG
11801 TACATTGGGA ACCCAAAGCC GTACATTGGG AACCGGAACC CGTACATTGG GAACCCAAAG CCGTACATTG GGAACCGGTC ACACATGTAA GTGACTGATA
11901 TAAAAGAGAA AAAAGGCGAT TTTTCCGCCT AAAACTCTTT AAAACTTATT AAAACTCTTA AAACCCGCCT GGCCTGTGCA TAACTGTCTG GCCAGCGCAC
12001 AGCCGAAGAG CTGCAAAAAG CGCCTACCCT TCGGTCGCTG CGCTCCCTAC GCCCCGCCGC TTCGCGTCGG CCTATCGCGG CCGCTGGCCG CTCAAAAATG
12101 GCTGGCCTAC GGCCAGGCAA TCTACCAGGG CGCGGACAAG CCGCGCCGTC GCCACTCGAC CGCCGGCGCC CACATCAAGG CACCGGTGGG TATGCCTGAC
12201 GATGCGTGGA GACCGAAACC TTGCGCTCGT TCGCCAGCCA GGACAGAAAT GCCTCGACTT CGCTGCTGCC CAAGGTTGCC GGGTGACGCA CACCGTGGAA
12301 ACGGATGAAG GCACGAACCC AGTGGACATA AGCCTGTTCG GTTCGTAAGC TGTAATGCAA GTAGCGTATG CGCTCACGCA ACTGGTCCAG AACCTTGACC
12401 GAACGCAGCG GTGGTAACGG CGCAGTGGCG GTTTTCATGG CTTGTTATGA CTGTTTTTTT GGGGTACAGT CTATGCCTCG GGCATCCAAG CAGCAAGCGC
12501 GTTACGCCGT GGGTCGATGT TTGATGTTAT GGAGCAGCAA CGATGTTACG CAGCAGGGCA GTCGCCCTAA AACAAAGTTA AACATCATGA GGGAAGCGGT
12601 GATCGCCGAA GTATCGACTC AACTATCAGA GGTAGTTGGC GTCATCGAGC GCCATCTCGA ACCGACGTTG CTGGCCGTAC ATTTGTACGG CTCCGCAGTG
12701 GATGGCGGCC TGAAGCCACA CAGTGATATT GATTTGCTGG TTACGGTGAC CGTAAGGCTT GATGAAACAA CGCGGCGAGC TTTGATCAAC GACCTTTTGG
12801 AAACTTCGGC TTCCCCTGGA GAGAGCGAGA TTCTCCGCGC TGTAGAAGTC ACCATTGTTG TGCACGACGA CATCATTCCG TGGCGTTATC CAGCTAAGCG
12901 CGAACTGCAA TTTGGAGAAT GGCAGCGCAA TGACATTCTT GCAGGTATCT TCGAGCCAGC CACGATCGAC ATTGATCTGG CTATCTTGCT GACAAAAGCA
13001 AGAGAACATA GCGTTGCCTT GGTAGGTCCA GCGGCGGAGG AACTCTTTGA TCCGGTTCCT GAACAGGATC TATTTGAGGC GCTAAATGAA ACCTTAACGC
13101 TATGGAACTC GCCGCCCGAC TGGGCTGGCG ATGAGCGAAA TGTAGTGCTT ACGTTGTCCC GCATTTGGTA CAGCGCAGTA ACCGGCAAAA TCGCGCCGAA
13201 GGATGTCGCT GCCGACTGGG CAATGGAGCG CCTGCCGGCC CAGTATCAGC CCGTCATACT TGAAGCTAGA CAGGCTTATC TTGGACAAGA AGAAGATCGC
13301 TTGGCCTCGC GCGCAGATCA GTTGGAAGAA TTTGTCCACT ACGTGAAAGG CGAGATCACC AAGGTAGTCG GCAAATAATG TCTAACAATT CGTTCAAGCC
13401 GACGCCGCTT CGCGGCGCGG CTTAACTCAA GCGTTAGATG CACTAAGCAC ATAATTGCTC ACAGCCAAAC TATCAGGTCA AGTCTGCTTT TATTATTTTT
13501 AAGCGTGCAT AATAAGCCCT ACACAAATTG GGAGATATAT CATGAAAGGC TGGCTTTTTC TTGTTATCGC AATAGTTGGC GAAGTAATCG CAACATAGCT
13601 TGCTTGGTCG TTCCGCGTGA ACGTCGGCTC GATTGTACCT GCGTTCAAAT ACTTTGCGAT CGTGTTGCGC GCCTGCCCGG TGCGTCGGCT GATCTCACGG
13701 ATCGACTGCT TCTCTCGCAA CGCCATCCGA CGGATGATGT TTAAAAGTCC CATGTGGATC ACTCCGTTGC CCCGTCGCTC ACCGTGTTGG GGGGAAGGTG
13801 CACATGGCTC AGTTCTCAAT GGAAATTATC TGCCTAACCG GCTCAGTTCT GCGTAGAAAC CAACATGCAA GCTCCACCGG GTGCAAAGCG GCAGCGGCGG
13901 CAGGATATAT TCAATTGTAA ATGGCTTCAT GTCCGGGAAA TCTACATGGA TCAGCAATGA GTATGATGGT CAATATGGAG AAAAAGAAAG AGTAATTACC
14001 AATTTTTTTT CAATTCAAAA ATGTAGATGT CCGCAGCGTT ATTATAAAAT GAAAGTACAT TTTGATAAAA CGACAAATTA CGATCCGTCG TATTTATAGG
14101 CGAAAGCAAT AAACAAATTA TTCTAATTCG GAAATCTTTA TTTCGACGTG TCTACATTCA CGTCCAAATG GGGGCTTAGA TGAGAAACTT CACGATCGGC

gfp-specific RGEN vector used in barley pSH92

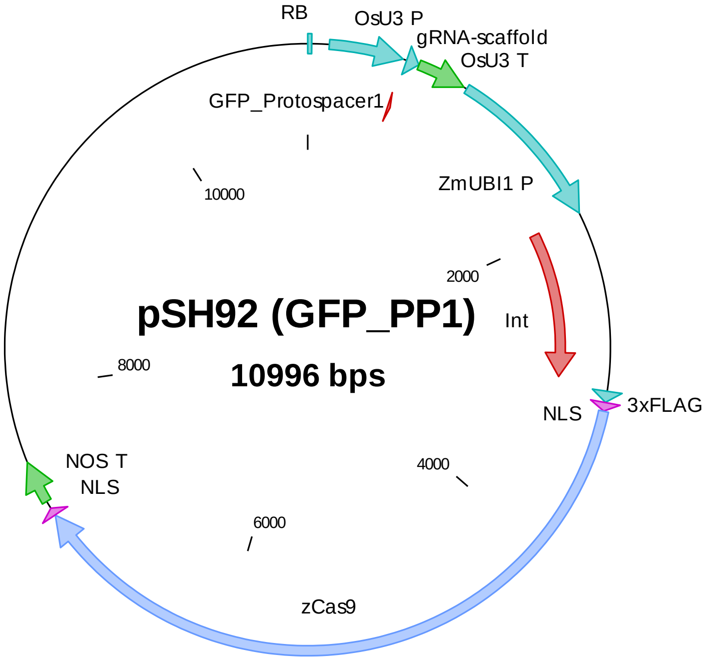


```
   1  GTTTACCCGC CAATATATCC TGTCAAACAC TGATAGTTTG TAGGCCCTTA AGGCCATGAA ACTGAAGGCG GGAAACGACA ATCTGATCCA AGCTCAAGCT
 101  AAGCTCACGT GACGGAATTA AGCTTAGTAA TTCATCCAGG TCACCAAGTT CTAGGATTTT CAGAACTGCA ACTTATTTTA TCAAGGAATC TTTAAACATA
 201  CGAACAGATC ACTTAAAGTT CTTCTGAAGC AACTTAAAGT TATCAGGCAT GCATGGATCT TGGAGGAATC AGATGTGCAG TCAGGGACCA TAGCACAAGA
 301  CAGGCGTCTT CTACTGGTGC TACCAGCAAA TGCTGGAAGC CGGGAACACT GGGTACGTTG GAAACCACGT GATGTGAAGA AGTAAGATAA ACTGTAGGAG
 401  AAAAGCATTT CGTAGTGGGC CATGAAGCCT TTCAGGACAT GTATTGCAGT ATGGGCCGGC CCATTACGCA ATTGGACGAC AACAAAGACT AGTATTAGTA
 501  CCACCTCGGC TATCCACATA GATCAAAGCT GATTTAAAAG AGTTGTGCAG ATGATCCGTG GCGTCTTTGC TCAGGGCGGA CTGTTTTAGA GCTAGAAATA
 601  GCAAGTTAAA ATAAGGCTAG TCCGTTATCA ACTTGAAAAA GTGGCACCGA GTCGGTGCTT TTTTTTTTCG TTTTGCATTG AGTTTTCTCC GTCGCATGTT
 701  TGCAGTTTTA TTTTCCGTTT TGCATTGAAA TTTCTCCGTC TCATGTTTGC AGCGTGTTCA AAAAGTACGC AGCTGTATTT CACTTATTTA CGGCGCCACA
 801  TTTTCATGCC GTTTGTGCCA ACTATCCCGA GCTAGTGAAT ACAGCTTGGC TTCACACAAC ACTGGTGACC CGCTGACCTG CTCGTACCTC GTACCGTCGT
 901  ACGGCACAGC ATTTGGAATT AAAGGGTGTG ATCGATACTG CTTGCTGCTA AGCTTGATAT CGAATTCCTG CAGTGCAGCG TGACCCGGTC GTGCCCCTCT
1001  CTAGAGATAA TGAGCATTGC ATGTCTAAGT TATAAAAAAT TACCACATAT TTTTTTTGTC ACACTTGTTT GAAGTGCAGT TTATCTATCT TTATACATAT
1101  ATTTAAACTT TACTCTACGA ATAATATAAT CTATAGTACT ACAATAATAT CAGTGTTTTA GAGAATCATA TAAATGAACA GTTAGACATG GTCTAAAGGA
1201  CAATTGAGTA TTTTGACAAC AGGACTCTAC AGTTTTATCT TTTTAGTGTG CATGTGTTCT CCTTTTTTTT TGCAAATAGC TTCACCTATA TAATACTTCA
1301  TCCATTTTAT TAGTACATCC ATTTAGGGTT TAGGGTTAAT GGTTTTTATA GACTAATTTT TTTAGTACAT CTATTTTATT CTATTTTAGC CTCTAAATTA
1401  AGAAAACTAA AACTCTATTT TAGTTTTTTT ATTTAATAAT TTAGATATAA AATAGAATAA AATAAAGTGA CTAAAAATTA AACAAATACC CTTTAAGAAA
1501  TTAAAAAAAC TAAGGAAACA TTTTTCTTGT TTCGAGTAGA TAATGCCAGC CTGTTAAACG CCGTCGACGA GTCTAACGGA CACCAACCAG CGAACCAGCA
1601  GCGTCGCGTC GGGCCAAGCG AAGCAGACGG CACGGCATCT CTGTCGCTGC CTCTGGACCC CTCTCGAGAG TTCCGCTCCA CCGTTGGACT TGCTCCGCTG
1701  TCGGCATCCA GAAATGCGTG GCGGAGCGGC AGACGTGAGC CGGCACGGCA GGCGGCCTCC TCCTCCTCTC ACGGCACGGC AGCTACGGGG GATTCCTTTC
1801  CCACCGCTCC TTCGCTTTCC CTTCCTCGCC CGCCGTAATA AATAGACACC CCCTCCACAC CCTCTTTCCC CAACCTCGTG TTGTTCGGAG CGCACACACA
1901  CACAACCAGA TCTCCCCCAA ATCCACCCGT CGGCACCTCC GCTTCAAGGT ACGCCGCTCG TCCTCCCCCC CCCCCCCTCT CTACCTTCTC TAGATCGGCG
2001  TTCCGGTCCA TGGTTAGGGC CCGGTAGTTC TACTTCTGTT CATGTTTGTG TTAGATCCGT GTTTGTGTTA GATCCGTGCT GCTAGCGTTC GTACACGGAT
2101  GCGACCTGTA CGTCAGACAC GTTCTGATTG CTAACTTGCC AGTGTTTCTC TTTGGGGAAT CCTGGGATGG CTCTAGCCGT TCCGCAGACG GGATCGATTT
2201  CATGATTTTT TTTGTTTCGT TGCATAGGGT TTGGTTTGCC CTTTTCCTTT ATTTCAATAT ATGCCGTGCA CTTGTTTGTC GGGTCATCTT TTCATGCTTT
2301  TTTTTGTCTT GGTTGTGATG ATGTGGTCTG GTTGGGCGGT CGTTCTAGAT CGGAGTAGAA TTCTGTTTCA AACTACCTGG TGGATTTATT AATTTTGGAT
2401  CTGTATGTGT GTGCCATACA TATTCATAGT TACGAATTGA AGATGATGGA TGGAAATATC GATCTAGGAT AGGTATACAT GTTGATGCGG GTTTTACTGA
2501  TGCATATACA GAGATGCTTT TTGTTCGCTT GGTTGTGATG ATGTGGTGTG GTTGGGCGGT CGTTCATTCG TTCTAGATCG GAGTAGAATA CTGTTTCAAA
2601  CTACCTGGTG TATTTATTAA TTTTGGAACT GTATGTGTGT GTCATACATC TTCATAGTTA CGAGTTTAAG ATGGATGGAA ATATCGATCT AGGATAGGTA
2701  TACATGTTGA TGTGGGTTTT ACTGATGCAT ATACATGATG GCATATGCAG CATCTATTCA TATGCTCTAA CCTTGAGTAC CTATCTATTA TAATAAACAA
2801  GTATGTTTTA TAATTATTTT GATCTTGATA TACTTGGATG ATGGCATATG CAGCAGCTAT ATGTGGATTT TTTTAGCCCT GCCTTCATAC GCTATTTATT
2901  TGCTTGGTAC TGTTTCTTTT GTCGATGCTC ACCCTGTTGT TTGGTGTTAC TTCTGCAGCC CGGGGGATCC CCAATACTTG TATGGCCGCG GCCGCTCTAG
3001  ATGGATTACA AGGACCACGA CGGGGATTAC AAGGACCACG ACATTGATTA CAAGGATGAT GATGACAAGA TGGCTCCGAA GAAGAAGAGG AAGGTTGGCA
3101  TCCACGGGGT GCCAGCTGCT GACAAGAAGT ACTCGATCGG CCTCGATATT GGGACTAACT CTGTTGGCTG GGCCGTGATC ACCGACGAGT ACAAGGTGCC
3201  CTCAAAGAAG TTCAAGGTCC TGGGCAACAC CGATCGGCAT TCCATCAAGA AGAATCTCAT TGGCGCTCTC CTGTTCGACA GCGGCGAGAC GGCTGAGGCT
3301  ACGCGGCTCA AGCGCACCGC CCGCAGGCGG TACACGCGCA GGAAGAATCG CATCTGCTAC CTGCAGGAGA TTTTCTCCAA CGAGATGGCG AAGGTTGACG
3401  ATTCTTTCTT CCACAGGCTG GAGGAGTCAT TCCTCGTGGA GGAGGATAAG AAGCACGAGC GGCATCCAAT CTTCGGCAAC ATTGTCGACG AGGTTGCCTA
3501  CCACGAGAAG TACCCTACGA TCTACCATCT GCGGAAGAAG CTCGTGGACT CCACAGATAA GGCGGACCTC CGCCTGATCT ACCTCGCTCT GGCCCACATG
3601  ATTAAGTTCA GGGGCCATTT CCTGATCGAG GGGGATCTCA ACCCGGACAA TAGCGATGTT GACAAGCTGT TCATCCAGCT CGTGCAGACG TACAACCAGC
3701  TCTTCGAGGA GAACCCCATT AATGCGTCAG GCGTCGACGC GAAGGCTATC CTGTCCGCTA GGCTCTCGAA GTCTCGGCGC CTCGAGAACC TGATCGCCCA
3801  GCTGCCGGGC GAGAAGAAGA ACGGCCTGTT CGGGAATCTC ATTGCGCTCA GCCTGGGGCT CACGCCCAAC TTCAAGTCGA ATTTCGATCT CGCTGAGGAC
3901  GCCAAGCTGC AGCTCTCCAA GGACACATAC GACGATGACC TGGATAACCT CCTGGCCCAG ATCGGCGATC AGTACGCGGA CCTGTTCCTC GCTGCCAAGA
4001  ATCTGTCGGA CGCCATCCTC CTGTCTGATA TTCTCAGGGT GAACACCGAG ATTACGAAGG CTCCGCTCTC AGCCTCCATG ATCAAGCGCT ACGACGAGCA
4101  CCATCAGGAT CTGACCCTCC TGAAGGCGCT GGTCAGGCAG CAGCTCCCCG AGAAGTACAA GGAGATCTTC TTCGATCAGT CGAAGAACGG CTACGCTGGG
```

```
4201  TACATTGACG GCGGGGCCTC TCAGGAGGAG TTCTACAAGT TCATCAAGCC GATTCTGGAG AAGATGGACG GCACGGAGGA GCTGCTGGTG AAGCTCAATC
4301  GCGAGGACCT CCTGAGGAAG CAGCGGACAT TCGATAACGG CAGCATCCCA CACCAGATTC ATCTCGGGGA GCTGCACGCT ATCCTGAGGA GGCAGGAGGA
4401  CTTCTACCCT TTCCTCAAGG ATAACCGCGA GAAGATCGAG AAGATTCTGA CTTTCAGGAT CCCGTACTAC GTCGGCCCAC TCGCTAGGGG CAACTCCCGC
4501  TTCGCTTGGA TGACCCGCAA GTCAGAGGAG ACGATCACGC CGTGGAACTT CGAGGAGGTG GTCGACAAGG GCGCTAGCGC TCAGTCGTTC ATCGAGAGGA
4601  TGACGAATTT CGACAAGAAC CTGCCAAATG AGAAGGTGCT CCCTAAGCAC TCGCTCCTGT ACGAGTACTT CACAGTCTAC AACGAGCTGA CTAAGGTGAA
4701  GTATGTGACC GAGGGCATGA GGAAGCCGGC TTTCCTGTCT GGGGAGCAGA AGAAGGCCAT CGTGGACCTC CTGTTCAAGA CCAACCGGAA GGTCACGGTT
4801  AAGCAGCTCA AGGAGGACTA CTTCAAGAAG ATTGAGTGCT TCGATTCGGT CGAGATCTCT GGCGTTGAGG ACCGCTTCAA CGCCTCCCTG GGGACCTACC
4901  ACGATCTCCT GAAGATCATT AAGGATAAGG ACTTCCTGGA CAACGAGGAG AATGAGGATA TCCTCGAGGA CATTGTGCTG ACACTCACTC TGTTCGAGGA
5001  CCGGGAGATG ATCGAGGAGC GCCTGAAGAC TTACGCCCAT CTCTTCGATG ACAAGGTCAT GAAGCAGCTC AAGAGGAGGA GGTACACCGG CTGGGGGAGG
5101  CTGAGCAGGA AGCTCATCAA CGGCATTCGG GACAAGCAGT CCGGGAAGAC GATCCTCGAC TTCCTGAAGA GCGATGGCTT CGCGAACCGC AATTTCATGC
5201  AGCTGATTCA CGATGACAGC CTCACATTCA AGGAGGATAT CCAGAAGGCT CAGGTGAGCG GCCAGGGGGA CTCGCTGCAC GAGCATATCG CGAACCTCGC
5301  TGGCTCGCCA GCTATCAAGA AGGGGATTCT GCAGACCGTG AAGGTTGTGG ACGAGCTGGT GAAGGTCATG GGCAGGCACA AGCCTGAGAA CATCGTCATT
5401  GAGATGGCCC GGGAGAATCA GACCACGCAG AAGGGCCAGA AGAACTCACG CGAGAGGATG AAGAGGATCG AGGAGGGCAT TAAGGAGCTG GGGTCCCAGA
5501  TCCTCAAGGA GCACCCGGTG GAGAACACGC AGCTGCAGAA TGAGAAGCTC TACCTGTACT ACCTCCAGAA TGGCCGCGAT ATGTATGTGG ACCAGGAGCT
5601  GGATATTAAC AGGCTCAGCG ATTACGACGT CGATCATATC GTTCCACAGT CATTCCTGAA GGATGACTCC ATTGACAACA AGGTCCTCAC CAGGTCGGAC
5701  AAGAACCGGG GCAAGTCTGA TAATGTTCCT TCAGAGGAGG TCGTTAAGAA GATGAAGAAC TACTGGCGCC AGCTCCTGAA TGCCAAGCTG ATCACGCAGC
5801  GGAAGTTCGA TAACCTCACA AAGGCTGAGA GGGGCGGGCT CTCTGAGCTG GACAAGGCGG GCTTCATCAA GAGGCAGCTG GTCGAGACAC GGCAGATCAC
5901  TAAGCACGTT GCGCAGATTC TCGACTCACG GATGAACACT AAGTACGATG AGAATGACAA GCTGATCCGC GAGGTGAAGG TCATCACCCT GAAGTCAAAG
6001  CTCGTCTCCG ACTTCAGGAA GGATTTCCAG TTCTACAAGG TTCGGGAGAT CAACAATTAC CACCATGCCC ATGACGCGTA CCTGAACGCG GTGGTCGGCA
6101  CAGCTCTGAT CAAGAAGTAC CCAAAGCTCG AGAGCGAGTT CGTGTACGGG GACTACAAGG TTTACGATGT GAGGAAGATG ATCGCCAAGT CGGAGCAGGA
6201  GATTGGCAAG GCTACCGCCA AGTACTTCTT CTACTCTAAC ATTATGAATT TCTTCAAGAC AGAGATCACT CTGGCCAATG GCGAGATCCG GAAGCGCCCC
6301  CTCATCGAGA CGAACGGCGA GACGGGGGAG ATCGTGTGGG ACAAGGGCAG GGATTTCGCG ACCGTCAGGA AGGTTCTCTC CATGCCACAA GTGAATATCG
6401  TCAAGAAGAC AGAGGTCCAG ACTGGCGGGT TCTCTAAGGA GTCAATTCTG CCTAAGCGGA ACAGCGACAA GCTCATCGCC CGCAAGAAGG ACTGGGATCC
6501  GAAGAAGTAC GGCGGGTTCG ACAGCCCCAC TGTGGCCTAC TCGGTCCTGG TTGTGGCGAA GGTTGAGAAG GGCAAGTCCA AGAAGCTCAA GAGCGTGAAG
6601  GAGCTGCTGG GGATCACGAT TATGGAGCGC TCCAGCTTCG AGAAGAACCC GATCGATTTC CTGGAGGCGA AGGGCTACAA GGAGGTGAAG AAGGACCTGA
6701  TCATTAAGCT CCCCAAGTAC TCACTCTTCG AGCTGGAGAA CGGCAGGAAG CGGATGCTGG CTTCCGCTGG CGAGCTGCAG AAGGGGAACG AGCTGGCTCT
6801  GCCGTCCAAG TATGTGAACT TCCTCTACCT GGCCTCCCAC TACGAGAAGC TCAAGGGCAG CCCCGAGGAC AACGAGCAGA AGCAGCTGTT CGTCGAGCAG
6901  CACAAGCATT ACCTCGACGA GATCATTGAG CAGATTTCCG AGTTCTCCAA GCGCGTGATC CTGGCCGACG CGAATCTGGA TAAGGTCCTC TCCGCGTACA
7001  ACAAGCACCG CGACAAGCCA ATCAGGGAGC AGGCTGAGAA TATCATTCAT CTCTTCACCC TGACGAACCT CGGCGCCCCT GCTGCTTTCA AGTACTTCGA
7101  CACAACTATC GATCGCAAGA GGTACACAAG CACTAAGGAG GTCCTGGACG CGACCCTCAT CCACCAGTCG ATTACCGGCC TCTACGAGAC GCGCATCGAC
7201  CTGTCTCAGC TCGGGGGCGA CAAGCGGCCA GCGGCGACGA AGAAGGCGGG GCAGGCGAAG AAGAAGAAGT GAGCTCAGGC CTCCATACAA GTATTGGGGA
7301  TCCGAATTTC CCCGATCGTT CAAACATTTG GCAATAAAGT TTCTTAAGAT TGAATCCTGT TGCCGGTCTT GCGATGATTA TCATATAATT TCTGTTGAAT
7401  TACGTTAAGC ATGTAATAAT TAACATGTAA TGCATGACGT TATTTATGAG ATGGGTTTTT ATGATTAGAG TCCCGCAATT ATACATTTAA TACGCGATAG
7501  AAAACAAAAT ATAGCGCGCA AACTAGGATA AATTATCGCG CGCGGTGTCA TCTATGTTAC TAGATCGGGA ATTCACTGGC CGTCGTTTTA CAACGTCGTG
7601  ACTGGGAAAA CCCTGGCGTT ACCCAACTTA ATCGCCTTGC AGCACATCCC CCTTTCGCCA GGGGTACCAG GCCGCCATGG CCAGGTACCG CCCCGTCCGG
7701  TCCTGCCCGT CACCGAGATT TGACTCGAGT TTCTCCATAA TAATGTGTGA GTAGTTCCCA GATAAGGGAA TTAGGGTTCC TATAGGGTTT CGCTCATGTG
7801  TTGAGCATAT AAGAAACCCT TAGTATGTAT TTGTATTTGT AAAATACTTC TATCAATAAA ATTTCTAATT CCTAAAACCA AAATCCAGTA CTAAAATCCA
7901  GATCCCCCGA ATTAATTCGG CGTTAATTCA GTACATTAAA AACGTCCGCA ATGTGTTATT AAGTTGTCAC TAGTCAGGTT AACTCAATTC GGCGTTAATT
8001  CAGTACATTA AAAACGTCCG CAATGTGTTA TTAAGTTGTC TAAGCGTCAA TTTGTTTACA CCACAATATA TCCTGCCACC AGCCAGCCAA CAGCTCCCCG
8101  ACCGGCAGCT CGGCACAAAA TCACCACTCG ATACAGGCAG CCCATCAGTC CGGGACGGCG TCAGCGGGAG AGCCGTTGTA AGGCGGCAGA CTTTGCTCAT
8201  GTTACCGATG CTATTCGGAA GAACGGCAAC TAAGCTGCCG GGTTTGAAAC ACGGATGATC TCGCGGAGGG TAGCATGTTG ATTGTAACGA TGACAGAGCG
8301  TTGCTGCCTG TGATCACTTA AGTAACTAAC TAACAGGAAG AGTTTGTAGA AACGCAAAAA GGCCATCCGT CAGGATGGCC TTCTGCTTAG TTTGATGCCT
8401  GGCAGTTTAT GGCGGGCGTC CTGCCCGCCA CCCTCCGGGC CGTTGCTTCA CAACGTTCAA ATCCGCTCCC GGCGGATTTG TCCTACTCAG GAGAGCGTTC
8501  ACCGACAAAC AACAGATAAA ACGAAAGGCC CAGTCTTCCG ACTGAGCCTT TCGTTTTATT TGATGCCTGG CAGTTCCCTA CTCTCGCTTA GTAGTTAGAC
8601  GTCCCCGAGA TCCATGCTAG ACCATGAATC CAGAAGCCCG AGAGGTTGCC GCCTTTCGGG CTTTTTCTTT TTCAAAAAAA AAAATTTATA AAACGATCTG
8701  TTGCGGCCGG CCGCCGGGTT GTGGGCAAAG GCGCTCGACG GTGGGCAACC GCTTGCGGTT GTCCACGGGC GGAGCCGGTG CGCGTAGCGC ATTGTCCACA
8801  AGCCAAGGGC GACCAATAAT TGATATATAT ATTCATAATT GAAAAGCTAA TTGAACATAC TACTTGCTGT AACTACTTGC CGGAGCGAGG GGTGTTTGCA
8901  AGCTGTTGAT CTGAAAGGGC TATTAGCGTT CTCACGTGCC TTTTTGATTA GCGATTTCAC GTGACCTTAT TAGCGATTTC ACGTACTCCG ATTAGCGATT
9001  TCACGTACCC TGATTAGCGA TTTCACGTGG ATAGTTTTTG GAGCGGGCCG GAAAGCCCCG TGAATCAAGG CTTTGCGGGG CATTAGCGGT TTCACGTGGA
9101  TAACTACCCT CTATCCACAG GCTTCCGGGG ATAAAAAAGC CCGCTCGACG GCGGGCTGTT GGATGGGGAT CTAGCGGTAA TACGGTTATC CACAGAATCA
9201  GGGGATAACG CAGGAAAGAA CATGTGAGCA AAAGGCCAGC AAAAGGCCAG GAACCGTAAA AAGGCCGCGT TGCTGGCGTT TTTCCATAGG CTCCGCCCCC
9301  CTGACGAGCA TCACAAAAAT CGACGCTCAA GTCAGAGGTG GCGAAACCCG ACAGGACTAT AAAGATACCA GGCGTTTCCC CCTGGAAGCT CCCTCGTGCG
9401  CTCTCCTGTT CCGACCCTGC CGCTTACCGG ATACCTGTCC GCCTTTCTCC CTTCGGGAAG CGTGGCGCTT TCTCATAGCT CACGCTGTAG GTATCTCAGT
9501  TCGGTGTAGG TCGTTCGCTC CAAGCTGGGC TGTGTGCACG AACCCCCCGT TCAGCCCGAC CGCTGCGCCT TATCCGGTAA CTATCGTCTT GAGTCCAACC
9601  CGGTAAGACA CGACTTATCG CCACTGGCAG CAGCCACTGG TAACAGGATT AGCAGAGCGA GGTATGTAGG CGGTGCTACA GAGTTCTTGA AGTGGTGGCC
9701  TAACTACGGC TACACTAGAA GAACAGTATT TGGTATCTGC GCTCTGCTGA AGCCAGTTAC CTTCGGAAAA AGAGTTGGTA GCTCTTGATC CGGCAAACAA
9801  ACCACCGCTG GTAGCGGTGG TTTTTTTGTT TGCAAGCAGC AGATTACGCG CAGAAAAAAA GGATCTCAAG AAGATCCTTT GATCTTTTCT ACCGGGTCTG
9901  ACGCTCAGTG GAACGGGGCC CAATCTGAAT AATGTTACAA CCAATTAACC AATTCTGATT AGAAAAACTC ATCGAGCATC AAATGAAACT GCAATTTATT
10001 CATATCAGGA TTATCAATAC CATATTTTTG AAAAAGCCGT TTCTGTAATG AAGGAGAAAA CTCACCGAGG CAGTTCCATA GGATGGCAAG ATCCTGGTAT
10101 CGGTCTGCGA TTCCGACTCG TCCAACATCA ATACAACCTA TTAATTTCCC CTCGTCAAAA ATAAGGTTAT CAAGTGAGAA ATCACCATGA GTGACGACTG
10201 AATCCGGTGA GAATGGCAAA AGTTTATGCA TTTCTTTCCA GACTTGTTCA ACAGGCCAGC CATTACGCTC GTCATCAAAA TCACTCGCAT CAACCAAACC
10301 GTTATTCATT CGTGATTGCG CCTGAGCGAG ACGAAATACG CGATCGCTGT TAAAAGGACA ATTACAAACA GGAATCGAAT GCAACCGGCG CAGGGACACT
10401 GCCAGCGCAT CAACAATATT TTCACCTGAA TCAGGATATT CTTCTAATAC CTGGAATGCT GTTTTTCCGG GGATCGCAGT GGTGAGTAAC CATGCATCAT
10501 CAGGAGTACG GATAAAATGC TTGATGGTCG GAAGAGGCAT AAATTCCGTC AGCCAGTTTA GTCTGACCAT CTCATCTGTA ACATCATTGG CAACGCTACC
10601 TTTGCCATGT TTCAGAAACA ACTCTGGCGC ATCGGGCTTC CCATACAAGC GATAGATTGT CGCACCTGAT TGCCCGACAT TATCGCGAGC CCATTTATAC
10701 CCATATAAAT CAGCATCCAT GTTGGAATTT AATCGCGGCC TCGACGTTTC CCGTTGAATA TGGCTCATAA CACCCCTTGT ATTACTGTTT ATGTAAGCAG
10801 ACAGTTTTAT TGTTCATGAT GATATATTTT TATCTTGTGC AATGTAACAT CAGAGATTTT GAGACACGGG CCAGAGCTGC AGTTTGATCC CGAGGGGAAC
10901 CCTGTGGTTG ACATGCACAT ACAAATGGAC GAACGGATAA ACCTTTTCAC GCCCTTTTAA ATATCCGTTA TTCTAATAAA CGCTCTTTTC TCTTAG
```

MLO-specific TALEN vector used in tobacco p114/115

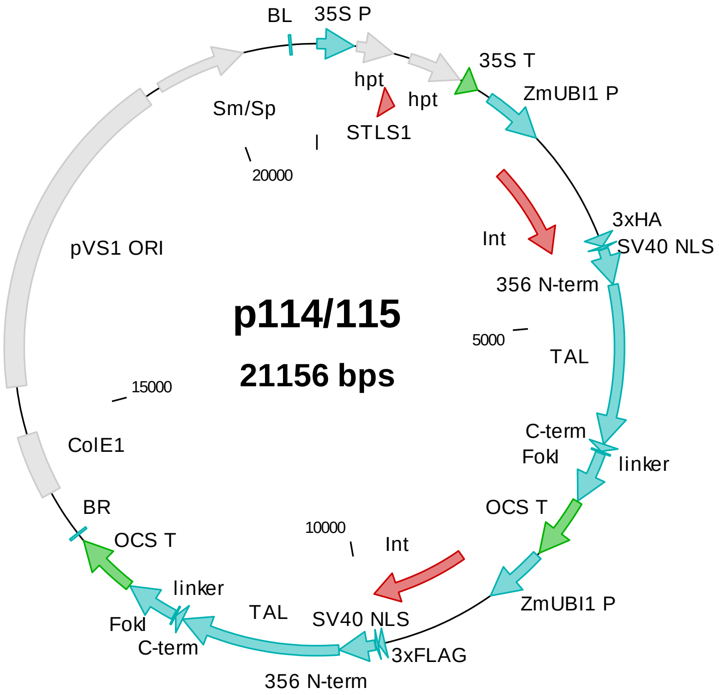


```
   1  ACATGGTGGA GCACGACACT CTCGTCTACT CCAAGAATAT CAAAGATACA GTCTCAGAAG ACCAGAGGGC TATTGAGACT TTTCAACAAA GGGTAATATC
 101  GGGAAACCTC CTCGGATTCC ATTGCCCAGC TATCTGTCAC TTCATCGAAA GGACAGTAGA AAAGGAAGAT GGCTTCTACA AATGCCATCA TTGCGATAAA
 201  GGAAAGGCTA TCGTTCAAGA TGCCTCTACC GACAGTGGTC CCAAAGATGG ACCCCCACCC ACGAGGAACA TCGTGGAAAA AGAAGACGTT CCAACCACGT
 301  CTTCAAAGCA AGTGGATTGA TGTGATATCT CCACTGACGT AAGGGATGAC GCACAATCCC ACTATCCTTC GCAAGACCCT TCCTCTATAT AAGGAAGTTC
 401  ATTTCATTTG GAGAGGACCT CGAGGGCCCC GGGGGGCAAT AAGATATGAA AAAGCCTGAA CTCACCGCGA CGTCTGTCGA GAAGTTTCTG ATCGAAAAGT
 501  TCGACAGCGT CTCCGACCTG ATGCAGCTCT CGGAGGGCGA AGAATCTCGT GCTTTCAGCT TCGATGTAGG AGGGCGTGGA TATGTCCTGC GGGTAAATAG
 601  CTGCGCCGAT GGTTTCTACA AAGATCGTTA TGTTTATCGG CACTTTGCAT CGGCCGCGCT CCCGATTCCG GAAGTGCTTG ACATTGGGGA ATTCAGCGAG
 701  AGCCTGACCT ATTGCATCTC CCGCCGTGCA CAGGGTGTCA CGTTGCAAGA CCTGCCTGAA ACCGAACTGC CCGCTGTTCT GCAGCCGGTC GCGGAGGCCA
 801  TGGATGCGAT CGCTGCGGCC GATCTTAGCC AGACGAGCGG GTTCGGCCCA TTCGGACCGC AAGGTAAGTT TCTGCTTCTA CCTTTGATAT ATATATAATA
 901  ATTATCATTA ATTAGTAGTA ATATAATATT TCAAATATTT TTTTCAAAAT AAAAGAATGT AGTATATAGC AATTGCTTTT CTGTAGTTTA TAAGTGTGTA
1001  TATTTTAATT TATAACTTTT CTAATATATG ACCAAAATTT GTTGATGTGC AGGTATCGGT CAATACACTA CATGGCGTGA TTTCATATGC GCGATTGCTG
1101  ATCCCCATGT GTATCACTGG CAAACTGTGA TGGACGACAC CGTCAGTGCG TCCGTCGCGC AGGCTCTCGA TGAGCTGATG CTTTGGGCCG AGGACTGCCC
1201  CGAAGTCCGG CACCTCGTGC ACGCGGATTT CGGCTCCAAC AATGTCCTGA CGGACAATGG CCGCATAACA GCGGTCATTG ACTGGAGCGA GGCGATGTTC
1301  GGGGATTCCC AATACGAGGT CGCCAACATC TTCTTCTGGA GGCCGTGGTT GGCTTGTATG GAGCAGCAGA CGCGCTACTT CGAGCGGAGG CATCCGGAGC
1401  TTGCAGGATC GCCGCGGCTC CGGGCGTATA TGCTCCGCAT TGGTCTTGAC CAACTCTATC AGAGCTTGGT TGACGGCAAT TTCGATGATG CAGCTTGGGC
1501  GCAGGGTCGA TGCGACGCAA TCGTCCGATC CGGAGCCGGG ACTGTCGGGC GTACACAAAT CGCCCGCAGA AGCGCGGCCG TCTGGACCGA TGGCTGTGTA
1601  GAAGTACTCG CCGATAGTGG AAACCGACGC CCCAGCACTC GTCCGAGGGC AAAGGAATAG AGTAGATGCC GACCGGAGTC CGCAAAAATC ACCAGTCTCT
1701  CTCTACAAAT CTATCTCTCT CTATTTTTCT CCAGAATAAT GTGTGAGTAG TTCCCAGATA AGGGAATTAG GGTTCTTATA GGGTTTCGCT CATGTGTTGA
1801  GCATATAAGA AACCCTTAGT ATGTATTTGT ATTTGTAAAA TACTTCTATC AATAAAATTT CTAATTCCTA AAACCAAAAT CCAGTGACCT GCAGGCATGC
1901  AAGCTGATCC ACTAGAGGCC ATGGCGGCCG CACTAGTGGA TCCCCCGGGA TGCATGAATT CAAGCTTATC GATGTCGACA GGCCTTAAGG GCCAGATCTT
2001  GGGCCCGGTA CCTGCCGAAT CGGATCCGGA GTTAACTGCA GTGCAGCGTG ACCCGGTCGT GCCCCTCTCT AGAGATAATG AGCATTGCAT GTCTAAGTTA
2101  TAAAAAATTA CCACATATTT TTTTTGTCAC ACTTGTTTGA AGTGCAGTTT ATCTATCTTT ATACATATAT TTAAACTTTA CTCTACGAAT AATATAATCT
2201  ATAGTACTAC AATAATATCA GTGTTTTAGA GAATCATATA AATGAACAGT TAGACATGGT CTAAAGGACA ATTGAGTATT TTGACAACAG GACTCTACAG
2301  TTTTATCTTT TTAGTGTGCA TGTGTTCTCC TTTTTTTTTG CAAATAGCTT CACCTATATA ATACTTCATC CATTTTATTA GTACATCCAC TTAGGGTTTA
2401  GGGTTAATGG TTTTTATAGA CTAATTTTTT TAGTACATCT ATTTTATTCT ATTTTAGCCT CTAAATTAAG AAAACTAAAA CTCTATTTTA GTTTTTTTAT
2501  TTAATAATTT AGATATAAAA TAGAATAAAA TAAAGTGACT AAAAATTAAA CAAATACCCT TTAAGAAATT AAAAAAACTA AGGAAACATT TTTCTTGTTT
2601  CGAGTAGATA ATGCCAGCCT GTTAAACGCC GTCGACGAGT CTAACGGACA CCAACCAGCG AACCAGCAGC GTCGCGTCGG GCCAAGCGAA GCAGACGGCA
2701  CGGCATCTCT GTCGCTGCCT CTGGACCCCT CTCGAGAGTT CCGCTCCACC GTTGGACTTG CTCCGCTGTC GGCATCCAGA AATTGCGTGG CGGAGCGGCA
2801  GACGTGAGCC GGCACGGCAG GCGGCCTCCT CCTCCTCTAA CGGCACGGCA GCTACGGGGG ATTCCTTTCC CACCGCTCCT TCGCTTTCCC TTCCTCGCCC
2901  GCCGTAATAA ATAGACACCC CCTCCACACC CTCTTTCCCC AACCTCGTGT TGTTCGGAGC GCACACACAC ACAACCAGAT CTCCCCCAAA TCCACCCGTC
3001  GGCACCTCCG CTTCAAGTAC GCCGCTCGTC CTCCCCCCCC CCCCCTCTCT ACCTTCTCTA GATCGGCGTT CCGGTCCATG GTTAGGGCCC GGTAGTTCTA
3101  CTTCTGTTCA TGTTTGTGTT AGATCCGTGT TTGTGTTAGA TCCGTGCTGC TAGCGTTCGT ACACGGATGC GACCTGTACG TCAGACACGT TCTGATTGCT
3201  AACTTGCCAG TGTTTCTCTT TGGGAATCCT GGGATGGCTC TAGCCGTTCC GCAGACGGGA TCGATTTCAT GATTTTTTTT GTTTCGTTGC ATATGGTTTG
3301  GTTTGCCCTT TTCCTTTATT TCAATATATG CCGTGCACTT GTTTGTCGGG TCATCTTTTC ATGCTTTTTT TTGTCTTGGT TGTGATGATG TGGTCTGGTT
3401  GGGCGGTCGT TCTAGATCGG AGTAGAATTC TGTTTCAAAC TACCTGGTGG ATTTATTAAT TTTGGATCTG TATGTGTGTG CCATACATAT TCATAGTTAC
3501  GAATTGAAGA TGATGGATGG AAATATCGAT CTAGGATAGG TATACATGTT GATGCGGGTT TTACTGATGC ATATACAGAG ATGCTTTTTG TTCGCTTGGT
3601  TGTGATGATG TGGTGTGGTT GGGCGGTCGT TCATTCGTTC TAGATCGGAG TAGAATACTG TTTCAAACTA CCTGGTGTAT TTATTAATTT TGGAACTGTA
3701  TGTGTGTGTC ATACATCTTC ATAGTTACGA GTTTAAGATG GATGGAAATA TCGATCTAGG ATAGGTATAC ATGTTGATGT GGGTTTTACT GATGCATATA
3801  CATGATGGCA TATGCAGCAT CTATTCATAT GCTCTAACCT TGAGTACCTA TCTATTATAA TAAACAAGTA TGTTTTATAA TTATTTTGAT CTTGATATAC
3901  TTGGATGATG GCATATGCAG CAGCTATATG TGGATTTTTT TAGCCCTGCC TTCATACGCT ATTTATTTGC TTGGTACTGT TTCTTTTGTC GATGCTCACC
4001  CTGTTGTTTG GTGTTACTTC TGCAGAATGG GGTTAATTAA CATCTTTTAC CCATACGATG TTCCTGACTA TGCGGGCTAT CCCTATGACG TCCCGGACTA
4101  TGCAGGATCC TATCCATATG ACGTTCCAGA TTACGCTGCT CAGTGCAGCC CCAAGAAAAA GCGGAAAGTG GGCATCCACG CTATGGATCT ACGCACGCTC
```

4201 GGCTACAGCC AGCAGCAACA GGAGAAGATC AAACCGAAGG TTCGTTCGAC AGTGGCGCAG CACCACGAGG CACTGGTCGG CCATGGGTTT ACACACGCGC
4301 ACATCGTTGC GCTCAGCCAA CACCCGGCAG CGTTAGGGAC CGTCGCTGTC AAGTATCAGG ACATGATCGC AGCGTTGCCA GAGGCGACAC ACGAAGCGAT
4401 CGTTGGCGTC GGCAAACAGT GGTCCGGCGC ACGCGCCCTG GAGGCCTTGC TCACGGTGGC GGGAGAGTTG AGAGGTCCAC CGTTACAGTT GGACACAGGC
4501 CAACTTCTCA AGATTGCAAA ACGTGGCGGC GTGACCGCAG TGGAGGCAGT GCATGCATGG CGCAATGCAC TGACGGGTGC CCCCCTGAAC CTTACGCCGC
4601 AGCAGGTGGT GGCCATCGCC AGCAATAATG GTGGCAAGCA GGCGCTGGAG ACGGTGCAGC GGCTGCTTCC GGTGCTGTGC CAGGCCCATG GCCTGACCCC
4701 GGAGCAGGTG GTGGCCATCG CCAGCAATAA TGGTGGCAAG CAGGCGCTGG AGACGGTGCA GCGATTGTTG CCGGTGCTGT GCCAGGCCCA TGGCCTGACC
4801 CCGGAGCAGG TGGTGGCCAT CGCCAGCAAT ATTGGTGGCA AGCAGGCGCT GGAGACTGTC CAGCGGCTGT TGCCGGTGCT GTGCCAGGCC CATGGCCTGA
4901 CCCCGGAGCA GGTGGTGGCC ATCGCCAGCA ATATTGGTGG CAAGCAGGCG CTTGAGACGG TGCAGCGGCT GTTGCCGGTG CTGTGCCAGG CCCATGGCCT
5001 GACCCCGCAG CAGGTGGTGG CCATCGCCAG CCACGACGGT GGCAAGCAGG CTCTGGAGAC GGTGCAGCGG CTGTTGCCGG TGCTGTGCCA GGCCCATGGC
5101 CTGACCCCGG AGCAGGTGGT GGCCATCGCC AGCAATATTG GGGGCAAGCA GGCGCTGGAG ACGGNGCAGC GGCTGTTGCC GGTGCTGTGC CAGGCCCATG
5201 GCCTGACCCC GCAGCAGGTG GTGGCCATCG CCAGCCACGA CGGCGGCAAG CAGGCGCTGG AGACGGTGCA GGCGCTGTTG CCGGTGCTGT GCCAGGCCCA
5301 TGGCCTGACC CCGGAGCAGG TGGTGGCCAT CGCAAGCAAT AATGGTGGCA AGCAGGCGCT GGAGACGGTG CAGGCGCTGT TGCCGGTGCT GTGCCAGGCC
5401 CATGGCCTGA CCCCGGAGCA GGTGGTGGCA ATCGCCAGCA ATAATGGTGG CAAGCAGGCG CTGGAGACGG TGCAGCGGCT GTTGCCGGTG CTGTGCCAGG
5501 CCCATGGCCT GACCCCGCAA CAGGTGGTAG CCATCGCCAG CCACGACGGT GGCAAGCAGG CGCTGGAGAC GGTGCAGCGG CTGTTGCCGG TGCTGTGCCA
5601 GGCCCATGGC CTGACACCCC AGCAGGTGGT AGCGATCGCC AGCCACGACG GTGGCAAGCA GGCGCTGGAG ACGGTGCAGC GGCTGCTTCC GGTGCTGTGC
5701 CAGGCCCATG GCCTGACCCC GCAGCAGGTG GTGGCCATCG CCAGCAATGG CGGTGGCAAG CAGGCGCTGG AGACGGTGCA GCGATTGTTG CCGGTGCTGT
5801 GCCAGGCCCA TGGCCTGACC CCGGAGCAGG TGGTGGCCAT CGCCAGCCAC GACGGTGGCA AGCAGGCGCT GGAGACTGTC CAGCGGCTGT TGCCGGTGCT
5901 GTGCCAGGCC CATGGCCTGA CCCCGGAGCA GGTGGTGGCC ATCGCCAGCC ACGACGGTGG CAAGCAGGCG CTTGAGACGG TGCAGCGGCT GTTGCCGGTG
6001 CTGTGCCAGG CCCATGGCCT GACCCCGGAG CAGGTGGTGG CCATCGCCAG CAATATTGGT GGCAAGCAGG CTCTGGAGAC GGTGCAGCGG CTGTTGCCGG
6101 TGCTGTGCCA GGCCCATGGC CTGACCCCGG AGCAGGTGGT GGCCATCGCC AGCCACGACG GGGGCAAGCA GGCGCTGGAG ACGGTGCAGC GGCTGTTGCC
6201 GGTGCTGTGC CAGGCCCATG GCCTGACCCC GCAGCAGGTG GTGGCCATCG CCAGCAATAT TGGCGGCAAG CAGGCGCTGG AGACGGTGCA GGCGCTGTTG
6301 CCGGCGCTGT GCCAGGCCCA TGGCCTGACA CCCCAGCAGG TGGTGGCCAT CGCCAGCAAT ATTGGCGGCA GGCCGGCGCT GGAGAGCATT GTTGCCCAGT
6401 TATCTCGCCC TGATCCGGCG TTGGCCGCGT TGACCAACGA CCACCTCGTC GCCTTGGCCT GCCTCGGCGG AAGCGGCGGA AGCGGTGGTC AGCTAGTGAA
6501 ATCTGAATTG GAAGAGAAGA AATCTGAACT TAGACATAAA TTGAAATATG TGCCACATGA ATATATTGAA TTGATTGAAA TCGCAAGAAA TTCAACTCAG
6601 GATAGAATCC TTGAAATGAA GGTGATGGAG TTCTTTATGA AGGTTTATGG TTATCGTGGT AAACATTTGG GTGGATCAAG GAAACCAGAC GGAGCAATTT
6701 ATACTGTCGG ATCTCCTATT GATTACGGTG TGATCGTTGA TACTAAGGCA TATTCAGGAG GTTATAATCT TCCAATTGGT CAAGCAGATG AAATGCAAAG
6801 ATATGTCGAA GAGAATCAAA CAAGAAACAA GCATATCAAC CCTAATGAAT GGTGGAAAGT CTATCCATCT TCAGTAACAG AATTTAAGTT CTTGTTTGTG
6901 AGTGGTCATT TCAAAGGAAA CTACAAAGCT CAGCTTACAA GATTGAATCA TATCACTAAT TGTAATGGAG CTGTTCTTAG TGTAGAAGAG CTTTTGATTG
7001 GTGGAGAAAT GATTAAAGCT GGTACATTGA CACTTGAGGA AGTGAGAAGG AAATTTAATA ACGGTGAGAT AAACTTTTAG GCTTGTCCTG CTTTAATGAG
7101 ATATGCGAGA AGCCTATGAT CGCATGATAT TTGCTTTCAA TTCTGTTGTG CACGTTGTAA AAAACCTGAG CATGTGTAGC TCAGATCCTT ACCGCCGGTT
7201 TCGGTTCATT CTAATGAATA TATCACCCGT TACTATCGTA TTTTTATGAA TAATATTCTC CGTTCAATTT ACTGATTGTA CCCTACTACT TATATGTACA
7301 ATATTAAAAT GAAAACAATA TATTGTGCTG AATAGGTTTA TAGCGACATC TATGATAGAG CGCCACAATA ACAAACAATT GCGTTTTATT ATTACAAATC
7401 CAATTTTAAA AAAAGCGGCA GAACCGGTCA AACCTAAAAG ACTGATTACA TAAATCTTAT TCAAATTTCA AAAGTGCCCC AGGGGCTAGT ATCTACGACA
7501 CACCGAGCGG CGAACTAATA ACGCTCACTG AAGGGAACTC CGGTTCCCCG CCGGCGCGCA TGGGTGAGAT TCCTTGAAGT TGAGTATTGG CCGTCCGCTC
7601 TACCGAAAGT TACGGGCACC ATTCAACCCG GTCCAGCACG GCGGCCGGGT AACCGACTTG CTGCCCCGAG AATTATGCAG CATTTTTTTG GTGTATGTGG
7701 GCCCCAAATG AAGTGCAGGT CAAACCTTGA CAGTGACGAC AAATCGTTGG GCGGGTCCAG GGCGAATTTT GCGACAACAT GTCGAGGCTC AGCAGGACCG
7801 CTGCAAGAAT CAAGCTTGGA GTTAACTGCA GTGCAGCGTG ACCCGGTCGT GCCCCTCTCT AGAGATAATG AGCATTGCAT GTCTAAGTTA TAAAAAATTA
7901 CCACATATTT TTTTTGTCAC ACTTGTTTGA AGTGCAGTTT ATCTATCTTT ATACATATAT TTAAACTTTA CTCTACGAAT AATATAATCT ATAGTACTAC
8001 AATAATATCA GTGTTTTAGA GAATCATATA AATGAACAGT TAGACATGGT CTAAAGGACA ATTGAGTATT TTGACAACAG GACTCTACAG TTTTATCTTT
8101 TTAGTGTGCA TGTGTTCTCC TTTTTTTTTG CAAATAGCTT CACCTATATA ATACTTCATC CATTTTATTA GTACATCCAC TTAGGGTTTA GGGTTAATGG
8201 TTTTTATAGA CTAATTTTTT TAGTACATCT ATTTTATTCT ATTTTAGCCT CTAAATTAAG AAAACTAAAA CTCTATTTTA GTTTTTTTAT TTAATAATTT
8301 AGATATAAAA TAGAATAAAA TAAAGTGACT AAAAATTAAA CAAATACCCT TTAAGAAATT AAAAAAACTA AGGAAACATT TTTCTTGTTT CGAGTAGATA
8401 ATGCCAGCCT GTTAAACGCC GTCGACGAGT CTAACGGACA CCAACCAGCG AACCAGCAGC GTCGCGTCGG GCCAAGCGAA GCAGACGGCA CGGCATCTCT
8501 GTCGCTGCCT CTGGACCCCT CTCGAGAGTT CCGCTCCACC GTTGGACTTG CTCCGCTGTC GGCATCCAGA AATTGCGTGG CGGAGCGGCA GACGTGAGCC
8601 GGCACGGCAG GCGGCCTCCT CCTCCTCTAA CGGCACGGCA GCTACGGGGG ATTCCTTTCC CACCGCTCCT TCGCTTTCCC TTCCTCGCCC GCCGTAATAA
8701 ATAGACACCC CCTCCACACC CTCTTTCCCC AACCTCGTGT TGTTCGGAGC GCACACACAC ACAACCAGAT CTCCCCCAAA TCCACCCGTC GGCACCTCCG
8801 CTTCAAGTAC GCCGCTCGTC CTCCCCCCCC CCCCCTCTCT ACCTTCTCTA GATCGGCGTT CCGGTCCATG GTTAGGGCCC GGTAGTTCTA CTTCTGTTCA
8901 TGTTTGTGTT AGATCCGTGT TTGTGTTAGA TCCGTGCTGC TAGCGTTCGT ACACGGATGC GACCTGTACG TCAGACACGT TCTGATTGCT AACTTGCCAG
9001 TGTTTCTCTT TGGGAATCCT GGGATGGCTC TAGCCGTTCC GCAGACGGGA TCGATTTCAT GATTTTTTTT GTTTCGTTGC ATATGGTTTG GTTTGCCCTT
9101 TTCCTTTATT TCAATATATG CCGTGCACTT GTTTGTCGGG TCATCTTTTC ATGCTTTTTT TTGTCTTGGT TGTGATGATG TGGTCTGGTT GGGCGGTCGT
9201 TCTAGATCGG AGTAGAATTC TGTTTCAAAC TACCTGGTGG ATTTATTAAT TTTGGATCTG TATGTGTGTG CCATACATAT TCATAGTTAC GAATTGAAGA
9301 TGATGGATGG AAATATCGAT CTAGGATAGG TATACATGTT GATGCGGGTT TTACTGATGC ATATACAGAG ATGCTTTTTG TTCGCTTGGT TGTGATGATG
9401 TGGTGTGGTT GGGCGGTCGT TCATTCGTTC TAGATCGGAG TAGAATACTG TTTCAAACTA CCTGGTGTAT TTATTAATTT TGGAACTGTA TGTGTGTGTC
9501 ATACATCTTC ATAGTTACGA GTTTAAGATG GATGGAAATA TCGATCTAGG ATAGGTATAC ATGTTGATGT GGGTTTTACT GATGCATATA CATGATGGCA
9601 TATGCAGCAT CTATTCATAT GCTCTAACCT TGAGTACCTA TCTATTATAA TAAACAAGTA TGTTTTATAA TTATTTTGAT CTTGATATAC TTGGATGATG
9701 GCATATGCAG CAGCTATATG TGGATTTTTT TAGCCCTGCC TTCATACGCT ATTTATTTGC TTGGTACTGT TTCTTTTGTC GATGCTCACC CTGTTGTTTG
9801 GTGTTACTTC TGCAGAATGG ACTACAAGGA TGACGATGAC AAGGACTACA AGGATGACGA TGACAAGGAC TACAAGGATG ACGATGACAA GCCCAAGAAA
9901 AAGCGGAAAG TGGGCATCCA CGCTATGGAT CTACGCACGC TCGGCTACAG CCAGCAGCAA CAGGAGAAGA TCAAACCGAA GGTTCGTTCG ACAGTGGCGC
10001 AGCACCACGA GGCACTGGTC GGCCATGGGT TTACACACGC GCACATCGTT GCGCTCAGCC AACACCCGGC AGCGTTAGGG ACCGTCGCTG TCAAGTATCA
10101 GGACATGATC GCAGCGTTGC CAGAGGCGAC ACACGAAGCG ATCGTTGGCG TCGGCAAACA GTGGTCCGGC GCACGCGCCC TGGAGGCCTT GCTCACGGTG
10201 GCGGGAGAGT TGAGAGGTCC ACCGTTACAG TTGGACACAG GCCAACTTCT CAAGATTGCA AAACGTGGCG GCGTGACCGC AGTGGAGGCA GTGCATGCAT
10301 GGCGCAATGC ACTGACGGGT GCCCCCCTGA ACCTTACGCC GCAGCAGGTG GTGGCCATCG CCAGCAATGG CGGTGGCAAG CAGGCGCTGG AGACGGTGCA
10401 GCGGCTGCTT CCGGTGCTGT GCCAGGCCCA TGGCCTGACC CCGGAGCAGG TGGTGGCCAT CGCCAGCAAT AATGGTGGCA AGCAGGCGCT GGAGACGGTG
10501 CAGCGATTGT TGCCGGTGCT GTGCCAGGCC CATGGCCTGA CCCCGGAGCA GGTGGTGGCC ATCGCCAGCA ATATTGGTGG CAAGCAGGCG CTGGAGACTG
10601 TCCAGCGGCT GTTGCCGGTG CTGTGCCAGG CCCATGGCCT GACCCCGGAG CAGGTGGTGG CCATCGCCAG CAATAATGGT GGCAAGCAGG CGCTTGAGAC
10701 GGTGCAGCGG CTGTTGCCGG TGCTGTGCCA GGCCCATGGC CTGACCCCGC AGCAGGTGGT GGCCATCGCC AGCCACGACG GTGGCAAGCA GGCTCTGGAG
10801 ACGGTGCAGC GGCTGTTGCC GGTGCTGTGC CAGGCCCATG GCCTGACCCC GGAGCAGGTG GTGGCCATCG CCAGCAATGG CGGGGGCAAG CAGGCGCTGG
10901 AGACGGTGCA GCGGCTGTTG CCGGTGCTGT GCCAGGCCCA TGGCCTGACC CCGCAGCAGG TGGTGGCCAT CGCCAGCAAT ATTGGCGGCA AGCAGGCGCT
11001 GGAGACGGTG CAGGCGCTGT TGCCGGTGCT GTGCCAGGCC CATGGCCTGA CCCCGGAGCA GGTGGTGGCC ATCGCAAGCA ATAATGGTGG CAAGCAGGCG
11101 CTGGAGACGG TGCAGGCGCT GTTGCCGGTG CTGTGCCAGG CCCATGGCCT GACCCCGGAG CAGGTGGTGG CAATCGCCAG CAATAATGGT GGCAAGCAGG
11201 CGCTGGAGAC GGTGCAGCGG CTGTTGCCGG TGCTGTGCCA GGCCCATGGC CTGACCCCGC AACAGGTGGT AGCCATCGCC AGCAATAATG GTGGCAAGCA
11301 GGCGCTGGAG ACGGTGCAGC GGCTGTTGCC GGTGCTGTGC CAGGCCCATG GCCTGACACC CCAGCAGGTG GTAGCGATCG CCAGCAATGG CGGTGGCAAG
11401 CAGGCGCTGG AGACGGTGCA GCGGCTGCTT CCGGTGCTGT GCCAGGCCCA TGGCCTGACC CCGGAGCAGG TGGTGGCCAT CGCCAGCAAT ATTGGTGGCA
11501 AGCAGGCGCT GGAGACGGTG CAGCGATTGT TGCCGGTGCT GTGCCAGGCC CATGGCCTGA CCCCGGAGCA GGTGGTGGCC ATCGCCAGCA ATATTGGTGG
11601 CAAGCAGGCG CTGGAGACTG TCCAGCGGCT GTTGCCGGTG CTGTGCCAGG CCCATGGCCT GACCCCGGAG CAGGTGGTGG CCATCGCCAG CCACGACGGT
11701 GGCAAGCAGG CGCTTGAGAC GGTGCAGCGG CTGTTGCCGG TGCTGTGCCA GGCCCATGGC CTGACCCCGC AGCAGGTGGT GGCCATCGCC AGCAATAATG
11801 GTGGCAAGCA GGCTCTGGAG ACGGTGCAGC GGCTGTTGCC GGTGCTGTGC CAGGCCCATG GCCTGACCCC GGAGCAGGTG GTGGCCATCG CCAGCAATAA
11901 TGGGGGCAAG CAGGCGCTGG AGACGGTGCA GCGGCTGTTG CCGGTGCTGT GCCAGGCCCA TGGCCTGACC CCGCAGCAGG TGGTGGCCAT CGCCAGCAAT
12001 AATGGCGGCA AGCAGGCGCT GGAGACGGTG CAGGCGCTGT TGCCGGCGCT GTGCCAGGCC CATGGCCTGA CACCCCAGCA GGTGGTGGCC ATCGCCAGCA
12101 ATATTGGCGG CAGGCCGGCG CTGGAGAGCA TTGTTGCCCA GTTATCTCGC CCTGATCCGG CGTTGGCCGC GTTGACCAAC GACCACCTCG TCGCCTTGGC
12201 CTGCCTCGGC GGAAGCGGCG GAAGCGGTGG TCAGCTAGTG AAATCTGAAT TGGAAGAGAA GAAATCTGAA CTTAGACATA AATTGAAATA TGTGCCACAT
12301 GAATATATTG AATTGATTGA AATCGCAAGA AATTCAACTC AGGATAGAAT CCTTGAAATG AAGGTGATGG AGTTCTTTAT GAAGGTTTAT GGTTATCGTG
12401 GTAAACATTT GGGTGGATCA AGGAAACCAG ACGGAGCAAT TTATACTGTC GGATCTCCTA TTGATTACGG TGTGATCGTT GATACTAAGG CATATTCAGG
12501 AGGTTATAAT CTTCCAATTG GTCAAGCAGA TGAAATGCAA AGATATGTCG AAGAGAATCA AACAAGAAAC AAGCATATCA ACCCTAATGA ATGGTGGAAA
12601 GTCTATCCAT CTTCAGTAAC AGAATTTAAG TTCTTGTTTG TGAGTGGTCA TTTCAAAGGA AACTACAAAG CTCAGCTTAC AAGATTGAAT CATATCACTA
12701 ATTGTAATGG AGCTGTTCTT AGTGTAGAAG AGCTTTTGAT TGGTGGAGAA ATGATTAAAG CTGGTACATT GACACTTGAG GAAGTGAGAA GGAAATTTAA
12801 TAACGGTGAG ATAAACTTTT AGGCTTGTCC TGCTTTAATG AGATATGCGA GAAGCCTATG ATCGCATGAT ATTTGCTTTC AATTCTGTTG TGCACGTTGT
12901 AAAAAACCTG AGCATGTGTA GCTCAGATCC TTACCGCCGG TTTCGGTTCA TTCTAATGAA TATATCACCC GTTACTATCG TATTTTTATG AATAATATTC
13001 TCCGTTCAAT TTACTGATTG TACCCTACTA CTTATATGTA CAATATTAAA ATGAAAACAA TATATTGTGC TGAATAGGTT TATAGCGACA TCTATGATAG
13101 AGCGCCACAA TAACAAACAA TTGCGTTTTA TTATTACAAA TCCAATTTTA AAAAAAGCGG CAGAACCGGT CAAACCTAAA AGACTGATTA CATAAATCTT
13201 ATTCAAATTT CAAAAGTGCC CCAGGGGCTA GTATCTACGA CACACCGAGC GGCGAACTAA TAACGCTCAC TGAAGGGAAC TCCGGTTCCC CGCCGGCGCG
13301 CATGGGTGAG ATTCCTTGAA GTTGAGTATT GGCCGTCCGC TCTACCGAAA GTTACGGGCA CCATTCAACC CGGTCCAGCA CGGCGGCCGG GTAACCGACT
13401 TGCTGCCCCG AGAATTATGC AGCATTTTTT TGGTGTATGT GGGCCCCAAA TGAAGTGCAG GTCAAACCTT GACAGTGACG ACAAATCGTT GGGCGGGTCC
13501 AGGGCGAATT TTGCGACAAC ATGTCGAGGC TCAGCAGGAC CGCTACTAGA GGATGCACAT GTGACCGAGG GAGGTACCCG ATCAGATTGT CGTTTCCCGC
13601 CTTCGGTTTA AACTATCAGT GTTTGACAGG ATATATTGGC GGGTAAACCT AAGAGAAAAG AGCGTTTATT AGAATAATCG GATATTTAAA AGGGCGTGAA
13701 AAGGTTTATC CGTTCGTCCA TTTGTATGTG CATGCCAACC ACAGGGTTCC CCTCGGGAGT GCTTGGCATT CCGTGCGATA ATGACTTCTG TTCAACCACC
13801 CAAACGTCGG AAAGCCTGAC GACGGAGCAG CATTCCAAAA AGATCCCTTG GCTCGTCTGG GTCGGCTAGA AGGTCGAGTG GGCTGCTGTG GCTTGATCCC
13901 TCAACGCGGT CGCGGACGTA GCGCAGCGCC GAAAAATCCT CGATCGCAAA TCCGACGCTG TCGAAAAGCG TGATCTGCTT GTCGCTCTTT CGGCCGACGT
14001 CCTGGCCAGT CATCACGCGC CAAAGTTCCG TCACAGGATG ATCTGGCGCG AGTTGCTGGA TCTCGCCTTC AATCCGGGTC TGTGGCGGGA ACTCCACGAA
14101 AATATCCGAA CGCAGCAAGA TATCGCGGTG CATCTCGGTC TTGCCTGGGC AGTCGCCGCC GACGCCGTTG ATGTGGACGC CGAAAAGGAT CTAGGTGAAG
14201 ATCCTTTTTG ATAATCTCAT GACCAAAATC CCTTAACGTG AGTTTTCGTT CCACTGAGCG TCAGACCCCG TAGAAAAGAT CAAAGGATCT TCTTGAGATC
14301 CTTTTTTTCT GCGCGTAATC TGCTGCTTGC AAACAAAAAA ACCACCGCTA CCAGCGGTGG TTTGTTTGCC GGATCAAGAG CTACCAACTC TTTTTCCGAA
14401 GGTAACTGGC TTCAGCAGAG CGCAGATACC AAATACTGTT CTTCTAGTGT AGCCGTAGTT AGGCCACCAC TTCAAGAACT CTGTAGCACC GCCTACATAC
14501 CTCGCTCTGC TAATCCTGTT ACCAGTGGCT GCTGCCAGTG GCGATAAGTC GTGTCTTACC GGGTTGGACT CAAGACGATA GTTACCGGAT AAGGCGCAGC
14601 GGTCGGGCTG AACGGGGGGT TCGTGCACAC AGCCCAGCTT GGAGCGAACG ACCTACACCG AACTGAGATA CCTACAGCGT GAGCTATGAG AAAGCGCCAC

14701 GCTTCCCGAA GGGAGAAAGG CGGACAGGTA TCCGGTAAGC GGCAGGGTCG GAACAGGAGA GCGCACGAGG GAGCTTCCAG GGGGAAACGC CTGGTATCTT
14801 TATAGTCCTG TCGGGTTTCG CCACCTCTGA CTTGAGCGTC GATTTTTGTG ATGCTCGTCA GGGGGGCGGA GCCTATGGAA AAACGCCAGC AACGCGGCCT
14901 TTTTACGGTT CCTGGCCTTT TGCTGGCCTT TTGCTCACAT GTTCTTTCCT GCGTTATCCC CTGATTCTGT GGATAACCGA TTACCGCCTT TGAGTGAGCT
15001 GATACCGCTC GCCGCAGCCG AACGACCGAG CGCAGCGAGT CAGTGAGCGA GGAAGCGGAA GAGCGCCTGA TGCGGTATTT TCTCCTTACG CATCTGTGCG
15101 GTATTTCACA CCGCATATGG TGCACTCTCA GTACAATCTG CTCTGATGCC GCATAGTTAA GCCAGTATAC ACTCCGCTAT CGCTACGTGA CTGGGTCATG
15201 GCTGCGCCCC GACACCCGCC AACACCCGCT GACGCGCCCT GACGGGCTTG TCTGCTCCCG GCATCCGCTT ACAGACAAGC TGTGACCGTC TCCGGGAGCT
15301 GCATGTGTCA GAGGTTTTCA CCGTCATCAC CGAAACGCGC GAGGCAGGGG TACGTCGAGG TCGATCCAAC CCCTCCGCTG CTATAGTGCA GTCGGCTTCT
15401 GACGTTCAGT GCAGCCGTCT TCTGAAAACG ACATGTCGCA CAAGTCCTAA GTTACGCGAC AGGCTGCCGC CCTGCCCTTT TCCTGGCGTT TTCTTGTCGC
15501 GTGTTTTAGT CGCATAAAGT AGAATACTTG CGACTAGAAC CGGAGACATT ACGCCATGAA CAAGAGCGCC GCCGCTGGCC TGCTGGGCTA TGCCCGCGTC
15601 AGCACCGACG ACCAGGACTT GACCAACCAA CGGGCCGAAC TGCACGCGGC CGGCTGCACC AAGCTGTTTT CCGAGAAGAT CACCGGCACC AGGCGCGACC
15701 GCCCGGAGCT GGCCAGGATG CTTGACCACC TACGCCCTGG CGACGTTGTG ACAGTGACCA GGCTAGACCG CCTGGCCCGC AGCACCCGCG ACCTACTGGA
15801 CATTGCCGAG CGCATCCAGG AGGCCGGCGC GGGCCTGCGT AGCCTGGCAG AGCCGTGGGC CGACACCACC ACGCCGGCCG GCCGCATGGT GTTGACCGTG
15901 TTCGCCGGCA TTGCCGAGTT CGAGCGTTCC CTAATCATCG ACCGCACCCG GAGCGGGCGC GAGGCCGCCA AGGCGCGAGG CGTGAAGTTT GGCCCCCGCC
16001 CTACCCTCAC CCCGGCACAG ATCGCGCACG CCCGCGAGCT GATCGACCAG GAAGGCCGCA CCGTGAAAGA GGCGGCTGCA CTGCTTGGCG TGCATCGCTC
16101 GACCCTGTAC CGCGCACTTG AGCGCAGCGA GGAAGTGACG CCCACCGAGG CCAGGCGGCG CGGTGCCTTC CGTGAGGACG CATTGACCGA GGCCGACGCC
16201 CTGGCGGCCG CCGAGAATGA ACGCCAAGAG GAACAAGCAT GAAACCGCAC CAGGACGGCC AGGACGAACC GTTTTTCATT ACCGAAGAGA TCGAGGCGGA
16301 GATGATCGCG GCCGGGTACG TGTTCGAGCC GCCCGCGCAC GTCTCAACCG TGCGGCTGCA TGAAATCCTG GCCGGTTTGT CTGATGCCAA GCTCGCGGCC
16401 TGGCCGGCGA GCTTGGCCGC TGAAGAAACC GAGCGCCGCC GTCTAAAAAG GTGATGTGTA TTTGAGTAAA ACAGCTTGCG TCATGCGGTC GCTGCGTATA
16501 TGATGCGATG AGTAAATAAA CAAATACGCA AGGGGAACGC ATGAAGGTTA TCGCTGTACT TAACCAGAAA GGCGGGTCAG GCAAGACGAC CATCGCAACC
16601 CATCTAGCCC GCGCCCTGCA ACTCGCCGGG GCCGATGTTC TGTTAGTCGA TTCCGATCCC CAGGGCAGTG CCCGCGATTG GGCGGCCGTG CGGGAAGATC
16701 AACCGCTAAC CGTTGTCGGC ATCGACCGCC CGACGATTGA CCGCGACGTG AAGGCCATCG GCCGGCGCGA CTTCGTAGTG ATCGACGGAG CGCCCCAGGC
16801 GGCGGACTTG GCTGTGTCCG CGATCAAGGC AGCCGACTTC GTGCTGATTC CGGTGCAGCC AAGCCCTTAC GACATATGGG CCACCGCCGA CCTGGTGGAG
16901 CTGGTTAAGC AGCGCATTGA GGTCACGGAT GGAAGGCTAC AAGCGGCCTT TGTCGTGTCG CGGGCGATCA AAGGCACGCG CATCGGCGGT GAGGTTGCCG
17001 AGGCGCTGGC CGGGTACGAG CTGCCCATTC TTGAGTCCCG TATCACGCAG CGCGTGAGCT ACCCAGGCAC TGCCGCCGCC GGCACAACCG TTCTTGAATC
17101 AGAACCCGAG GGCGACGCTG CCCGCGAGGT CCAGGCGCTG GCCGCTGAAA TTAAATCAAA ACTCATTTGA GTTAATGAGG TAAAGAGAAA ATGAGCAAAA
17201 GCACAAACAC GCTAAGTGCC GGCCGTCCGA GCGCACGCAG CAGCAAGGCT GCAACGTTGG CCAGCCTGGC AGACACGCCA GCCATGAAGC GGGTCAACTT
17301 TCAGTTGCCG GCGGAGGATC ACACCAAGCT GAAGATGTAC GCGGTACGCC AAGGCAAGAC CATTACCGAG CTGCTATCTG AATACATCGC GCAGCTACCA
17401 GAGTAAATGA GCAAATGAAT AAATGAGTAG ATGAATTTTA GCGGCTAAAG GAGGCGGCAT GGAAAATCAA GAACAACCAG GCACCGACGC CGTGGAATGC
17501 CCCATGTGTG GAGGAACGGG CGGTTGGCCA GGCGTAAGCG GCTGGGTTGT CTGCCGGCCC TGCAATGGCA CTGGAACCCC CAAGCCCGAG GAATCGGCGT
17601 GAGCGGTCGC AAACCATCCG GCCCGGTACA AATCGGCGCG GCGCTGGGTG ATGACCTGGT GGAGAAGTTG AAGGCGGCGC AGGCCGCCCA GCGGCAACGC
17701 ATCGAGGCAG AAGCACGCCC CGGTGAATCG TGGCAAGCGG CCGCTGATCG AATCCGCAAA GAATCCCGGC AACCGCCGGC AGCCGGTGCG CCGTCGATTA
17801 GGAAGCCGCC CAAGGGCGAC GAGCAACCAG ATTTTTTCGT TCCGATGCTC TATGACGTGG GCACCCGCGA TAGTCGCAGC ATCATGGACG TGGCCGTTTT
17901 CCGTCTGTCG AAGCGTGACC GACGAGCTGG CGAGGTGATC CGCTACGAGC TTCCAGACGG GCACGTAGAG GTTTCCGCAG GGCCGGCCGG CATGGCGAGT
18001 GTGTGGGATT ACGACCTGGT ACTGATGGCG GTTTCCCATC TAACCGAATC CATGAACCGA TACCGGGAAG GGAAGGGAGA CAAGCCCGGC CGCGTGTTCC
18101 GTCCACACGT TGCGGACGTA CTCAAGTTCT GCCGGCGAGC CGATGGCGGA AAGCAGAAAG ACGACCTGGT AGAAACCTGC ATTCGGTTAA ACACCACGCA
18201 CGTTGCCATG CAGCGTACGA AGAAGGCCAA GAACGGCCGC CTGGTGACGG TATCCGAGGG TGAAGCCTTG ATTAGCCGCT ACAAGATCGT AAAGAGCGAA
18301 ACCGGGCGGC CGGAGTACAT CGAGATCGAG CTAGCTGATT GGATGTACCG CGAGATCACA GAAGGCAAGA ACCCGGACGT GCTGACGGTT CACCCCGATT
18401 ACTTTTTGAT CGATCCCGGC ATCGGCCGTT TTCTCTACCG CCTGGCACGC CGCGCCGCAG GCAAGGCAGA AGCCAGATGG TTGTTCAAGA CGATCTACGA
18501 ACGCAGTGGC AGCGCCGGAG AGTTCAAGAA GTTCTGTTTC ACCGTGCGCA AGCTGATCGG GTCAAATGAC CTGCCGGAGT ACGATTTGAA GGAGGAGGCG
18601 GGGCAGGCTG GCCCGATCCT AGTCATGCGC TACCGCAACC TGATCGAGGG CGAAGCATCC GCCGGTTCCT AATGTACGGA GCAGATGCTA GGGCAAATTG
18701 CCCTAGCAGG GGAAAAAGGT CGAAAAGGTC TCTTTCCTGT GGATAGCACG TACATTGGGA ACCCAAAGCC GTACATTGGG AACCGGAACC CGTACATTGG
18801 GAACCCAAAG CCGTACATTG GGAACCGGTC ACACATGTAA GTGACTGATA TAAAAGAGAA AAAAGGCGAT TTTTCCGCCT AAAACTCTTT AAAACTTATT
18901 AAAACTCTTA AAACCCGCCT GGCCTGTGCA TAACTGTCTG GCCAGCGCAC AGCCGAAGAG CTGCAAAAAG CGCCTACCCT TCGGTCGCTG CGCTCCCTAC
19001 GCCCCGCCGC TTCGCGTCGG CCTATCGCGG CCGCTGGCCG CTCAAAAATG GCTGGCCTAC GGCCAGGCAA TCTACCAGGG CGCGGACAAG CCGCGCCGTC
19101 GCCACTCGAC CGCCGGCGCC CACATCAAGG CACCGGTGGG TATGCCTGAC GATGCGTGGA GACCGAAACC TTGCGCTCGT TCGCCAGCCA GGACAGAAAT
19201 GCCTCGACTT CGCTGCTGCC CAAGGTTGCC GGGTGACGCA CACCGTGGAA ACGGATGAAG GCACGAACCC AGTGGACATA AGCCTGTTCG GTTCGTAAGC
19301 TGTAATGCAA GTAGCGTATG CGCTCACGCA ACTGGTCCAG AACCTTGACC GAACGCAGCG GTGGTAACGG CGCAGTGGCG GTTTTCATGG CTTGTTATGA
19401 CTGTTTTTTT GGGGTACAGT CTATGCCTCG GGCATCCAAG CAGCAAGCGC GTTACGCCGT GGGTCGATGT TTGATGTTAT GGAGCAGCAA CGATGTTACG
19501 CAGCAGGGCA GTCGCCCTAA AACAAAGTTA AACATCATGA GGGAAGCGGT GATCGCCGAA GTATCGACTC AACTATCAGA GGTAGTTGGC GTCATCGAGC
19601 GCCATCTCGA ACCGACGTTG CTGGCCGTAC ATTTGTACGG CTCCGCAGTG GATGGCGGCC TGAAGCCACA CAGTGATATT GATTTGCTGG TTACGGTGAC
19701 CGTAAGGCTT GATGAAACAA CGCGGCGAGC TTTGATCAAC GACCTTTTGG AAACTTCGGC TTCCCCTGGA GAGAGCGAGA TTCTCCGCGC TGTAGAAGTC
19801 ACCATTGTTG TGCACGACGA CATCATTCCG TGGCGTTATC CAGCTAAGCG CGAACTGCAA TTTGGAGAAT GGCAGCGCAA TGACATTCTT GCAGGTATCT
19901 TCGAGCCAGC CACGATCGAC ATTGATCTGG CTATCTTGCT GACAAAAGCA AGAGAACATA GCGTTGCCTT GGTAGGTCCA GCGGCGGAGG AACTCTTTGA
20001 TCCGGTTCCT GAACAGGATC TATTTGAGGC GCTAAATGAA ACCTTAACGC TATGGAACTC GCCGCCCGAC TGGGCTGGCG ATGAGCGAAA TGTAGTGCTT
20101 ACGTTGTCCC GCATTTGGTA CAGCGCAGTA ACCGGCAAAA TCGCGCCGAA GGATGTCGCT GCCGACTGGG CAATGGAGCG CCTGCCGGCC CAGTATCAGC
20201 CCGTCATACT TGAAGCTAGA CAGGCTTATC TTGGACAAGA AGAAGATCGC TTGGCCTCGC GCGCAGATCA GTTGGAAGAA TTTGTCCACT ACGTGAAAGG
20301 CGAGATCACC AAGGTAGTCG GCAAATAATG TCTAACAATT CGTTCAAGCC GACGCCGCTT CGCGGCGCGG CTTAACTCAA GCGTTAGATG CACTAAGCAC
20401 ATAATTGCTC ACAGCCAAAC TATCAGGTCA AGTCTGCTTT TATTATTTTT AAGCGTGCAT AATAAGCCCT ACACAAATTG GGAGATATAT CATGAAAGGC
20501 TGGCTTTTTC TTGTTATCGC AATAGTTGGC GAAGTAATCG CAACATAGCT TGCTTGGTCG TTCCGCGTGA ACGTCGGCTC GATTGTACCT GCGTTCAAAT
20601 ACTTTGCGAT CGTGTTGCGC GCCTGCCCGG TGCGTCGGCT GATCTCACGG ATCGACTGCT TCTCTCGCAA CGCCATCCGA CGGATGATGT TTAAAAGTCC
20701 CATGTGGATC ACTCCGTTGC CCCGTCGCTC ACCGTGTTGG GGGGAAGGTG CACATGGCTC AGTTCTCAAT GGAAATTATC TGCCTAACCG GCTCAGTTCT
20801 GCGTAGAAAC CAACATGCAA GCTCCACCGG GTGCAAAGCG GCAGCGGCGG CAGGATATAT TCAATTGTAA ATGGCTTCAT GTCCGGGAAA TCTACATGGA
20901 TCAGCAATGA GTATGATGGT CAATATGGAG AAAAAGAAAG AGTAATTACC AATTTTTTTT CAATTCAAAA ATGTAGATGT CCGCAGCGTT ATTATAAAAT
21001 GAAAGTACAT TTTGATAAAA CGACAAATTA CGATCCGTCG TATTTATAGG CGAAAGCAAT AAACAAATTA TTCTAATTCG GAAATCTTTA TTTCGACGTG
21101 TCTACATTCA CGTCCAAATG GGGGCTTAGA TGAGAAACTT CACGATCGGC TCTAGA
